# Amycolatomycins A and B, Cyclic Hexapeptides Isolated from an *Amycolatopsis* sp. 195334CR

**DOI:** 10.3390/antibiotics10030261

**Published:** 2021-03-05

**Authors:** Gian Primahana, Chandra Risdian, Tjandrawati Mozef, Joachim Wink, Frank Surup, Marc Stadler

**Affiliations:** 1 Department Microbial Drugs, Helmholtz Centre for Infection Research GmbH (HZI), Inhoffenstrasse 7, 38124 Braunschweig, Germany; Gian.Primahana@helmholtz-hzi.de (G.P.); Frank.Surup@helmholtz-hzi.de (F.S.); 2 Research Center for Chemistry, Indonesian Institute of Sciences (LIPI), Kawasan Puspiptek, Serpong, Tangerang Selatan 15314, Indonesia; tjandrawm@gmail.com; 3 Working Group Microbial Strain Collection, Helmholtz Centre for Infection Research GmbH (HZI), Inhoffenstrasse 7, 38124 Braunschweig, Germany; Chandra.Risdian@helmholtz-hzi.de (C.R.); Joachim.Wink@helmholtz-hzi.de (J.W.); 4 Research Unit for Clean Technology, Indonesian Institute of Sciences (LIPI), Bandung 40135, Indonesia

**Keywords:** rare Actinobacteria, *Amycolatopsis* sp., new secondary metabolites, peptides, 2,6-dichloro-tryptophan, amycolatomycins

## Abstract

The rare actinobacterium *Amycolatopsis* sp. strain 195334CR was found to produce previously undescribed cyclic hexapeptides, which we named amycolatomycin A and B (**1** and **2**). Their planar structures were determined by high-resolution mass spectrometry as well as extensive 1D and 2D NMR spectroscopy, while the absolute stereochemistry of its amino acids were determined by Marfey’s method. Moreover, **1** and **2** differ by the incorporation of l-Ile and l-*allo*-Ile, respectively, whose FDVA (Nα-(2,4-Dinitro-5-fluorphenyl)-L-valinamide) derivatives were separated on a C_4_ column. Their hallmark in common is a unique 2,6-dichloro-tryptophan amino acid unit. Amycolatomycin A (**1**) exhibited weak activity against *Bacillus subtilis* DSM 10 (minimum inhibitory concentration (MIC) = 33.4 µg/mL).

## 1. Introduction

The misuse and overuse of antibiotics have serious consequences of the emergence of antibiotics resistance against currently used antibiotics. This situation led to the urgently needed novel antimicrobial compounds, especially with new modes of action [1]. Microbes continue to serve as a potential storehouse for structurally diverse chemical scaffolds that essentially have been proven for drug discovery [2]. Historically, about 65% of currently used antibiotics are derived from Actinobacteria, especially from *Streptomyces* [3]; thus, making these taxa as the first options for drug discovery screening programs until the end of the 1990s. Due to the massive exploration of Actinobacteria, especially of the genus *Streptomyces*, rediscovery of previously isolated compounds has frequently occurred. This situation led to a shift in the exploration for new secondary metabolites from underexplored genera of the so-called rare Actinobacteria [4]. One of these genera is *Amycolatopsis*, which is known as the producer of vancomycin [5] and rifamycin [6]. In the past few years, several new bioactive compounds have been isolated from this genus, such as the antibacterial agents, macrotermycins A and C that were isolated from a termite-associated *Amycolatopsis* strain [7]. Additional examples are rifamorpholine B and D [8], anticancer dipyrimicin A [9], and 2′-*O*-succinyl-apoptolidin A, as well as 3’-*O*-succinyl-apoptolidin A [10].

During our routine screening program from our rare Actinobacteria collection isolated from Indonesian soil samples, a crude extract of *Amycolatopsis* sp. strain 195334CR exhibited weak activity against *Bacillus subtilis* DSM 10 with a minimum inhibitory concentration (MIC) of 66.7 µg/mL. Analysis of the crude extract by using high-performance liquid chromatography coupled to diode array detection/mass spectrometry (HPLC–DAD/MS) in combination with comparison to entries of the commercial database, Dictionary of Natural Products (DNP, http://dnp.chemnetbase.com (accessed on 17 November 2020)) pointed towards the presence of hitherto unknown metabolites. In this paper, we describe the isolation, structure elucidation, and biological activities of unprecedented cyclic hexapeptides from this strain.

## 2. Results and Discussion

### 2.1. Structure Elucidation of Amycolatomycins

A thorough analysis of the crude extract produced by the rare Actinobacterium *Amycolatopsis* sp strain 195334CR (see Figures S1 and S2 in Supplementary Information SI) by using high performance liquid chromatography–diode array detector–high-resolution mass spectrometry (HPLC–DAD–HRMS) suggested the presence of novel secondary metabolites. Consequently, we conducted large-scale fermentation and purification by using reversed phase (RP) flash chromatography and preparative RP-HPLC led to the isolation of **1** and **2**.

**Figure 1:**
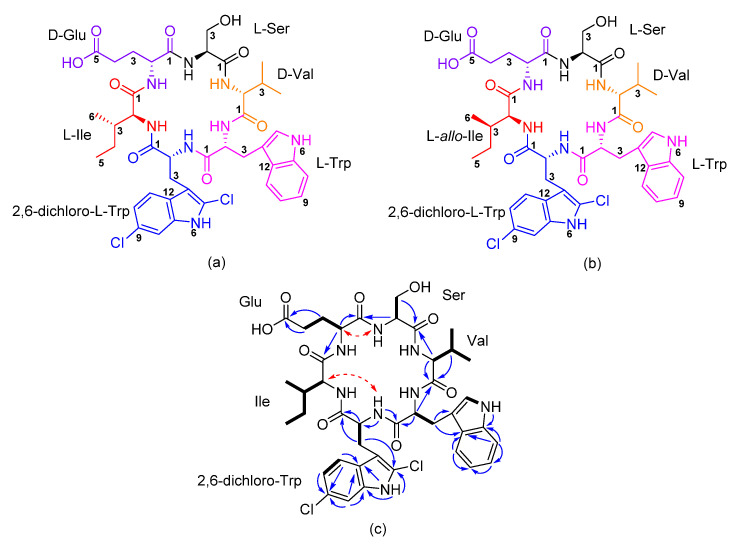
Chemical structure of amycolatomycin A (**a**) and B (**b**); (**c**) selected COSY (bold bond), heteronuclear multiple bond correlation (HMBC) (blue lines), and rotating frame Overhauser effect spectroscopy (ROESY) (red dashed line).

Amycolatomycin A (**1**) was isolated as a white solid. Its monoisotopic mass pattern from high resolution-electrospray ionization mass spectrometry (HR-ESIMS) showed major peaks for [M+H]^+^, [M+2]^+^, and [M+4]^+^ pattern (Figures S3–S5 in SI) gave a hint for the presence of a di-chlorinated compound [11]. Molecular ion cluster at *m/z* 869.3144 [M+H]^+^, and 891.2967 [M+Na]^+^ indicated the molecular formulae of C_41_H_51_Cl_2_N_8_O_9_ (calcd. 869.3151) and C_41_H_50_Cl_2_NaN_8_O_9_ (calcd. 891.2970); thus, accounted for twenty double bond equivalents (DBEs). The ^1^H-nuclear magnetic resonance (NMR) spectrum of **1** in DMSO-*d_6_* displayed signals that were attributable to a peptide, including six α-hydrogens of amino acids between *δ*_H_ 4 and 5 ppm and six amide bonds (-NH) between 7 and 8 ppm. In addition, ^13^C NMR data indicated the presence of seven carbonyls (four overlapped), sixteen olefinic carbon, and four methyl moieties, in combination with 2D NMR data, including correlation spectroscopy (COSY), heteronuclear single quantum coherence (HSQC), and heteronuclear multiple bond correlation (HMBC) allowed us to identify amino acid fragments of valine (Val), serine (Ser), glutamic acid (Glu), isoleucine (Ile), and tryptophan (Trp). All spin systems of the amino acid units were confirmed by total correlation spectroscopy (TOCSY) correlations (see Figures S6–S12 in SI). The peculiar feature of amycolatomycin A (Figure 1) was the occurrence of a 2,6-dichloro-tryptophan (dcT) amino acid unit. The position of the chlorine atom was determined by the HMBC signals of the indole moiety and methylene signal of dcT2 to carbon signal at 125.2 ppm. Furthermore, the HMBC from methine aromatic signals of dcT8, dcT10, and dcT11 to carbon signal at 123.1 ppm (see Table 1) confirmed the positions of chlorine atom. The interpretation of the NMR data for the chlorine atom position is in agreement with the published NMR data of jasplakinolide R_1_ [12].

The sequence of the amino acids was established by analyses of key long-range HMBC, rotating frame Overhauser effect spectroscopy (ROESY) correlations, in combination with mass spectrometry. An Ile was positioned next to Glu by the HMBC correlation from H-2 of Glu to C-1 (*δ*_C_ 170.3 ppm) of Ile. An HMBC signal from H-2 of Ser (*δ*_H_ 4.13 ppm) to C-1 of Glu (*δ*_C_ 170.5 ppm) allowed us to position Ser next to Glu. This connectivity was also supported by the ROESY correlation between the amide proton from Ser (*δ*_H_ 8.14 ppm) with H-2 (*δ*_H_ 4.42 ppm) of Glu. Moreover, the connectivity between Ser and Val was identified based on an HMBC signal at 4.04 ppm (H-2) of Val to Ser carbonyl at 170.3 ppm. Furthermore, an HMBC signal at 4.69 (H-2) of Trp to a carbonyl signal of Val at 170.3 ppm connected Trp and Val. The HMBC correlation from –NH (*δ*_H_ 8.66 ppm) of dcT to C-1 (*δ*_C_ 172.3) of Trp permitted us to connected Trp next to dcT. The connectivity between Ile and 2,6-dichloro-Trp (dcT) was identified based on a ROESY correlation between H-2 (*δ*_H_ 4.09 ppm) of Ile with -NH amide (*δ*_H_ 8.66 ppm) of dcT. In order to confirm our proposed structure, amycolatomycin A was measured in CD_3_OD. Although all of the -NH signals were not observable due to the rapid hydrogen–deuterium exchange with the solvent, the HMBC signals showed all connectivities of the proposed planar structure (NMR signal, table and detailed analysis see Table S1 in SI and Figures S13–S18 in Supporting Information).

Furthermore, the amino acid sequence was confirmed by LC–MS data observed after partial degradation of derivatized d-FDVA-amycolatomycin A. Ring-opening of the cyclic amycolatomycin A into its linear structure was conducted via hydrolysis under the presence of HCl (Figures S19 and S20 in SI). Further hydrolysis of the linear peptide resulted in partial or total degradation of the peptide. The partial hydrolysis of amycolatomycin A showed a molecular mass observed at 1080.44 [M+H]^+^ and gave a hint on the presence of a fragment resulting from the loss of serine; hence, this was correlated to d-FDVA-Val-Trp-dcT-Ile-Glu. Consequently, the linear peptide as a hydrolysis product contained serine as a terminal amino acid, which may have happened between Ser-Glu or Ser-Val. Subsequently, a fragment was detected at *m/z* 860.37 ([M+Na]^+^ for d-FDVA-Val-Trp-dcT along with other fragments equivalent to d-FDVA-Ile (412.18 [M+H]^+^) and d-FDVA-Glu (428.13 [M+H]^+^), respectively. These observations corroborated the ring-opening to be located between the serine and the valine moieties. Finally, additional fragments detected for d-FDVA-Trp-dcT ([M+H]^+^, 739.16) and d-FDVA-Val ([M+H]^+^, 398.16) confirmed the complete amino acid sequence of amycolatomycin A (see Figures S19–S21 in SI).

The absolute configuration of each amino acid was determined with Marfey’s analysis. Degradation of amycolatomycin A (Figure S22 in SI) and the hydrolysate derivatization with *N*-(2,4-dinitro-5-fluorophenyl)- d/l-valinamide (d/l-FDVA, Marfey’s reagent) followed by HPLC–DAD/MS analyses and comparison of Marfey’s derivatized authentic amino acid revealed the presence of l-Ser (d-FDVA *t*_R_ 5.9 min), d-Glu (d-FDVA *t*_R_ 6.1 min), d-Val (d-FDVA *t*_R_ 7.4 min), and l-Ile (d-FDVA *t*_R_ 9.7 min) (HPLC–DAD/MS retention time of authentic amino acid-derived d or l-FDVA see Figures S23–S25 and Tables S2–S3 in SI). Since there are four stereoisomer of Ile (l or l-*allo*-Ile and d or d-*allo*-Ile) and under our standard HPLC method employing C_18_ column those stereoisomers were not separated, a modified C_3_ Marfey’s analysis (C_3_ refer to an HPLC column) introduced by Vijayasarathi et al. [13] was used.

Specific optimized conditions to resolve the l-Ile stereoisomer from l-*allo*-Ile were achieved by using a C_4_ column and confirmed the presence of l-Ile (C_4_ Marfey’s analysis, d-FDVA *t*_R_ 21.40 min) in amycolatomycin A (see Figures S26 and S27 in SI). The determination of the absolute configuration of tryptophan-containing units using several direct methods was unsuccessful due to the degradation of tryptophan, even when phenol was used as a protecting agent [14] (data not shown). Finally, the determination of the absolute configuration of tryptophan was achieved by converting tryptophan into aspartic acid catalyzed by RuCl_3_-NaIO_4_ [15,16] followed by hydrolysis and Marfey’s derivatization. According to our LC–MS data (see Figures S28 and S29 in SI), we observed l-aspartic acid (d-FDVA *t*_R_ 6.1 min and l-FDVA *t*_R_ 5.8 min), which indicated the presence of l-tryptophan in amycolatomycin A. Since there are two tryptophan (one in di-chlorinated form) in amycolatomycin A, and we observed only l-aspartic acid, we conclude that the tryptophan and 2,6-dichloro-tryptophan have the same l-absolute configuration.

Amycolatomycin B (**2**) was also isolated as a white solid. The UV, monoisotopic mass pattern, hydrolysis, and NMR data (Figures S30–S39 in SI) of **2** were very similar to amycolatomycin A, indicating that **2** represents a stereoisomer of **1**. Interpretation of 1D and 2D NMR data resulted in a planar structure identical to amycolatomycin A (**1**). The ^1^H and ^13^C NMR data of **2** showed that the α-CH and CH for Ile at C-2 position were more deshielded compared to **1** for the proton chemical shift and more shielding for the carbon chemical shift (*δ*_H_/*δ*_C_: 4.50/57.2 ppm in **2**, instead of 4.09/57.8 ppm in **1**), indicating that **2** bearing *allo-* isoleucine [17]. The application of Marfey’s analysis for amycolatomycin B in an analogous manner as described above revealed l-Ser (d-FDVA *t*_R_ 5.9 min), d-Glu (d-FDVA *t*_R_ 6.1 min), and d-Val (d-FDVA *t*_R_ 7.4 min). Further analysis of Marfey’s method on C_4_ column (see Figures S40 and S41 in SI) confirmed the presence of l-*allo*-isoleucine (d-FDVA *t*_R_ 21.13 min) in amycolatomycin B.

### 2.2. In Silico Analysis of the Amycolatomycin Biosynthetic Gene Clusters (BGCsBCGs)

**Figure 2:**
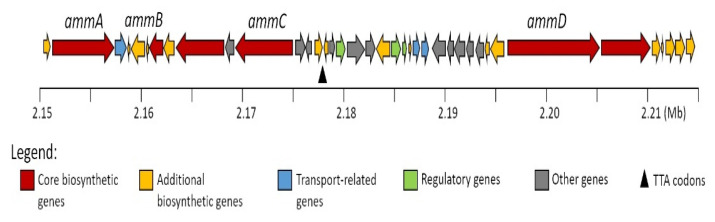
Putative biosynthetic gene clusters (BCGs) Encoding for Amycolatomycins.

The genomic analysis of *Amycolatopsis* sp. strain 195334CR yielded eight contigs with a total length of 9,926,854 bp. Examination of contigs using antiSMASH [18] revealed 33 regions hypothetically encoding secondary metabolite gene clusters. Amycolatomycins is a cyclic non-ribosomal peptide consisting of six amino acids with chlorination at one of Trp residues in its structure. Analysis of the peptide backbone of the amycolatomycins suggested that the non-ribosomal peptide synthetase (NRPS) system should contain six modules, considering a canonical sequential assembly of the amino acid residues [19,20]. According to the antiSMASH analysis result, four open reading frames (orfs) were identified in one region as putatively structural genes for amycolatomycins biosynthesis designated as *ammA*–*ammD* (Figure 2, see Table S4 in SI for nucleotide data of each biosynthetic gene cluster (BGC)).

A tryptophan halogenase encodes by *ammB*, which shows high homology (77% identity, 88% similarity) to FADH_2_-dependent halogenase from *Micromonospora* sp. GMKU326. This halogenase in *ammB* is most likely responsible for generating the 2,6-dichloro-tryptophan.

**Figure 3:**
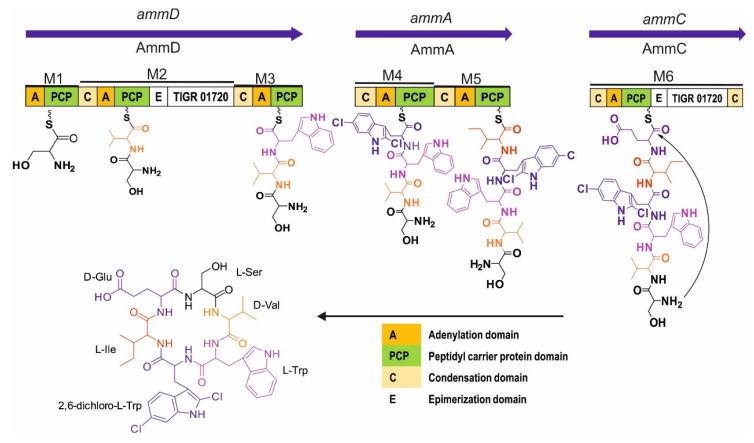
Proposed Biosynthesis of Amycolatomycin A.

On the other hand, *ammA*, *ammC*, and *ammD* encode multimodular NRPS consisting of six modules in total. The AmmD protein comprises modules 1, 2, and 3 and has relatively low homology with NRPS from *Streptomyces* sp. KCB13F003 (42% identity, 54 % similarity). The adenylation domain of module 1, 2, and 3 is predicted to incorporate serine (Ser), valine (Val), and tryptophan (Trp), respectively (Table 2). Module 2 contains an epimerase domain that may be responsible for modifying L-Val to D-Val. Module 4 and 5 are located in AmmA protein with 37% identity and 55 % similarity to PuwF-G, NRPS for biosynthesis puwainaphycin from *Cylindrospermum moravicum* CCALA 993. The prediction for the adenylation domain of modules 4 and 5 is 2,6-dichloro-Trp (2,6-dichloro-Tryptophan) and isoleucine (Ile), respectively. Module 6, which is in AmmC, comprises the adenylation domain, which are presumed for glutamic acid (Glu), and the epimerase domain that is putatively having a role in D-Glu synthesis. AmmC has 40% identity and 52 % similarity to Atr21, NRPS for biosynthesis atratumycin from *Streptomyces atratus*. Moreover, AmmC has a terminal condensation domain (CT), which is likely important for cyclization process of amycolatomycins as reported similarly with the role of CT for biosynthesis of cyclosporine A, aureobasidin A, apicidin, ferrichrome A, sansalvamide, and destruxin [21,22]. Based on the prediction of the function AmmA, AmmC, and AmmD, the proposed biosynthesis of amycolatomycin is depicted in Figure 3.

### 2.3. Biological Activity of Amycolatomycin A

Due to the insufficient material of compound **2**, only amycolatomycin A (**1**) was evaluated for antimicrobial activities against several pathogens, as well as its cytotoxicity against several cancer cell lines. Amycolatomycin A exhibited weak antimicrobial activity against *Bacillus subtilis* DSM 10 with a MIC value at 33.4 µg/mL and has no other activities in our standard antimicrobial assay protocol [23].

Tryptophan is a biosynthetic precursor for numerous complex microbial natural products, which many of these molecules are promising scaffolds for drug discovery and development [24]. Natural products containing mono-chlorinated tryptophan are common in nature, with an exception for chlorination at the C-2 position of tryptophan. Of these molecules is a peptide, krysinomycin, isolated from the *Streptomyces fradiae* strain MA7310 [25], inducamides, an alkaloid isolated from a mutant strain of *Streptomyces* [26], and keramamide A, a peptide isolated from marine sponge *Theonella sp*. [27], whereas the only examples of chlorinated tryptophan at the C-2 position are the chondramides, cyclodepsipeptides isolated from *Chondromyces crocatus* (Myxobacteria) [28].

According to the literature survey, there is no reported secondary metabolites bearing di-chlorinated tryptophan amino acid in the public (https://pubchem.ncbi.nlm.nih.gov) (accessed on 17 November 2020), commercial database (DNP (http://dnp.chemnetbase.com) (accessed on 17 November 2020), or SciFinder (https://scifinder.cas.org) (accessed on 17 November 2020); thus, making amycolatomycins the first reported example of secondary metabolite containing a di-chlorinated tryptophan amino acid unit.

## 3. Materials and Methods

### 3.1. General Experimental Procedure

Analytical reversed phase (RP) HPLC and fractionation were performed on an Agilent (Agilent Technologies, Santa Clara, CA, USA) 1100 HPLC system. HPLC conditions: XBridge C18 column 100 × 2.1 mm (Waters, Milford, MA, USA), 3.5 μm, solvent A (5% acetonitrile (ACN) in water, 5 mmol ammonium acetate (NH_4_OAc), 0.04 mL/L CH_3_COOH); solvent B (95% ACN, 5 mmol (NH_4_OAc,) 0.04 mL/L CH_3_COOH); gradient system: from 10% B to 100% B in 30 min and maintaining at 100% for 10 min, followed by post-run from 100% to the initial condition for 10 min; flow rate 0.3 mL/min; 40 °C; fractionation was performed in 96-well microtiter plates and collected every 30 s. High-resolution electrospray ionization mass spectrometry (HR-ESIMS) data were recorded on a MaXis ESI–TOF (Time of Flight) mass spectrometer (Bruker Daltonics, Bremen, Germany) equipped with an Agilent 1260 series HPLC-UV system (column C18 Acquity UPLC BEH (Ultra Performance Liquid Chromatography Ethylene Bridged Hybrid)(Waters), solvent A: H_2_O + 0.1% formic acid; solvent B: ACN + 0.1% formic acid, gradient: 5% B for 0.5 min, increasing 19.5 min to 100% B, holding 5 min at 100% B; flow rate 0.6 mL/min, 40 °C; DAD–UV detection at 200–600 nm). Molecular formulas were calculated using the Smart Formula algorithm (Bruker Daltonics). HPLC–DAD/MS analysis was performed using an amaZon speed ETD (Electron Transfer Dissociation) ion trap mass spectrometer (Bruker Daltonics) in positive and negative ion modes. HPLC system (column C18 Acquity UPLC BEH (Waters), solvent A: H_2_O + 0.1% formic acid; solvent B: acetonitrile (ACN) + 0.1% formic acid, gradient: 5% B for 0.5 min, increasing to 100% B in 20 min, maintaining isocratic conditions at 100% B for 10 min, flow rate 0.6 mL/min, UV/Vis detection 200–600 nm). Preparative HPLC was carried out on an Agilent (Santa Clara, CA, USA) 1100 series system (ChemStation Rev. B.04.03 SP1 software), comprising a binary pump system, a diode-array detector and a 180-fraction collector. NMR spectra were recorded on a Bruker 700 MHz Avance III spectrometer with a 5 mm TCI cryoprobe (^1^H: 700 MHz, ^13^C: 175 MHz), locked to the deuterium signal of the solvent. Chemical shifts are given in parts per million (ppm) and coupling constants in Hertz (Hz). UV spectra were measured on a Shimadzu (Kyoto, Japan) UV/Vis 2450 spectrophotometer, using methanol (Uvasol, Merck, Darmstadt, Germany). Optical rotations were measured using an Anton Paar MCP-150 Polarimeter (Graz, Austria) with 100 mm path length and sodium D line at 589 nm.

### 3.2. Origin of the Strain

Strain 195334CR was obtained from a soil sample collected from Cultural Park Bali, Indonesia, according to a previously described method [29].

### 3.3. Analysis of 16S rRNA Sequences

Genomic DNA extraction, amplification of 16S rRNA gene and the purification of the polymerase chain reaction (PCR) product were performed using the method described by Mohr et al. [30]. DNA sequencing was carried out by using a 96-capillary-system from Applied Biosystems (ABI), 3730xl DNA Analyzer employing five primers for sequencing: F27 (5′-AGAGTTTGATCMTGGCTCAG-3′), R518 (5′-CGTATTACCGCGGCTGCTGG-3′), F1100 (5′-YAACGAGCGCAACCC-3′), R1100 (5′-GGGTTGCGCTCGTTG-3′), and R1492 (5′-TACGGYTACCTTGTTACGACTT-3′). BioEdit software (version 7.0.5.3) was used for editing and generating the contig of the 16S rRNA gene sequence [31]. The 16S rRNA gene sequence of *Amycolatopsis* sp. 195334CR was deposited in GenBank with the accession number MW194079.

Investigation of the closely related type strains based on 16S rRNA gene sequence similarities was conducted using the EzTaxon-e server (https://www.ezbiocloud.net/taxonomy [32]; accessed on 4 January 2021). The 16S rRNA gene sequences were aligned using the MUSCLE algorithm [33] from the MEGA X software package version 10.0.5 for windows (MEGA X, Penn State University, Pennsylvania, USA) [34]. The phylogenetic tree was inferred from the maximum likelihood [35] algorithm and the topology of the tree was calculated by bootstrap analysis [36] based on 100 replicates.

### 3.4. Scale-Up Production, Extraction, and Isolation of Compounds

Seed cultures were prepared by inoculating three plugs of seven-day well-grown culture in agar medium (containing malt extract 10 g, glucose 4 g, yeast extract 4 g, and 40 g agar in 1L tap water, and pH adjusted to 6.3 before sterilization) to 250 mL flask filled with 100 mL of liquid medium containing the same compositions as the agar medium, and incubated for nine days. After nine days, 500 µL of the seed cultures were used to inoculate a medium consisted of soluble starch (15 g/L), yeast extract (4 g/L), K_2_HPO_4_ (1 g/L), CaCl_2_ (300 mg/L), and MgSO_4_.7H_2_O (0.5 g/L) in 1 L of tap water, pH adjusted to 7 with 20% HCl before sterilization, and incubated at 37 °C on a rotary shaker (120 rpm). In total, 18 L of fermentation was conducted in four batches. On day seven, the fermentation was terminated. Mycelial and supernatant were separated by centrifugation (9000 rpm, 10 min). The mycelial cake was extracted with 500 mL ethyl acetate (1 time) and methanol (500 mL, 3 times) under an ultrasonic bath. According to analytical HPLC, the ethyl acetate and methanol layer were combined due to their similar profile and dried under vacuum to provide 372 mg of crude mycelial extract. The crude mycelial extract was redissolved in 20% MeOH/H_2_O (700 mL) and partitioned with an equal amount of n-heptane (3 times), resulting in 180 mg of MeOH soluble fraction after dried under vacuum. The MeOH soluble fraction was redissolved in methanol (5 mL), sonicate for 2 min, and the resulted suspension was then centrifuged at 9000 rpm for 5 min, followed by drying under nitrogen stream until half of the volume was reached. The soluble fraction was subsequently subjected to flash chromatography (Grace Reveleris^®^, Maryland, USA) [FlashPure-C18 cartridge (Büchi, Flawil, Switzerland), 12 g, line 1 (A): H_2_O, line 2 (B): acetonitrile (ACN), gradient: 10% B for 1 min, increasing to 35% B in 5 min, followed by slowly increasing to 77% B in 25 min and then to 100% B in 10 min and finally hold at 100% B for 10 min]. Three fractions were collected according to the peaks in the UV chromatogram. Fraction 2 (13 mg) was further purified by preparative reversed phase (RP) HPLC [Phenyl-hexyl, 5 µm column, 250 × 21.2 mm (Macherey-Nagel, Düren, Germany), solvent A: water, solvent B: acetonitrile, flow rate 20 mL/min and UV detection at 210, 230, and 280 nm, gradient: 43% B isocratic for 2 min, from 43% B to 47% B in 3 min and 47% B isocratic for 43 min, then increasing to 100% B in 5 min and held at 100% B in 7 min] (see Figure S42 in SI for isolation process chromatogram) to deliver compound **1** (3.1 mg, *t*_R_ = 14.0 min) and compound **2** (1.4 mg, *t*_R_ = 6.1 min).

Amycolatomycin A (**1**): white solid; UV λ_max_ MeOH (log *ε*) 226 (4.83) nm, 287 (4.03) nm; ${\left[\alpha \right]}_{D}^{20}$ +35 (*c* 0.1, MeOH); NMR data (^1^H: 700 MHz, ^13^C 176 MHz, DMSO-*d*_6_) see Table 1; HR-ESIMS: [M+H]^+^ calcd for C_41_H_51_Cl_2_N_8_O_9_, *m/z* 869.3151, found 869.3144 [M+H]^+^ calcd for C_41_H_51_Cl_2_N_8_O_9_, [M+Na]^+^ calcd for C_41_H_50_Cl_2_NaN_8_O_9_, *m/z* 891.2970, found 891.2967 [M+Na]^+^ calcd for C_41_H_50_Cl_2_NaN_8_O_9_, *t*_R_ = 10.29 min.

Amycolatomycin B (**2**): white solid; UV λ_max_ MeOH (log *ε*) 227 (3.9) nm, 288 (4.7) nm; ${\left[\alpha \right]}_{D}^{20}$ +29 (*c* 0.1, MeOH); HR-ESIMS: [M+H]^+^ calcd for C_41_H_51_Cl_2_N_8_O_9_, *m/z* 869.3152, found 869.3141 [M+H]^+^ calcd for C_41_H_51_Cl_2_N_8_O_9_, [M+Na]^+^ calcd for C_41_H_50_Cl_2_NaN_8_O_9_, *m/z* 891.2965, found 891.2960 [M+Na]^+^ calcd for C_41_H_50_Cl_2_NaN_8_O_9_, *t*_R_ = 10.28 min. ^1^H-NMR (700 MHz, DMSO-*d*_6_) δ ppm 8.67 (m, -NH-Val2), 3.83 (m, H-Val2), 2.25 (td, *J* = 6.9, 4.4 Hz, H-Val3), 0.97 (t, t, *J* = 7.1 Hz, H_3_-Val4), 0.96 (t, *J* = 7.1 Hz, H_3_-Val5), 4.20 (td, *J* = 8.1, 4.8 Hz, H-Ser2), 3.46 (br d, *J* = 8.4 Hz, H-Ser3``), 3.62 (m, H-Ser3`), 4.43 (br s, H-Glu2), 2.22 (m, H-Glu3`), 1.84 (m, H-Glu3``), 2.20 (m, 2H-Glu4), 4.50 (br s, H-Ile2), 1.78 (br s, H-Ile3), 1.34 (br s, H-Ile4`), 1.01 (m, H-Ile4``), 0.77 (t, *J* = 7.4 Hz, H_3_-Ile5), 0.73 (br d, *J* = 4.5 Hz, H_3_-Ile6), 4.45 (m, H-dcT2), 3.30 (m, H-dcT3`), 2.94 (m, H-dcT3``), 10.95 (d, *J* = 1.7 Hz, NH-dcT6), 7.37 (d, *J* = 1.9 Hz, H-dcT8), 6.98 (dd, *J* = 8.5, 1.8 Hz, H-dcT10), 7.56 (d, *J* = 8.4 Hz, H-dcT11), 4.40 (m, H-Trp2), 2.97 (m, H-Trp3`), 2.85 (br s, H-Trp3``), 6.71 (br s, H-Trp5), 10.90 (br s, NH-Trp6), 7.29 (s, H-Trp8), 7.02 (dd, *J* = 8.6, 1.9 Hz, H-Trp9), 7.51 (d, *J* = 1.7 Hz, H-Trp11). ^13^C NMR (176 MHz, DMSO-*d*_6_) δ ppm 60.7 (CH-Val2), 28.7 (CH-Val3), 17.2 (CH_3_-Val4), 19.1 (CH_3_-Val5), 55.5 (CH-Ser2), 61.7 (CH_2_-Ser3), 51.8 (CH-Glu2), 26.4 (CH_2_-Glu3), 33.6 (CH_2_-Glu4), 177.1 (C-Glu5), 57.2 (CH-Ile2), 36.9 (CH-Ile3), 24.0 (CH_2_-Ile4), 11.5 (CH_3_-Ile5), 15.0 (CH_3_-Ile6), 53.4 (CH-dcT2), 26.8 (CH_2_-dcT3), 111.2 (C-dcT4), 125.5 (C-dcT5), 136.4 (C-dcT7), 110.9(CH-dcT8), 125.3 (C-dcT9), 118.5(CH-dcT10), 119.6 (CH-dcT11), 126.2 (C-dcT12), 55.7 (CH-Trp2), 27.9 (CH_2_-Trp3), 110.1 (C-Trp4), 124.9 (CH-Trp5), 134.4 (C-Trp7), 112.7 (CH-Trp8), 120.7 (CH-Trp9), 123.0 (CH-Trp10), 117.3 (CH-Trp11), 128.3 (C-Trp12), 169.4 (C-Ser1), 170.0 (C-Trp1&Glu1), 171.0 (CdcT1&Val1), and 171.6 (C-Ile1).

### 3.5. Ring-Opening and Partial Hydrolysis of Amycolatomycin A

Ring-opening to convert amycolatomycin A to its linear peptide and the partial hydrolysis was conducted according to Vijayasarathy et al. [13], with slight modification. In detail, amycolatomycin A (100 µg) was added with 100 µL of 2M HCl and heated at 70 °C for 3 h. After 3 h, 50 µL aliquot was taken and subjected to LCMS analysis. The remaining solution was heated at 100 °C for the next 3 h. After 3 h, the hydrolysate was dried under N_2_ and then treated with 1m of NaHCO_3_ (20 µL) and 40 µL of D-FDVA (1% solution in acetone) and heated at 40 °C for 1 h. After 1 h, the reaction mixture was diluted with 40 µL ACN and subjected to LC–MS analysis.

### 3.6. Determination of Absolute Amino Acid Stereochemistry

The determination of absolute stereochemistry of the amino acid units was conducted according to Pérez-Bonilla et al. [37]. In detail, amycolatomycins (100 µg) was subjected to acid hydrolysis with 100 µL of 6 N n HCl at 110 °C for 10 h. The acid hydrolysate was evaporated under N_2_ gas stream until dried, redissolved in 100 µL of distilled H_2_O, and divided into two individual vials. The reaction vial was completely dried under N_2_ gas stream and dissolved in 20 µL of 1 M NaHCO_3_. In one vial, 40 µL of 1% L-FDVA in acetone was added and another vial was added with D-FDVA, at the same time, authentic amino acids (D, L, or D/L mixture) were prepared in the same manner of the hydrolysis product and incubated at 40 °C for an hour. After 1 h, the reaction mixtures were diluted with 40 µL of ACN and subjected to LCMS measurement using an amaZon speed ETD ion trap mass spectrometer (column and conditions see General Experimental Procedure).

### 3.7. Determination of Tryptophan Absolute Amino Acid Stereochemistry

The tryptophan moiety in amycolatomycin A was converted into aspartic acid according to Chan et al. [15] and Ranganathan et al. [16]. Briefly, in a reaction vial, 100 µg of amycolatomycin amylocatomycin A dissolved in 200 µL of acetonitrile was added to a mixture of CHCl_3_-H_2_O (1:2; 300 µL), RuCl_3_·H_2_O (in catalytic amount), and NaIO_4_ (18 Equiv). The reaction vial was then sealed and stirred for 60 h, filtrated and dried under vacuum. The remaining residue was treated in the same manner as previously described in Section 3.4.

### 3.8. Determination of Isoleucine Stereoisomer Absolute Stereochemistry with C_4_ HPLC–DAD/MS Marfey’s Analysis

Marfey’s derivatives of authentic amino acid d-, l-, d-*allo*, and l-*allo* isoleucine were prepared and analyzed on a Dionex Ultimate 3000 HPLC system (Thermo-Fischer Scientific, Waltham, MA, USA) equipped with DAD/UV and an ion trap MS (amazon speed ETD, Bruker Daltonics) detector measured in positive and negative mode simultaneously. The separation was performed with a ternary mobile phase system comprises of H_2_O (A) and MeOH (B) added with 5% ACN as ternary mobile phase and 1 % (of ternary solvent) formic acid as a modifier. An Orbit 100 (250 × 4 mm, 5µm) C_4_ column (MZ analysentechnik GmbH, Mainz, Germany) was used with flowrate at 0.8 mL/min, the oven temperature was set to 50 °C, and wavelength at 340 nm was used for detection. The gradient system starts with 55% B to 80% B over 50 min, then increases to 100% B in 10 min, and finally holds at 100% B for 10 min before returning to the initial gradient.

### 3.9. Genomic DNA Isolation, Sequencing, and Bioinformatic Analysis

Genomic DNA of *Amycolatopsis* sp. 195334CR was extracted by using NucleoSpin Microbial DNA Mini Kit (Macherey-Nagel) following the manufacturer’s instruction. The genome of the bacterial strain was sequenced and de novo assembled using Illumina next-generation sequencing technology with MiSeq 600 cycle v3 and Unicycler [38], respectively. The assembled draft genome of *Amycolatopsis* sp. 195334CR was deposited at DDBJ/ENA/GenBank under the accession JAFJMJ000000000. Investigation of biosynthetic gene clusters (BGCsBCGs) was performed using web-based antiSMASH version 5.2.0 [18,39] (https://antismash.secondarymetabolites.org/) (accessed on 17 December 2020), and further analysis of the translated sequences was conducted by using the BLASTP (Basic Local Alignment Search Tool) algorithm [40] (https://blast.ncbi.nlm.nih.gov/) (accessed on 30 December 2020).

### 3.10. Antimicrobial and Cytotoxic Activities

Antimicrobial and cytotoxic activities of amycolatomycin A were determined by our established protocol, according to Surup et al. [23] and Becker et al. [41].

## 4. Conclusions

Our current study demonstrated that rare Actinobacteria are still a valuable source for novel bioactive metabolites, since two previously undescribed cyclo-hexapeptide contain a dichlorinated tryptophan moiety as an unique feature have been isolated from the underexplored genera *Amycolatopsis*, isolated from an Indonesian soil sample. The innovative approach, by combining the classical method of the isolating producer strain and its secondary metabolites with the whole genome sequencing analysis, which can only be possible due to the rapid development of the bioinformatics tools and the lower cost of the next-generation sequencing, are proven to have a crucial role in the field of natural product chemistry

## Figures and Tables

**Table 1:** ^1^H and ^13^C NMR spectroscopic data of compound **1** in DMSO-*d_6_* (^1^H 700.4 MHz; ^13^C 176.1 MHz).

Unit	Pos	*δ*_H_, Mult (*J* in Hz)	*δ*_C_	Unit	Pos	*δ*_H_, Mult (*J* in Hz)	*δ*_C_
dcT	1	−	170.8, C	Val	1	−	170.3, C
	2	4.28, m	54.4, CH		2	4.04, d (br) (2.0)	58.3, CH
	2NH	8.66, d (8.4)	−		2NH	8.40, d (7.0)	
	3	3.16, m; 2.92, dd (14.0, 10.4)	27.0, CH_2_		3	2.27, m	29.0, CH
	4	−	110.5, C		4	0.85, d (6.8)^ov^	19.3, CH^3^
	5-Cl	−	125.2, C		5	0.84, d (6.8)^ov^	16.9, CH^3^
	6NH	11.00, s	−	Ser	1	−	170.3, C
	7	−	134.4, C		2	4.13, q (6.7 × 3)	56.1, CH
	8	7.57, d (2.0)	112.8, CH		2NH	8.14, s	−
	9-Cl	−	123.1, C		3	3.30^a^	60.5, CH_2_
	10	7.04, dd (8.5, 2.0)	120.7, CH	Glu	1	−	170.5, C
	11	7.33, d (8.5, 2.0)	117.4, CH		2	4.42, q (6.7 × 3)	51.1, CH
	12	−	128.3, C		2NH	n.o	−
Trp	1	−	170.3, C		3	1.87, m	28.2, CH_2_
	2	4.69, d (br) (6.0)	53.5, CH		4	2.19, t (8.0)	30.2, CH_2_
	2NH	n.o			5	−	174.3, C
	3	3.11, m; 2.97, dd (14.0, 5.6)	28.3, CH_2_	Ile	1	−	170.3, C
	4	−	110.1, C		2	4.09, s (br)	57.8, CH
	5	7.13, s	124.7, CH		2NH	n.o	−
	6NH	10.97, d (2.0)	−		3	1.55, s (br)	35.1, CH
	7	−	136.3, C		4	1.22, m; 0.93, m	24.8, CH_2_
	8	7.35, d (2.0)	110.7, CH		5	0.75, t (7.4)	10.8, CH_3_
	9	6.99, dd (8.5, 2.0)	118.5, CH		6	0.51, d (6.2)	14.6, CH_3_
	10	n.o	125.5, C				
	11	7.56, s	120.0, CH				
	12	−	126.4, C				

n.o: not observed; a) overlapped with H_2_O; ov: overlapped signal.

**Table 2:** List of Biosynthetic Genes and Proteins Putatively Associated with Amycolatomycins.

Gene (Nucleotide)	Protein (Amino Acid)	Proposed Function	Percent Identity and Similarity to Protein /Origin
*ammA* (6279)	AmmA (2092)	NRPS: C_4_ A_4_ (2,6-dichloro-Trp) PCP_4_ C_5_ A_5_ (Ile) PCP_5_	37%, 55%: AXN93581.1, PuwF-G [*Cylindrospermum moravicum* CCALA 993]
*ammB* (1290)	AmmB (429)	Halogenase	77%, 88%: BAQ25509.1, FADH_2_-dependent_halogenase/*Micromonospora* sp. GMKU326
*ammC* (5865)	AmmC (1954)	NRPS: C_6_ A_6_ (Glu) PCP_6_ E_6_ TIGR01720 C_T_	40%, 52%: QBG38782.1, Atr217/*Streptomyces atratus*
*ammD* (9192)	AmmD (3063)	NRPS: A_1_(Ser) PCP_1_ C_2_ A_2_ (Val) PCP_2_ E_2_ TIGR01720 C_3_ A_3_ (Trp) PCP_3_	42%, 54%: ATU31794.1, NRPS/*Streptomyces* sp. KCB13F003

## Data Availability

The GenBank accession number for the 16S rRNA gene sequence of *Amycolatopsis* sp. 195334CR is MW194079. The GenBank accession number of the assembled draft genome of *Amycolatopsis* sp. 195334CR is JAFJMJ000000000.

## Supplementary Materials

The following are available online at https://www.mdpi.com/2079-6382/10/3/261/s1, Figure S1: Amycolatopsis sp. 195334CR on GYM agar plate, Figure S2: The phylogenetic tree based on the nearly complete 16S rRNA gene sequence, Figure S3: HPLC–DAD/MS chromatogram of amycolatomycin A, Figure S4: HR-ESIMS chromatogram of amycolatomycin A, Figure S5: UV-vis spectrum of amycolatomycin A in MeOH, Figure S6–S12: 1D and 2D NMR spectrum of amycolatomycin A in DMSO-d6, Figure S13–S18: 1D and 2D NMR spectrum of amycolatomycin A in CD3OD, Figure S19: Reaction scheme of hydrolysis amycolatomycin A under acidic condition, Figure S20: HPLC-DAD/MS chromatogram of hydrolysis amycolatomycin A under an acidic condition Figure S21: HPLC-DAD/MS of partial degradation of linear amycolatomycin under an acidic condition at 100 °C, Figure S22: Partial degradation scheme of linear amycolatomycin A under acidic condition at 100 °C, Figure S23: General reaction of Marfey’s reagent and amino acid, Figure S24: HPLC-DAD/MS chromatogram of L/D or DL authentic amino acid derived D-FDVA, Figure S25: HPLC-DAD/MS chromatogram of L/D or DL authentic amino acid derived L-FDVA, Figure S26: HPLC-DAD/MS of Amycolatomycin A catalyzed by RuCl3·H2O-NaIO4 followed by hydrolysis and derivatization with L-FDVA on C18 column, Figure S27: HPLC-DAD/MS of Marfey’s analysis on C4 column of an authentic amino acid L and L-allo-Ile: co-injection of D-FDVA-L-Ile-L-allo-Ile (a), D-FDVA-L-Ile (b), and D-FDVA-L-allo-Ile, Figure S28: HPLC-DAD/MS of Marfey’s analysis of amycolatomycin A derived D-FDVA in C18 column, Figure S29: HPLC-DAD/MS of Amycolatomycin A catalyzed by RuCl3·H2O-NaIO4 followed by hydrolysis and derivatization with D-FDVA on C18 column, Figure S30: HPLC-DAD/MS chromatogram of amycolatomycin B, Figure S31: HR-ESIMS chromatogram of amycolatomycin B, Figure S32: UV/vis spectrum of amycolatomycin B in MeOH, Figure S33-S39: 1D and 2D NMR spectrum of amycolatomycin A in DMSO-d6, Figure S40: HPLC-DAD/MS of Marfey’s analysis on C4 column of amycolatomycin A derived D-FDVA, Figure S41: HPLC-DAD/MS of Marfey’s analysis on C4 column of amycolatomycin B derived D-FDVA, Figure S42: Isolation process chromatogram of GF2 fraction in preparative HPLC. Table S1: 1H NMR and 13C NMR of amycolatomycin A in CD3OD, Table S2: Retention time of L or D authentic amino acid derived D-FDVA, Table S3: Retention time of L or D authentic amino acid derived L-FDVA. Table S4. List of amycolatomycin biosynthetic genes and its nucleotide sequence (*ammA*-*ammD*).

## References

[1] Stadler M., Dersch P. (2017). How to overcome the antibiotic crisis – Facts, challenges, technologies & future perspectives. Curr Top. Microbiol Immunol..

[2] Genilloud O. (2018). Mining actinomycetes for novel antibiotics in the omics era: Are we ready to exploit this new paradigm?. Antibiotics.

[3] Tiwari K., Gupta R.K. (2012). Rare actinomycetes: A potential storehouse for novel antibiotics. Crit. Rev. Biotechnol..

[4] Ding T., Yang L.-J., Zhang W.-D., Shen Y.-H. (2019). The secondary metabolites of rare actinomycetes: Chemistry and bioactivity. RSC Adv..

[5] Xu L., Huang H., Wei W., Zhong Y., Tang B., Yuan H., Zhu L., Huang W., Ge M., Yang S. (2014). Complete genome sequence and comparative genomic analyses of the vancomycin-producing *Amycolatopsis orientalis*. BMC Genom..

[6] Zhao W., Zhong Y., Yuan H., Wang J., Zheng H., Wang Y., Cen X., Xu F., Bai J., Han X. (2010). Complete genome sequence of the rifamycin SV-producing *Amycolatopsis mediterranei* U32 revealed its genetic characteristics in phylogeny and metabolism. Cell Res..

[7] Beemelmanns C., Ramadhar T.R., Kim K.H., Klassen J.L., Cao S., Wyche T.P., Hou Y., Poulsen M., Bugni T.S., Currie C.R. (2017). Macrotermycins A-D, glycosylated macrolactams from a termite-associated *Amycolatopsis* sp. M39. Org. Lett..

[8] Xiao Y.S., Zhang B., Zhang M., Guo Z.K., Deng X.Z., Shi J., Li W., Jiao R.H., Tan R.X., Ge H.M. (2017). Rifamorpholines A-E, potential antibiotics from locust-associated actinobacteria: *Amycolatopsis* sp. Hca4. Org. Biomol. Chem..

[9] Izuta S., Kosaka S., Kawai M., Miyano R., Matsuo H., Matsumoto A., Nonaka K., Takahashi Y., Omura S., Nakashima T. (2018). Dipyrimicin A and B, microbial compounds isolated from *Amycolatopsis* sp. K16-0194. J. Antibiot..

[10] Sheng Y., Fotso S., Serrill J.D., Shahab S., Santosa D.A., Ishmael J.E., Proteau P.J., Zabriskie T.M., Mahmud T. (2015). Succinylated apoptolidins from *Amycolatopsis* sp. ICBB 8242. Org. Lett..

[11] Palaniappan K.K., Pitcher A.A., Smart B.P., Spiciarich D.R., Iavarone A.T., Bertozzi C.R. (2011). Isotopic signature transfer and mass pattern prediction (IsoStamp): An enabling technique for chemically-directed proteomics. ACS Chem. Biol..

[12] Watts K.R., Morinaka B.I., Amagata T., Robinson S.J., Tenney K., Bray W.M., Gassner N.C., Lokey R.S., Media J., Valeriote F.A. (2011). Biostructural features of additional jasplakinolide (jaspamide) analogues. J. Nat. Prod..

[13] Vijayasarathy S., Prasad P., Fremlin L.J., Ratnayake R., Salim A.A., Khalil Z., Capon R.J. (2016). C3 and 2D C3 Marfey’s methods for amino acid analysis in natural products. J. Nat. Prod..

[14] Muramoto K., Kamiya H. (1990). Recovery of tryptophan in peptides and proteins by high-temperature and short-term acid hydrolysis in the presence of phenol. Anal. Biochem..

[15] Chan C.-O., Crich D., Natarajan S. (1992). Enantiospecific Synthesis of Amino Acids: Preparation of *(R)*- and *(S)*-α- methylaspartic acid from *(S)*-tryptophan. Tetrahedron Lett..

[16] Ranganathan S., Ranganathan D., Bhattacharyya D. (1987). The Transformation of Tryptophan to Aspartic Acid in Peptides. J. Chem. Soc. Chem. Commun..

[17] Anderson Z.J., Hobson C., Needley R., Song L., Perryman M.S., Kerby P., Fox D.J. (2017). NMR-based assignment of isoleucine: Vs. *allo* -isoleucine stereochemistry. Org. Biomol. Chem..

[18] Blin K., Shaw S., Steinke K., Villebro R., Ziemert N., Lee S.Y., Medema M.H., Weber T. (2019). AntiSMASH 5.0: Updates to the secondary metabolite genome mining pipeline. Nucleic Acids Res..

[19] Challis G.L., Naismith J.H. (2004). Structural aspects of non-ribosomal peptide biosynthesis. Curr. Opin. Struct. Biol..

[20] Amoutzias G.D., Van de Peer Y., Mossialos D. (2008). Evolution and taxonomic distribution of non-ribosomal peptide and polyketide synthases. Future Microbiol..

[21] Gao X., Haynes S.W., Ames B.D., Peng W., Vien L.P., Walsh C.T., Tang Y. (2012). Cyclization of Fungal Nonribosomal Peptides by a Terminal Condensation-Like Domain. Nat. Chem. Biol..

[22] Romans-Fuertes P., Sondergaard T.E., Sandmann M.I.H., Wollenberg R.D., Nielsen K.F., Hansen F.T., Giese H., Brodersen D.E., Sørensen J.L. (2016). Identification of the non-ribosomal peptide synthetase responsible for biosynthesis of the potential anti-cancer drug sansalvamide in *Fusarium solani*. Curr. Genet..

[23] Sandargo B., Michehl M., Stadler M., Surup F. (2020). Antifungal sesquiterpenoids, rhodocoranes F-L from submerged cultures of the wrinkled peach mushroom, *Rhodotus palmatus*. J. Nat. Prod..

[24] Alkhalaf L.M., Ryan K.S. (2015). Biosynthetic manipulation of tryptophan in bacteria: Pathways and mechanisms. Chem. Biol..

[25] Therien A.G., Huber J.L., Wilson K.E., Beaulieu P., Caron A., Claveau D., Deschamps K., Donald R.G.K., Galgoci A.M., Gallant M. (2012). Broadening the spectrum of β-lactam antibiotics through inhibition of signal peptidase type I. Antimicrob. Agents Chemother..

[26] Fu P., Jamison M., La S., MacMillan J.B. (2014). Inducamides A-C, chlorinated alkaloids from an RNA polymerase mutant strain of *Streptomyces* sp.. Org. Lett..

[27] Kobayashi J., Satoa M., Ishibashia M., Shigemoria H., Nakamurab T., Ohizumic Y. (1991). Keramamide A, a Novel Peptide from the Okinawan Marine Sponge *Theonella* sp.. J. Chem. Soc. Perkin Trans. 1.

[28] Jansen R., Kunze B., Reichenbach H., Hofle G. (1996). Chondramides A-D, new cytostatic and antifungal cyclodepsipeptides from *Chondromyces crocatus* (Myxobacteria): Isolation and structure elucidation. Liebigs Ann..

[29] Primahana G., Risdian C., Mozef T., Sudarman E., Köck M., Wink J., Stadler M. (2020). Nonocarbolines A–E, β-carboline antibiotics produced by the rare actinobacterium *Nonomuraea* sp. from Indonesia. Antibiotics.

[30] Mohr K.I., Stechling M., Wink J., Wilharm E., Stadler M. (2016). Comparison of myxobacterial diversity and evaluation of isolation success in two niches: Kiritimati Island and German compost. Microbiol. Open.

[31] Hall T.A. (1999). BioEdit: A user-friendly biological sequence alignment editor and analysis program for Windows 95/98/NT. Nucleic Acids. Symp. Ser..

[32] Yoon S.H., Ha S.M., Kwon S., Lim J., Kim Y., Seo H., Chun J. (2017). Introducing EzBioCloud: A taxonomically united database of 16S rRNA gene sequences and whole-genome assemblies. Int. J. Syst. Evol. Microbiol..

[33] Edgar R.C. (2004). MUSCLE: Multiple sequence alignment with high accuracy and high throughput. Nucleic Acids Res..

[34] Kumar S., Stecher G., Li M., Knyaz C., Tamura K. (2018). MEGA X: Molecular evolutionary genetics analysis across computing platforms. Mol. Biol. Evol..

[35] Felsenstein J. (1981). Evolutionary trees from DNA sequences: A maximum likelihood approach. J. Mol. Evol..

[36] Felsenstein J. (1985). Confidence limits on phylogenies: An approach using the bootstrap. Evolution.

[37] Pérez-Bonilla M., Oves-Costales D., González I., de la Cruz M., Martín J., Vicente F., Genilloud O., Reyes F. (2020). Krisynomycins, Imipenem Potentiators against Methicillin-Resistant *Staphylococcus aureus*, Produced by *Streptomyces canus*. J. Nat. Prod..

[38] Wick R.R., Judd L.M., Gorrie C.L., Holt K.E. (2017). Unicycler: Resolving bacterial genome assemblies from short and long sequencing reads. PLoS Comput. Biol..

[39] Medema M.H., Blin K., Cimermancic P., De Jager V., Zakrzewski P., Fischbach M.A., Weber T., Takano E., Breitling R. (2011). AntiSMASH: Rapid identification, annotation and analysis of secondary metabolite biosynthesis gene clusters in bacterial and fungal genome sequences. Nucleic Acids Res..

[40] Altschul S.F., Wootton J.C., Gertz E.M., Agarwala R., Morgulis A., Schäffer A.A., Yu Y.K. (2005). Protein database searches using compositionally adjusted substitution matrices. FEBS J..

[41] Becker K., Wessel A., Luangsa-ard J.J., Stadler M. (2020). Viridistratins A-C, antimicrobial and cytotoxic benzo[j]fluoranthenes from stromata of *Annulohypoxylon viridistratum* (Hypoxylaceae, Ascomycota). Biomolecules.

