# Supplementary material for: Amycolatomycins A and B, Cyclic Hexapeptides Isolated from an Amycolatopsis sp. 195334CR
Source: Antibiotics (Basel). 2021 Mar 5;10(3):261. doi: 10.3390/antibiotics10030261 (PMC8002008; doi:10.3390/antibiotics10030261)
Supplement: Supplementary file 1 [file antibiotics-10-00261-s001.pdf]

Supporting Information

Amycolatomycins A and B, Cyclic Hexapeptides Isolated from an *Amycolatopsis* sp. 195334CR

Gian Primahana [1,2,‡], Chandra Risdian [3,4,‡], Tjandrawati Mozef [2], Joachim Wink [3], Frank Surup [1] and Marc Stadler [1,*]

[1]Department Microbial Drugs, Helmholtz Centre for Infection Research GmbH (HZI), Inhoffenstrasse 7, 38124 Braunschweig, Germany; Gian.Primahana@helmholtz-hzi.de (G.P.); Frank.Surup@helmholtz-hzi.de (F.S)

[2]Research Center for Chemistry, Indonesian Institute of Sciences (LIPI), Kawasan Puspiptek, Serpong, 15314 Tangerang Selatan, Indonesia

[3]Working group Microbial Strain Collection, Helmholtz Centre for Infection Research GmbH (HZI), Inhoffenstrasse 7, 38124 Braunschweig, Germany; Chandra.Risdian@helmholtz-hzi.de (C. R.); Joachim.Wink@helmholtz-hzi.de (J.W.)

[4]Research Unit for Clean Technology, Indonesian Institute of Sciences (LIPI), Bandung 40135, Indonesia

[‡]Author contributed equally

Correspondence: Prof. Dr. Marc Stadler, Department Microbial Drugs, Helmholtz Centre for Infection Research GmbH (HZI), Inhoffenstrasse 7, 38124 Braunschweig, Germany

Email: Marc.Stadler@helmholtz-hzi.de

Table of contents

Figure S1. *Amycolatopsis* sp. 195334CR on GYM agar plate 4

Figure S2. The phylogenetic tree based on the nearly complete 16S rRNA gene sequence 4

Figure S3. HPLC-DAD/MS chromatogram of amycolatomycin A 5

Figure S4. HR-ESIMS chromatogram of amycolatomycin A 5

Figure S5. UV/vis spectrum of amycolatomycin A in MeOH 5

Figure S6. ^{1}H NMR spectrum of amycolatomycin A in DMSO-d_6 (700 MHz) 6

Figure S7. ^{13}C NMR spectrum of amycolatomycin A in DMSO-d_6 (176 MHz) 7

Figure S8. ^{1}H,^{1}H COSY NMR spectrum of amycolatomycin A in DMSO-d_6 8

Figure S9. ^{1}H,^{13}C HSQC-DEPT NMR spectrum of amycolatomycin A in DMSO-d_6 (700 MHz, 176 MHz) 9

Figure S10. ^{1}H,^{13}C HMBC NMR spectrum of amycolatomycin A in DMSO-d_6 (700 MHz, 176 MHz) 10

Figure S11. TOCSY NMR spectrum of amycolatomycin A in DMSO-d_6 (700 MHz) 11

Figure S12. ROESY NMR spectrum of amycolatomycin A in DMSO-d_6 (700 MHz) 12

Figure S13. ^{1}H NMR spectrum of amycolatomycin A in CD_3OD (700 MHz) 13

Figure S14. ^{13}C NMR spectrum of amycolatomycin A in CD_3OD (700 MHz) 14

Figure S15. ^{1}H,^{1}H COSY NMR spectrum of amycolatomycin A in CD_3OD 15

Figure S16. ^{1}H,^{13}C HSQC-DEPT NMR spectrum of amycolatomycin A in CD_3OD (700 MHz, 176 MHz) 16

Figure S17. ^{1}H,^{13}C HMBC NMR spectrum of amycolatomycin A in CD_3OD (700 MHz, 176 MHz) 17

Figure S18. ROESY NMR spectrum of amycolatomycin A in CD_3OD (700 MHz) 18

Table 1. ^{1}H NMR and ^{13}C NMR of amycolatomycin A in CD_3OD (^{1}H 700 MHz; ^{13}C 176 MHz) 19

Figure S19. Reaction scheme of hydrolysis amycolatomycin A under acidic condition 21

Figure S20. HPLC-DAD/MS chromatogram of hydrolysis amycolatomycin A under an acidic condition 21

Figure S21. HPLC-DAD/MS of partial degradation of linear amycolatomycin under an acidic condition at 100 °C 22

Figure S22. Partial degradation scheme of linear amycolatomycin A under acidic condition at 100 °C 23

Figure 23. General reaction of Marfey's reagent and amino acid 24

Table 2. Retention time of L or D authentic amino acid derived D-FDVA 24

Table 3. Retention time of L or D authentic amino acid derived L-FDVA 24

Figure S24. HPLC-DAD/MS chromatogram of L/D or DL authentic amino acid derived D-FDVA.. 25

Figure S25. HPLC-DAD/MS chromatogram of L/D/DL authentic amino acid derived L-FDVA 26

Figure S26. HPLC-DAD/MS of Amycolatomycin A catalyzed by $RuCl_3 \cdot H_2O$-$NaIO_4$ followed by hydrolysis and derivatization with L-FDVA on C_{18} column. 27

Figure S27. HPLC-DAD/MS of Marfey's analysis on C_4 column of an authentic amino acid L and L-*allo*-Ile: co-injection of D-FDVA-L-Ile-L-*allo*-Ile (a), D-FDVA-L-Ile (b), and D-FDVA-L-*allo*-Ile (c) 28

.... 28

Figure S28. HPLC-DAD/MS of Marfey's analysis of amycolatomycin A derived D-FDVA in C_{18} column 29

Figure S29. HPLC-DAD/MS of Amycolatomycin A catalyzed by $RuCl_3 \cdot H_2O$-$NaIO_4$ followed by hydrolysis and derivatization with D-FDVA on C_{18} column. 30

Figure S30. HPLC-DAD/MS chromatogram of amycolatomycin B 31

Figure S31. HR-ESIMS chromatogram of amycolatomycin B 31

Figure S32. UV/vis spectrum of amycolatomycin B in MeOH 31

Figure S33. ^1H NMR spectrum of amycolatomycin B in DMSO-d_6 (700 MHz) 32

Figure S34. ^{13}C NMR spectrum of amycolatomycin B in DMSO-d_6 (176 MHz) 33

Figure S35. $^1H,^1H$ COSY NMR spectrum of amycolatomycin B in DMSO-d_6 34

Figure S36. $^1H,^{13}C$ HSQC-DEPT NMR spectrum of amycolatomycin B in DMSO-d_6 (700 MHz, 176 MHz) 35

Figure S37. $^1H,^{13}C$ HMBC NMR spectrum of amycolatomycin B in DMSO-d_6 (700 MHz, 176 MHz) 36

Figure S38. TOCSY NMR spectrum of amycolatomycin B in DMSO-d_6 (700 MHz) 37

Figure S39. ROESY NMR spectrum of amycolatomycin B in DMSO-d_6 (700 MHz) 38

Figure S40. HPLC-DAD/MS of Marfey's analysis on C_4 column of amycolatomycin A derived D-FDVA 39

Figure S41. HPLC-DAD/MS of Marfey's analysis on C_4 column of amycolatomycin B derived D-FDVA 40

Figure S42. Isolation process chromatogram of GF2 fraction in preparative HPLC 41

Table S4. List of amycolatomycin biosynthetic genes and its nucleotide sequence (*ammA – ammD*) 42

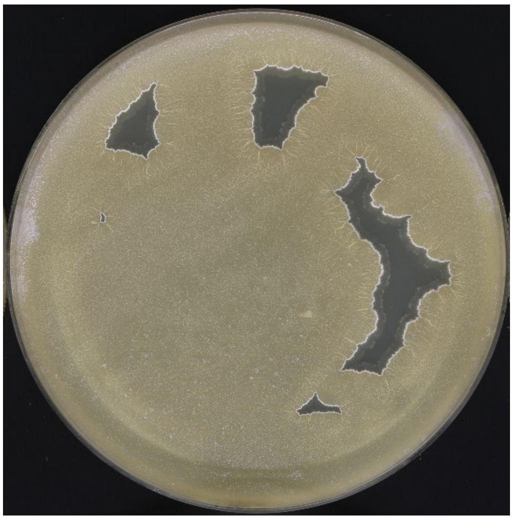

Figure S1. *Amycolatopsis* sp. 195334CR on GYM agar plate

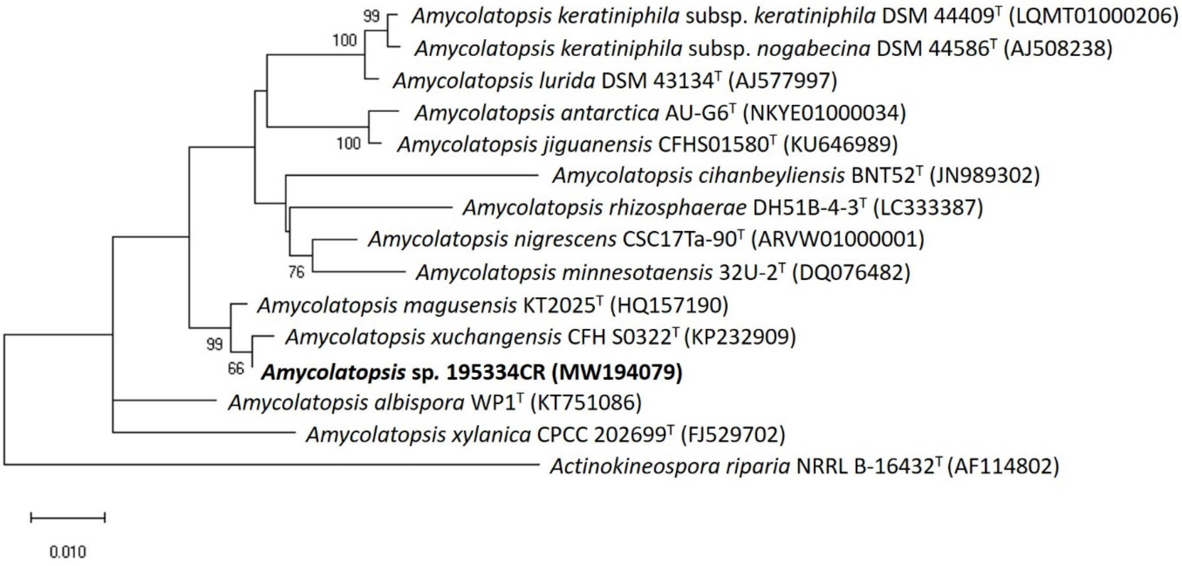

Figure S2. The phylogenetic tree based on the nearly complete 16S rRNA gene sequence (1385 positions in the final dataset) of strain 195334CR and closely related type strains of the genus Amycolatopsis based on Maximum Likelihood method and Tamura-Nei model using Actinokineospora riparia DSM 44259 as an out group. A discrete Gamma distribution was used to model evolutionary rate differences among sites (5 categories (+G, parameter = 0.3922)). The rate variation model allowed for some sites to be evolutionarily invariable ([+I], 67.43% sites). Numbers at the nodes are percentage bootstrap values with 100 replicates; only values above 50% are shown. Bar 0.010 substitutions per nucleotide position. Evolutionary analyses were conducted in MEGA X.

Amycolatopsis sp. strain 195334CR was isolated from a soil sample collected at Bali, Indonesia. The 16S rRNA gene sequences analysis indicated that the aligned sequenced was closely related to the DNA sequence of the type strain *Amycolatopsis magusensis* (DSM 45510^{T}) with 99.37% similarity and *Amycolatopsis xuchangensis* (similarity 99.72%)

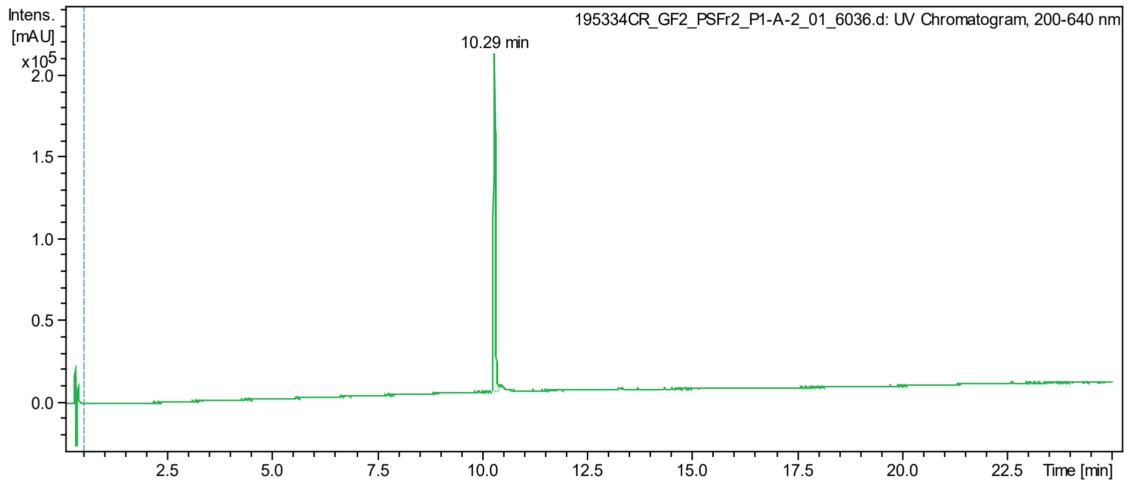

Figure S3. HPLC-DAD/MS chromatogram of amycolatomycin A

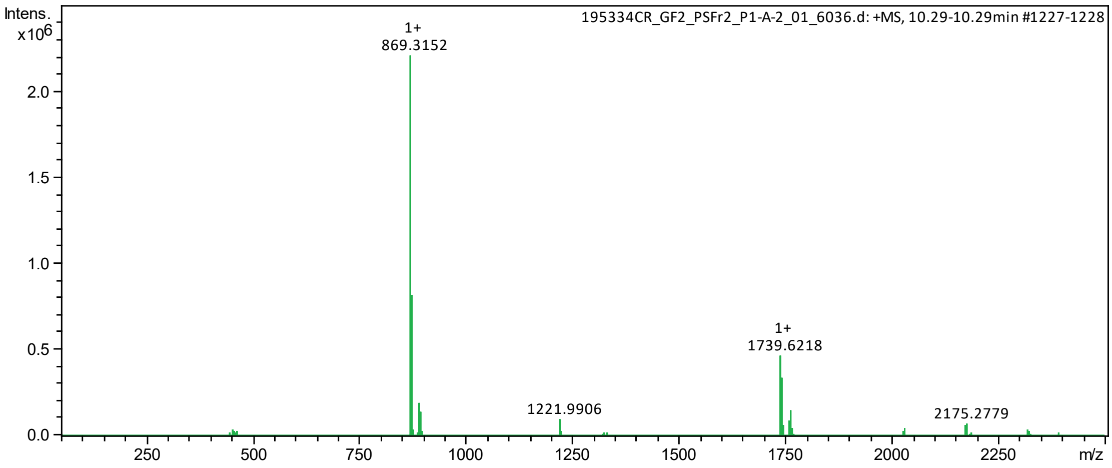

Figure S4. HR-ESIMS chromatogram of amycolatomycin A

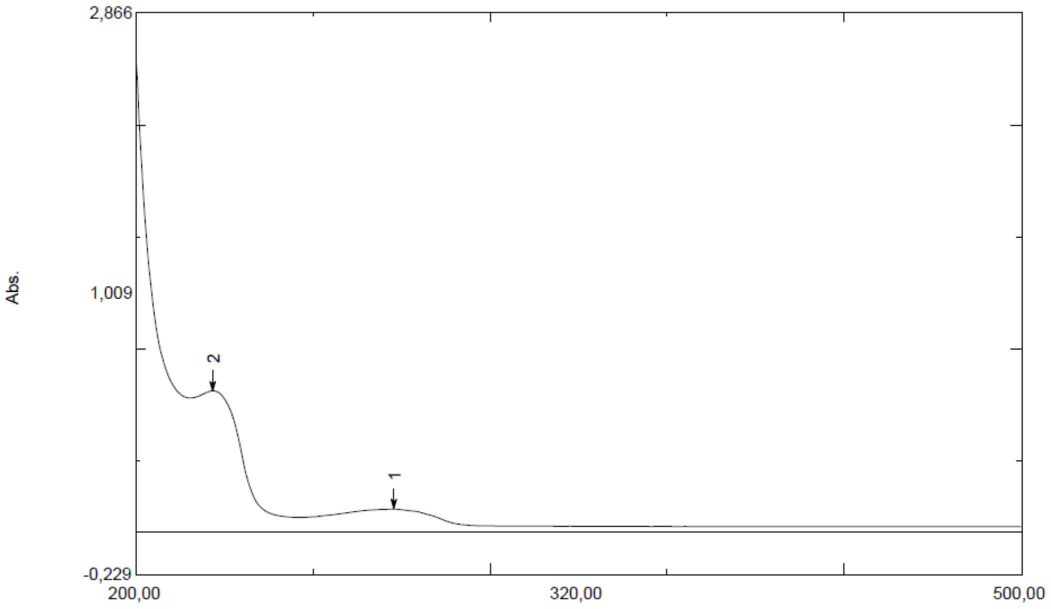

Figure S5. UV/vis spectrum of amycolatomycin A in MeOH (log ε) [neutral]λ_{max} 226 (4.83), 287 (4.03) nm.

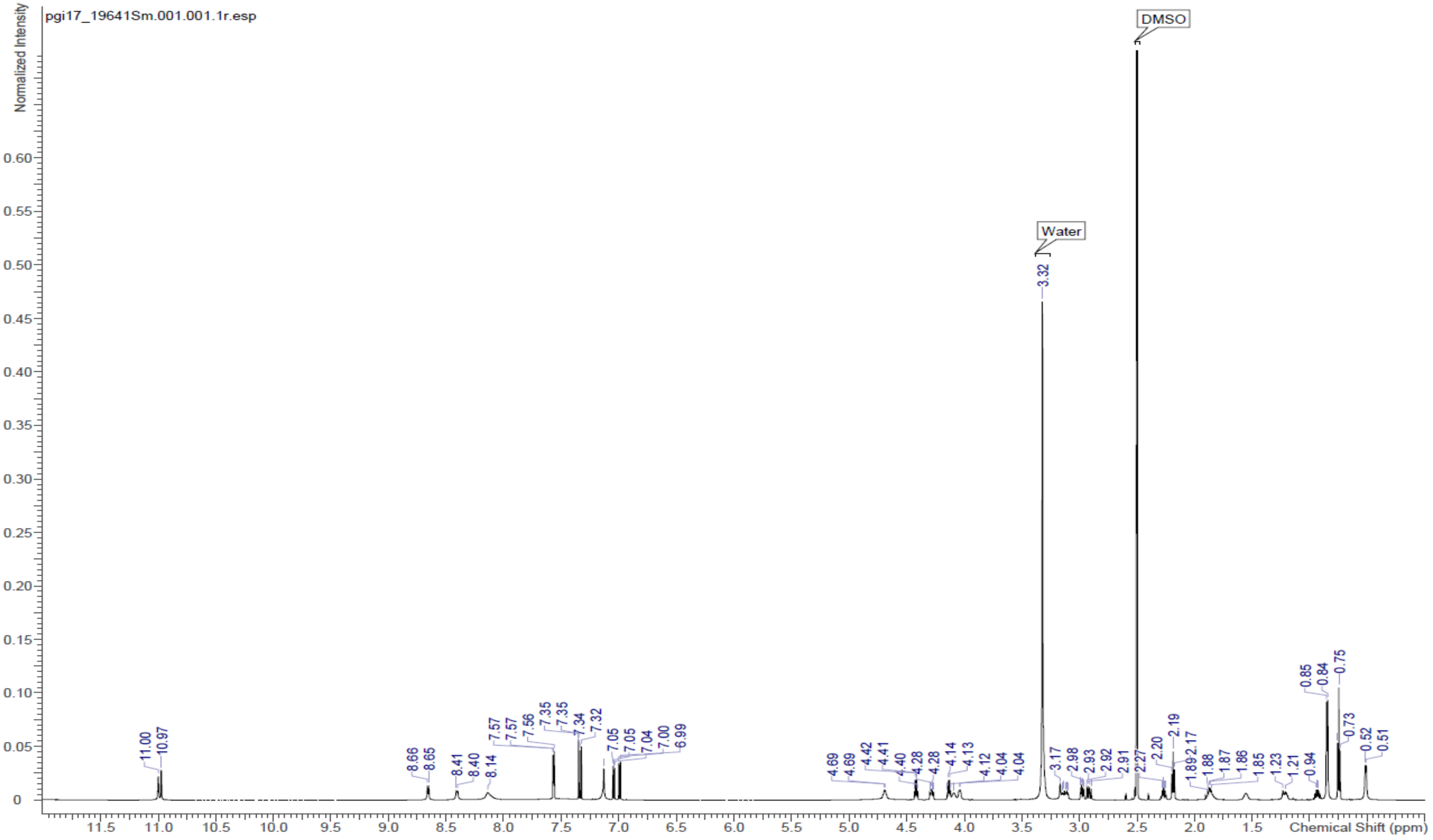

Figure S6. ^{1}H NMR spectrum of amycolatomycin A in DMSO-d_6 (700 MHz)

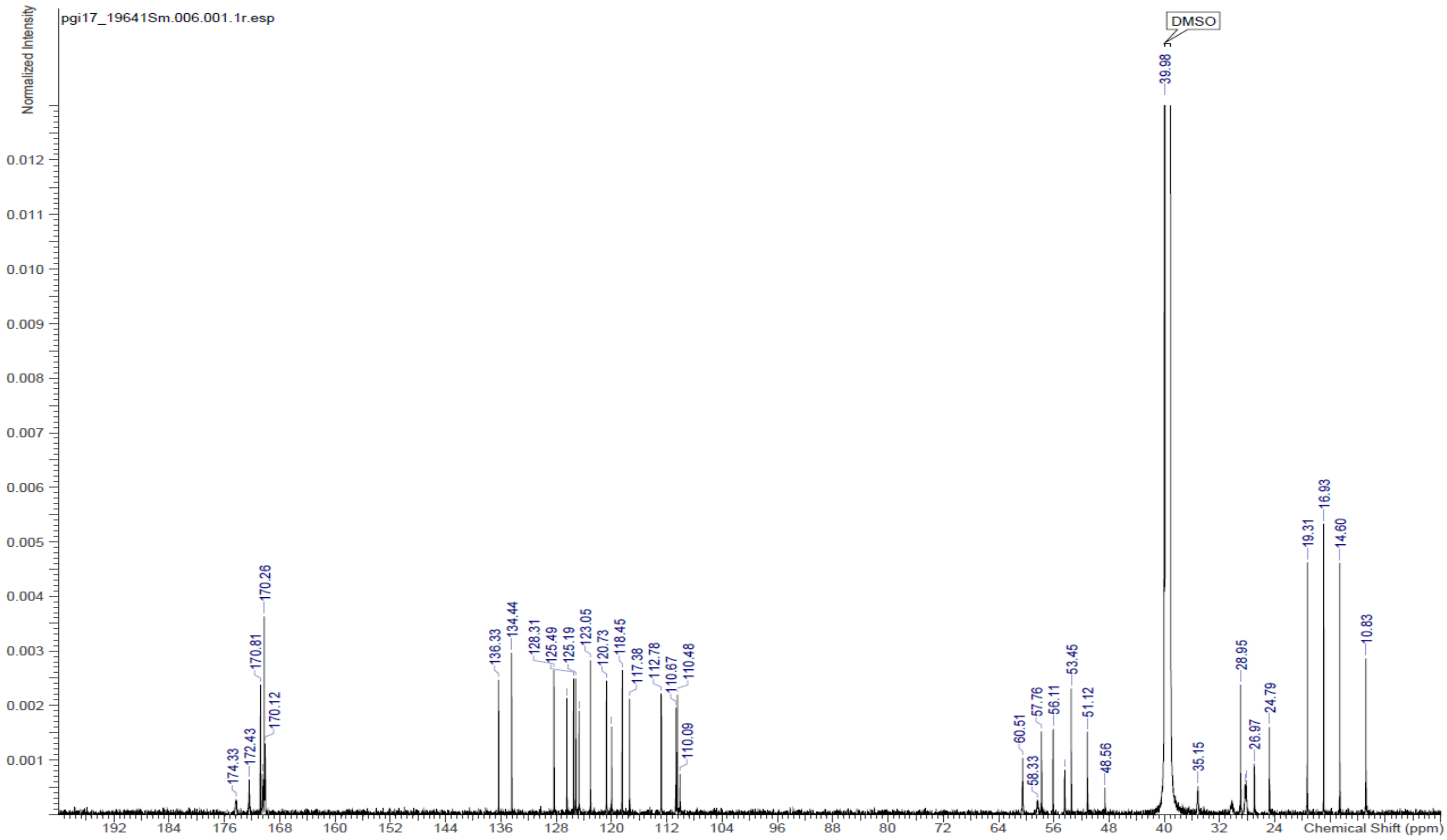

Figure S7. ^{13}C NMR spectrum of amycolatomycin A in DMSO-d_6 (176 MHz)

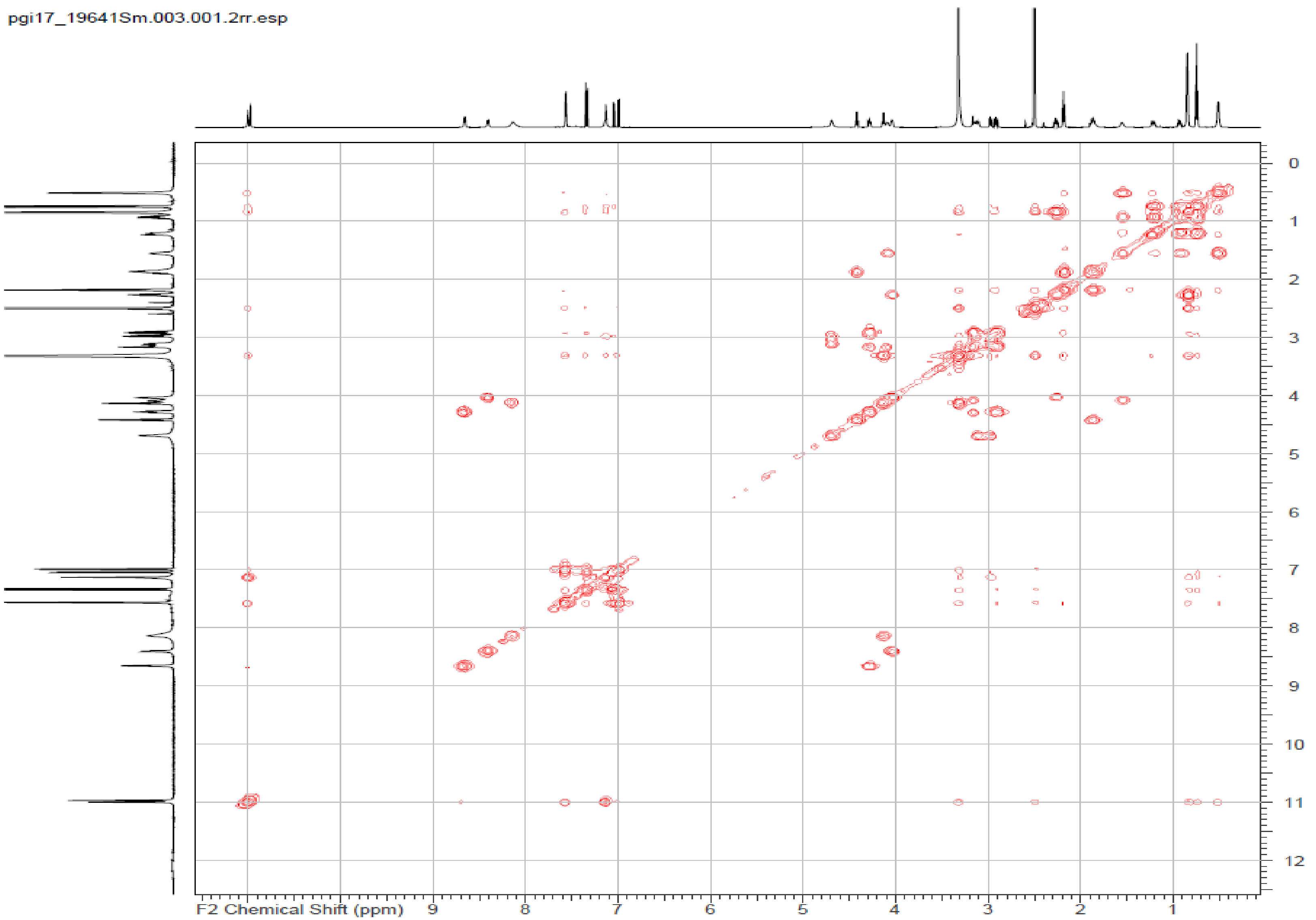

Figure S8. $^{1}H,^{1}H$ COSY NMR spectrum of amycolatomycin A in DMSO-d_6

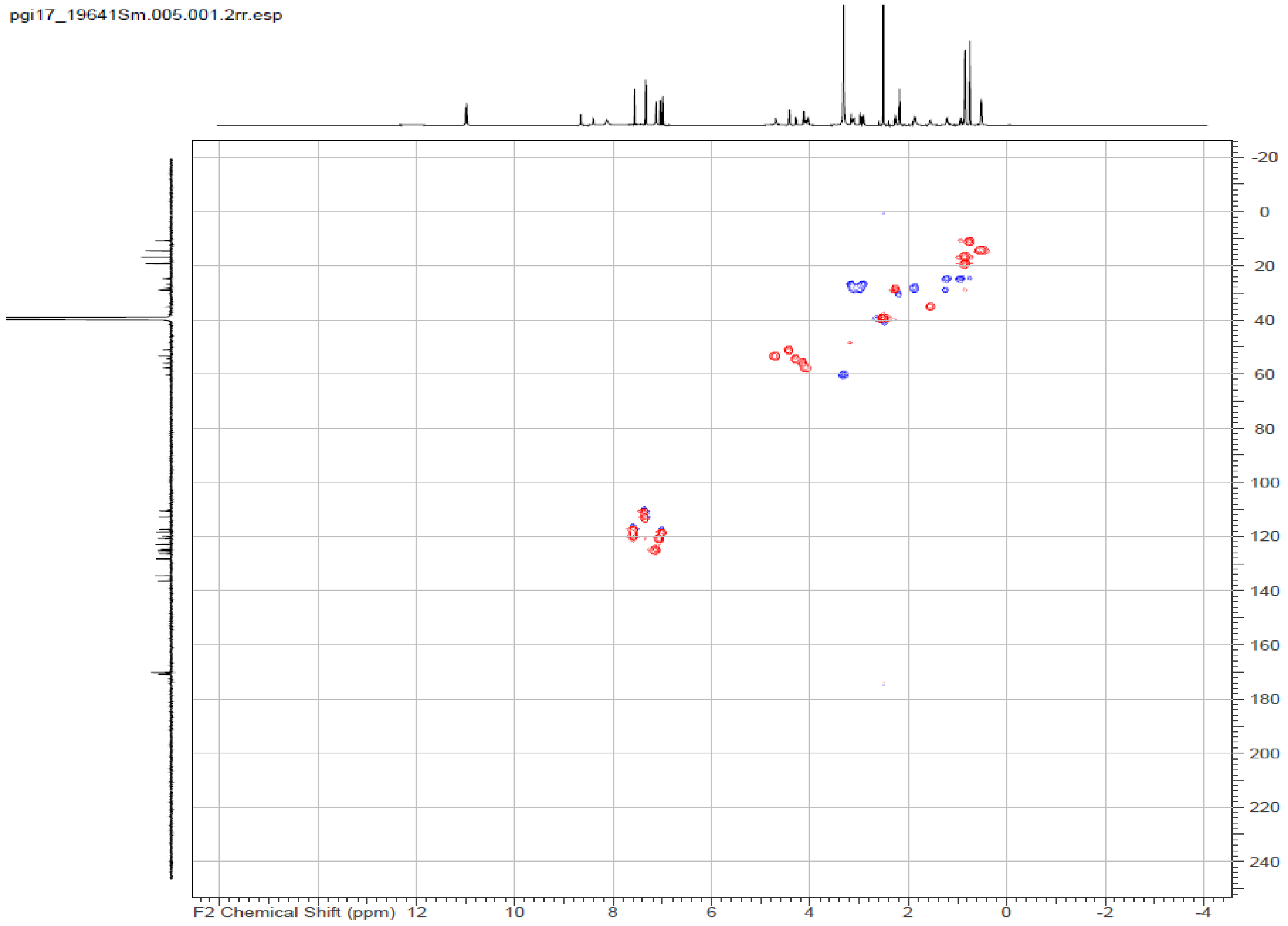

Figure S9. $^{1}H,^{13}C$ HSQC-DEPT NMR spectrum of amycolatomycin A in DMSO-d_6 (700 MHz, 176 MHz)

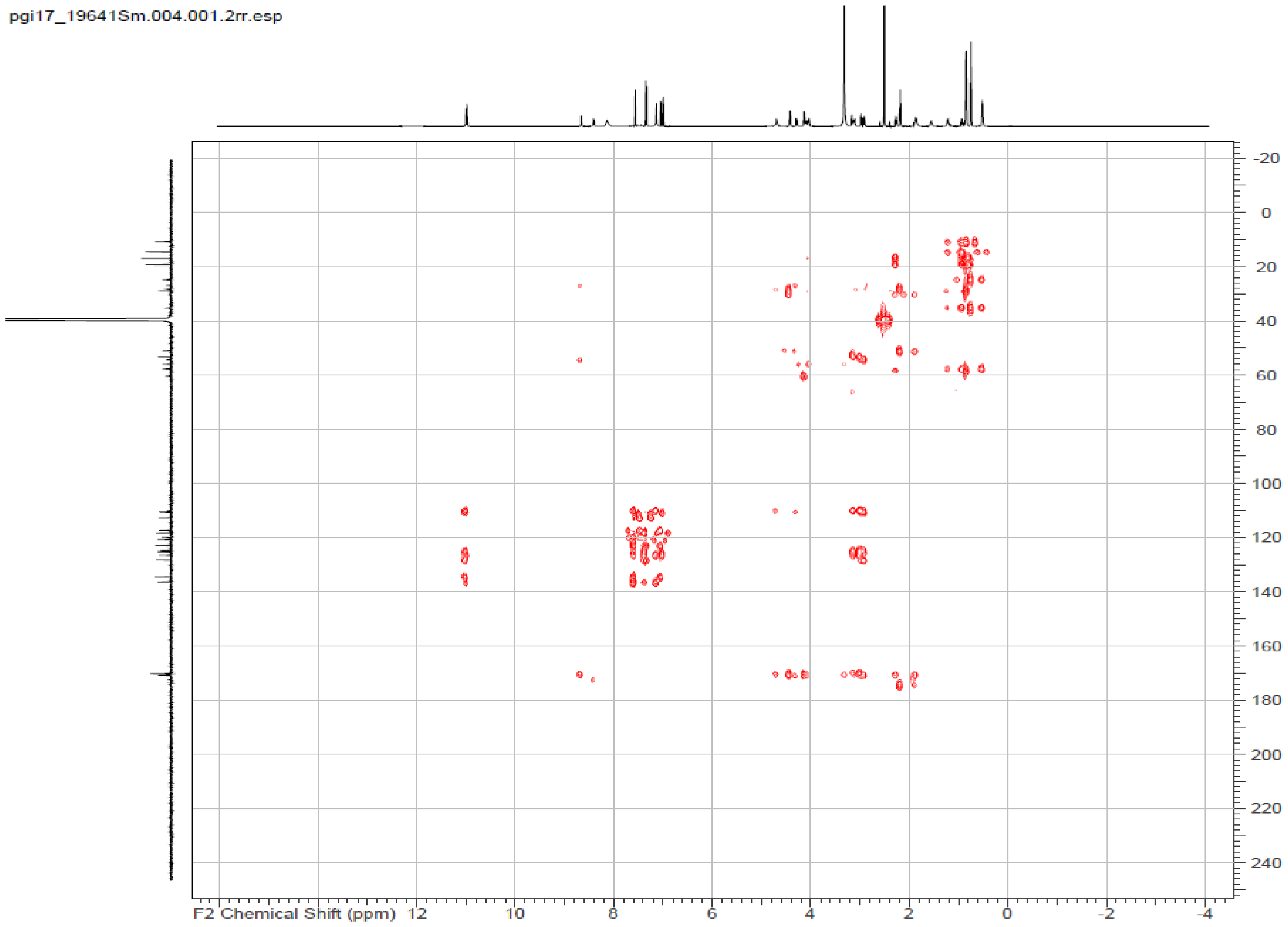

Figure S10. ^{1}H,^{13}C HMBC NMR spectrum of amycolatomycin A in DMSO-*d₆* (700 MHz, 176 MHz)

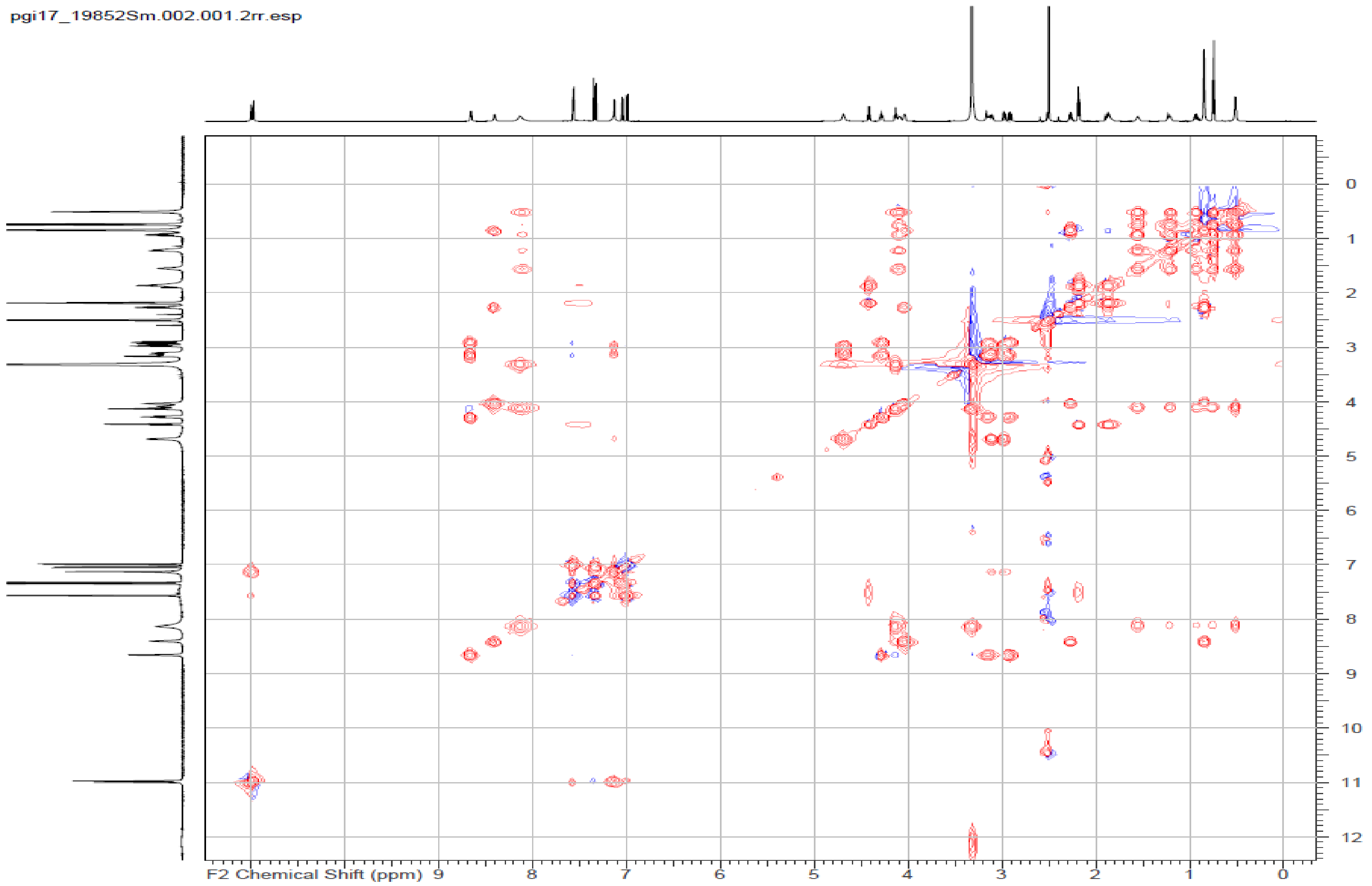

Figure S11. TOCSY NMR spectrum of amycolatomycin A in DMSO-d_6 (700 MHz)

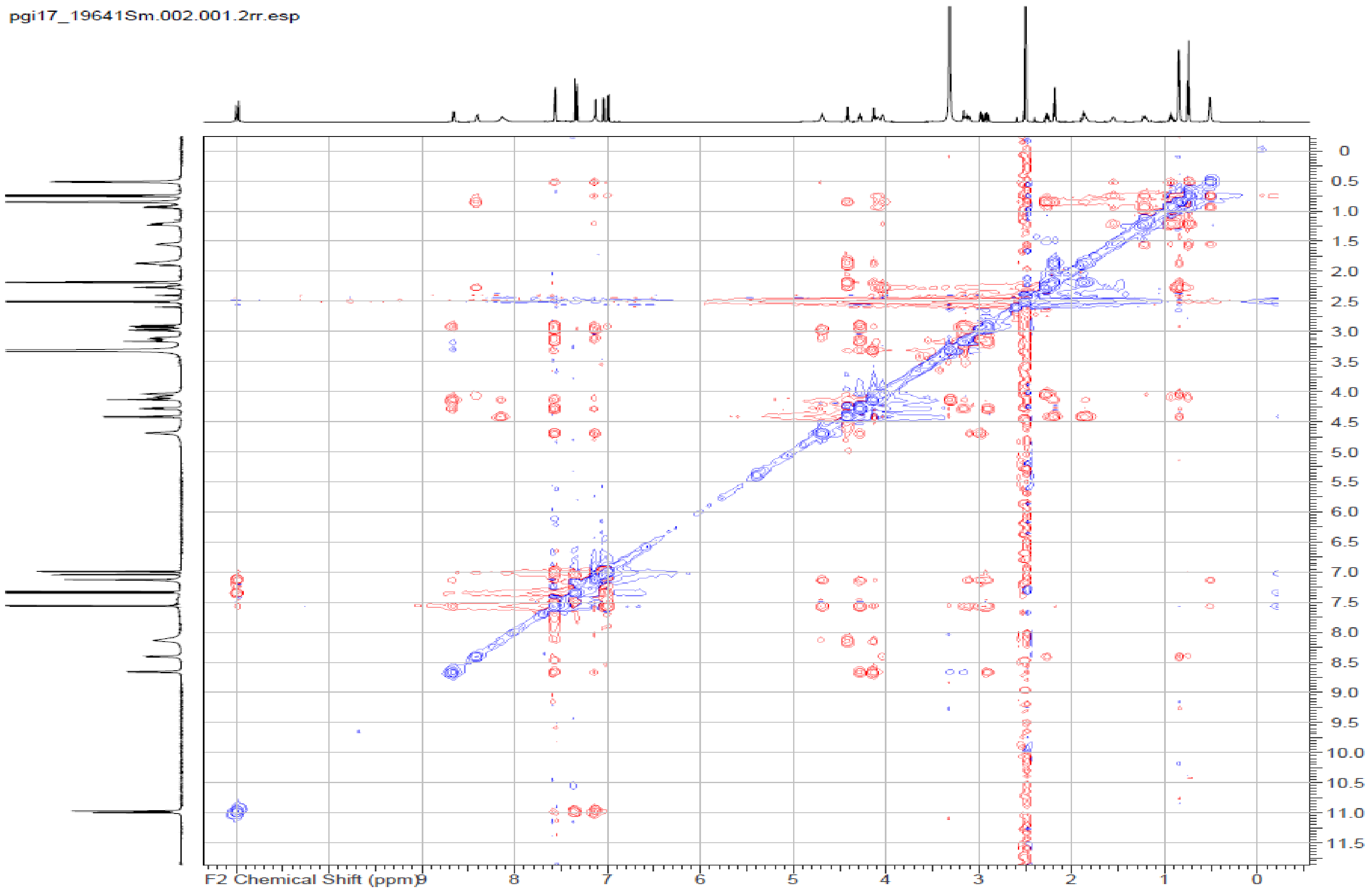

Figure S12. ROESY NMR spectrum of amycolatomycin A in DMSO-d_6 (700 MHz)

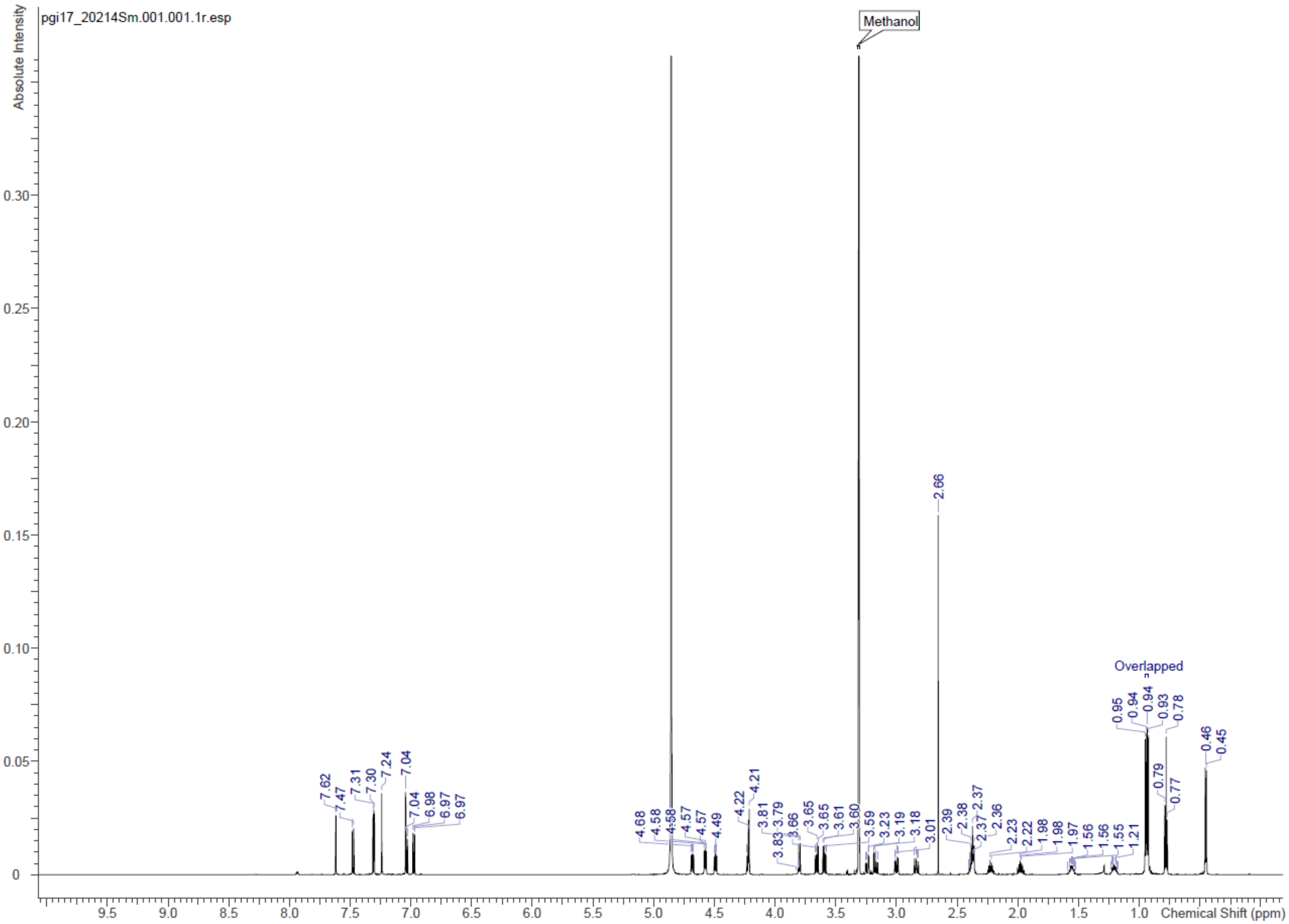

Figure S13. ^{1}H NMR spectrum of amycolatomycin A in CD_3OD (700 MHz)

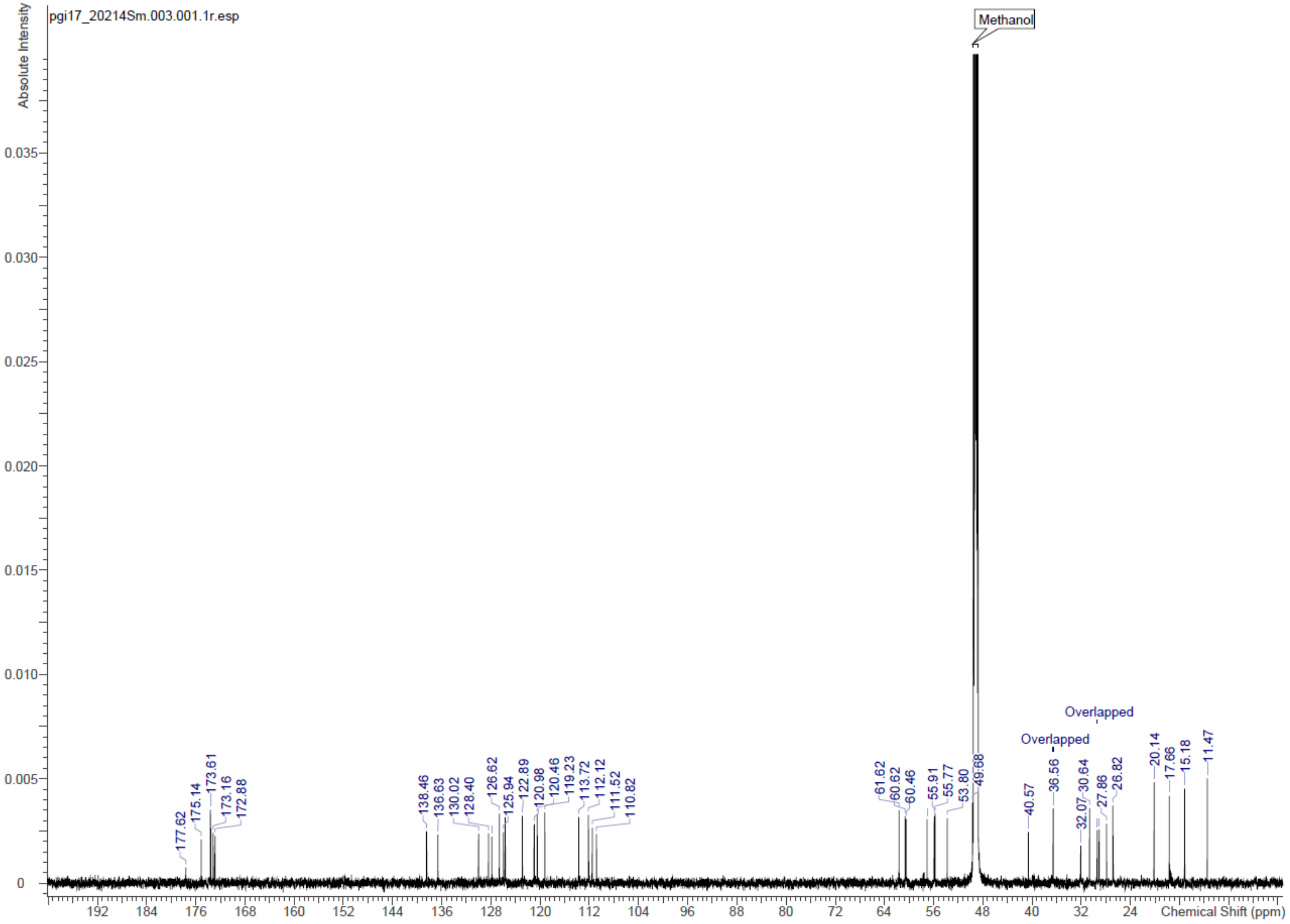

Figure S14. ^{13}C NMR spectrum of amycolatomycin A in CD_3OD (700 MHz)

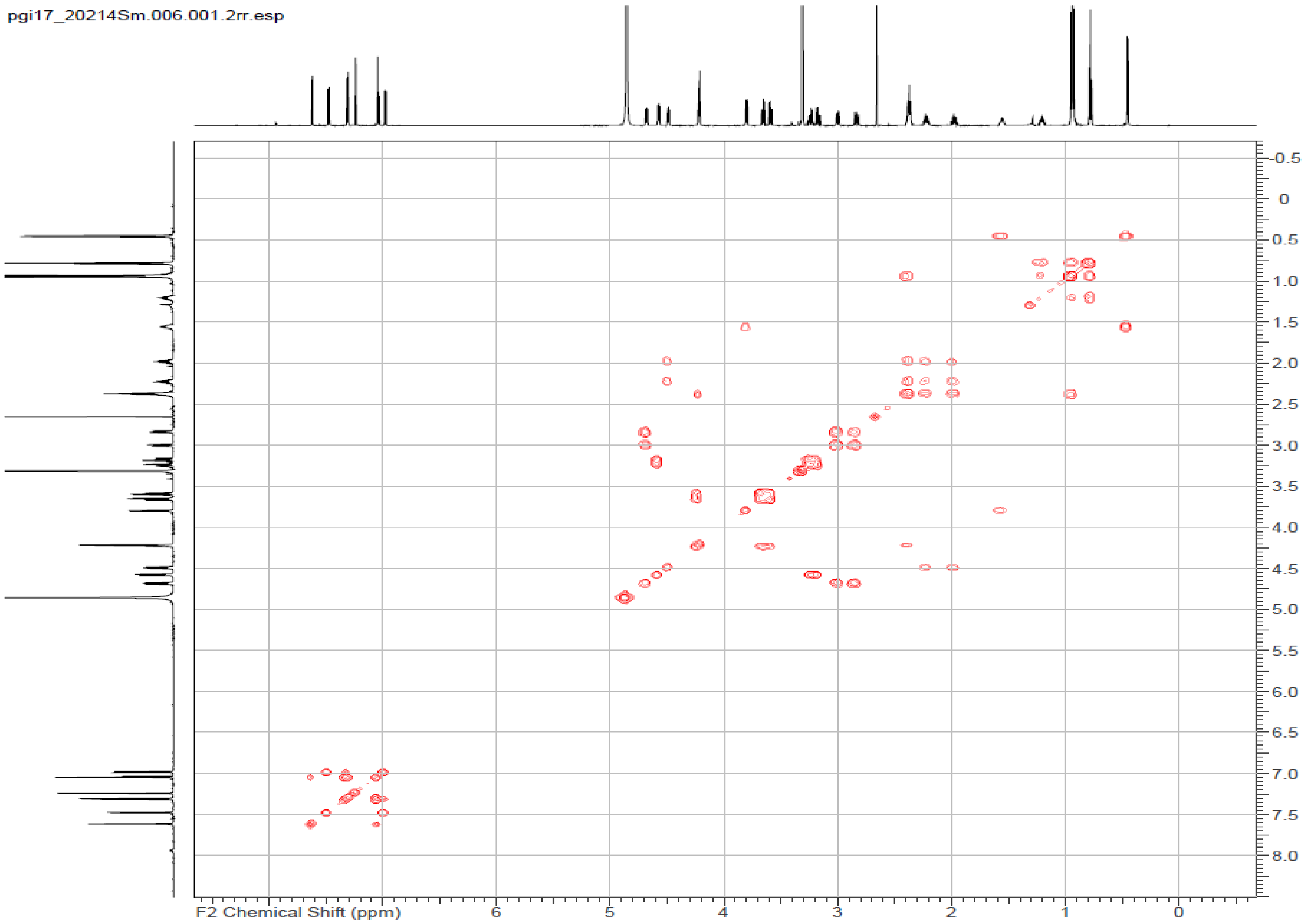

Figure S15. ^{1}H,^{1}H COSY NMR spectrum of amycolatomycin A in CD_3OD

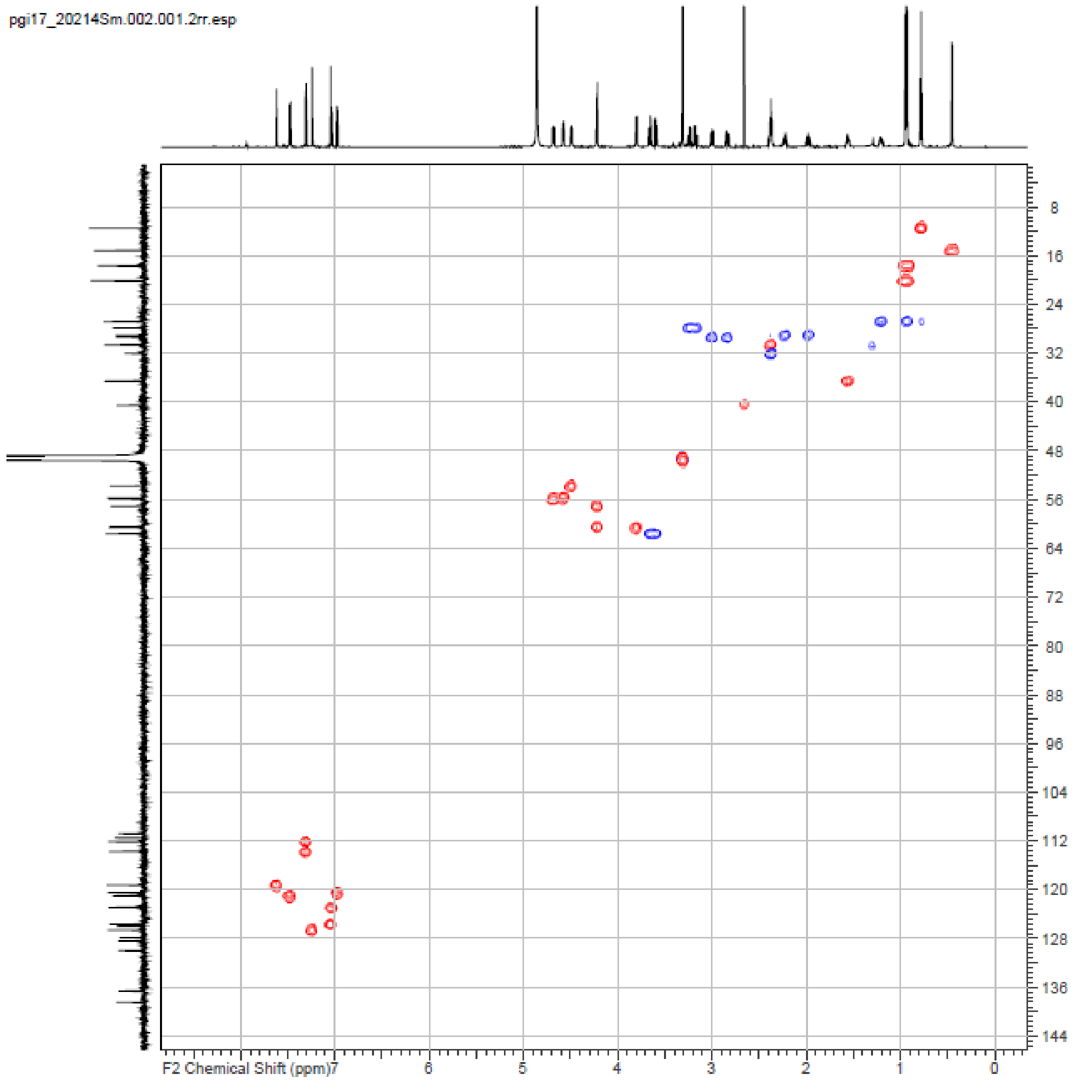

Figure S16. $^{1}H,^{13}C$ HSQC-DEPT NMR spectrum of amycolatomycin A in CD_3OD (700 MHz, 176 MHz)

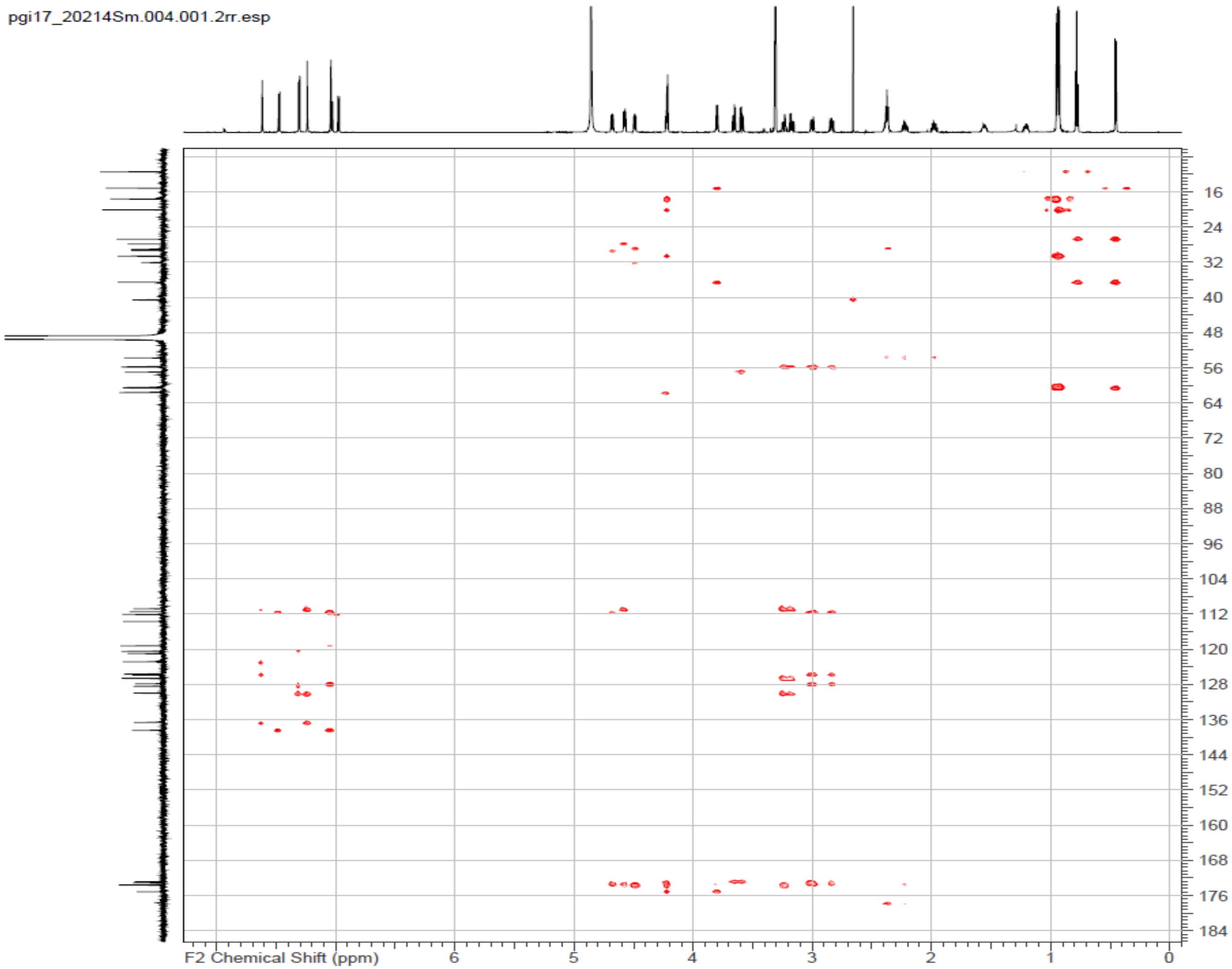

Figure S17. ^{1}H,^{13}C HMBC NMR spectrum of amycolatomycin A in CD_3OD (700 MHz, 176 MHz)

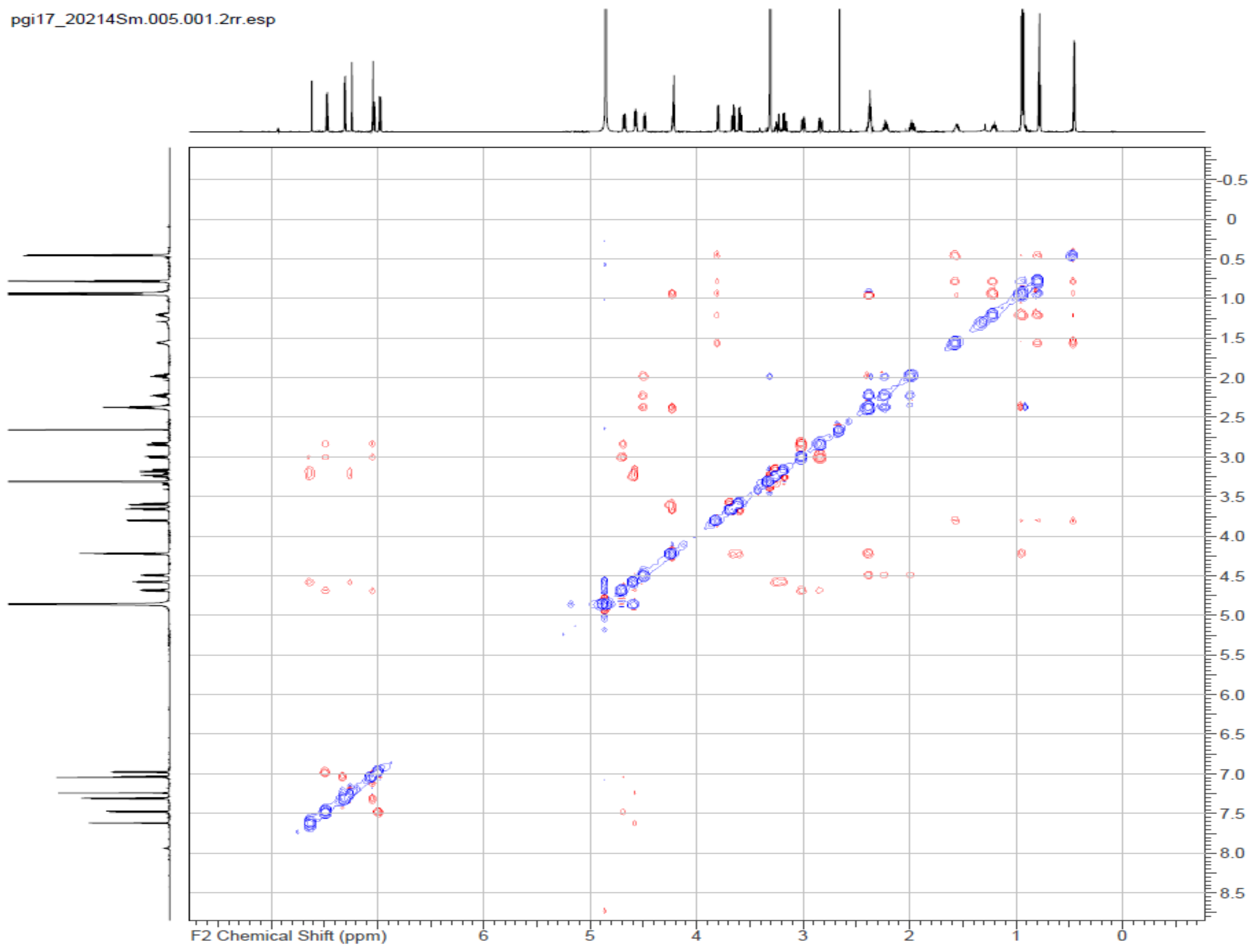

Figure S18. ROESY NMR spectrum of amycolatomycin A in CD_3OD (700 MHz)

Table 1. ^{1}H NMR and ^{13}C NMR of amycolatomycin A in CD_3OD (^{1}H 700 MHz; ^{13}C 176 MHz)

Unit	Pos	δ_H, mult (J in Hz)	δ_C
dcT	1	-	175.1, C
	2	4.57, dd (8.0; 4.4)	55.8, CH
	2NH	-	-
	3	3.24, m; 3.17, m	27.9, CH_2
	4	-	110.8, C
	5-Cl	-	128.4, C
	6NH	-	-
	7	-	136.6, C
	8	7.62, d (2.0)	119.2, CH
	9-Cl	-	125.9, C
	10	7.04, m^{ov}	122.9, CH
	11	7.24, s (br)	126.6, CH
	12	-	130.0, C
Trp	1	-	173.6, C
	2	4.68, dd (9.0; 5.5)	55.9, CH
	2NH	-	-
	3	3.00, m; 2.84, dd (14.0, 5.6)	29.4, CH_2
	4	-	111.5, C
	5	7.04, s	125.7, CH
	6NH	-	-
	7	-	138.5, C
	8	7.48, d (8.4)	121.0, CH
	9	6.98, dd (8.5, 2.0)	120.5, CH
	10	7.30, m^{ov}	112.1, C
	11	7.31, m^{ov}	113.7, CH
	12	-	127.8, C

Unit	Pos	δ_H, mult (J in Hz)	δ_C
Val	1	-	173.2, C
	2	4.22, m^{ov}	60.5, CH
	2NH	-	-
	3	2.41, m	30.6, CH
	4	0.95, d (6.8)^{ov}	26.8, CH_3
	5	0.93, d (6.8)^{ov}	20.1, CH_3
Ser	1	-	172.9, C
	2	4.23, m^{ov}	57.1, CH
	2NH	-	-
	3	3.66; 3.59, dd (11.0, 7.0)	61.6, CH_2
Glu	1	-	173.6, C
	2	4.49, dd (8.4, 5.4)	53.8, CH
	2NH	-	-
	3	2.22; 1.98, m	29.1, CH_2
	4	2.37; 2.38, m	32.1, CH_2
	5	-	177.6, C
Ile	1	-	173.6, C
	2	3.80, d (8.4)	60.6, CH
	2NH	-	-
	3	1.56, m	36.6, CH
	4	1.21, m	26.8, CH_2
	5	0.78, t (7.4)	11.5, CH_3
	6	0.45, d (6.7)	15.2, CH_3

ov: overlapped signal

Structure elucidation:

^{1}H-NMR, ^{13}C:NMR, HSQC-DEPT, HMBC, COSY, TOCSY and ROESY in CD_3OD of compound **1** allowed us to identify fragments of amino acids Val, Ser, Glu, Ile, dcT (2,6-dichloro tryptophan), and Trp. Key analyses of amino acid sequences were as follows. Ser was positioned next to Glu by an HMBC signal of H-2 Ser (4.23 ppm) to C-1 of Glu at 173.6 ppm. An HMBC signal from H-2 Val (4.22 ppm) to C-1 of Ser (172.9 ppm) allowed us to connect Ser next to Val. Tryptophan was connected next to Val deduced from an HMBC signal of H-2 Trp (4.68 ppm) to a carbon signal of Val at 173.2 ppm. The connectivity between Trp and 2,6-dichloro tryptophan (dcT) was conclude from an HMBC signal of H-2 dcT (4.57 ppm) and H-2 Trp (4.68 ppm) to C-1 of Trp at 173.6 ppm. 2,6-dichloro tryptophan was connected next to Ile from an HMBC signal of H-2 Ile (3.80 ppm) to C-1 of dcT at 175.1 ppm. And finally an HMBC signal from H-2 of Glu (4.49 ppm) to carbon signal at 173.6 (C-1 of Ile) completing the cyclic structure of amycolatomycin A.

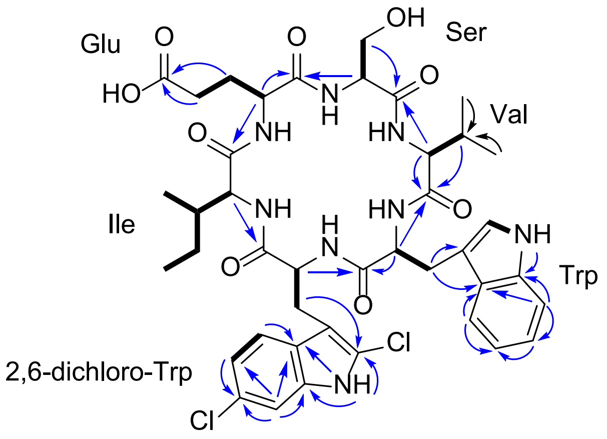

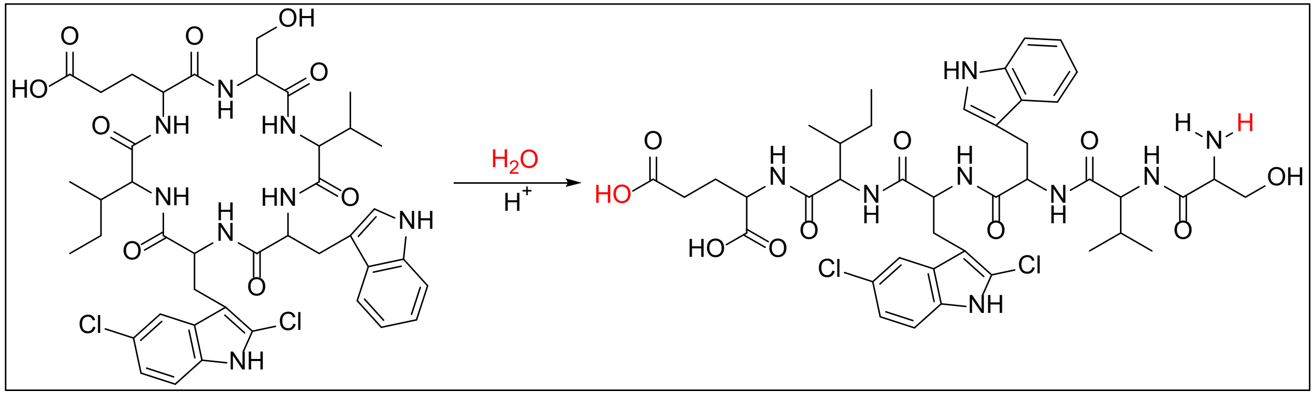

Figure S19. Reaction scheme of hydrolysis amycolatomycin A under acidic condition

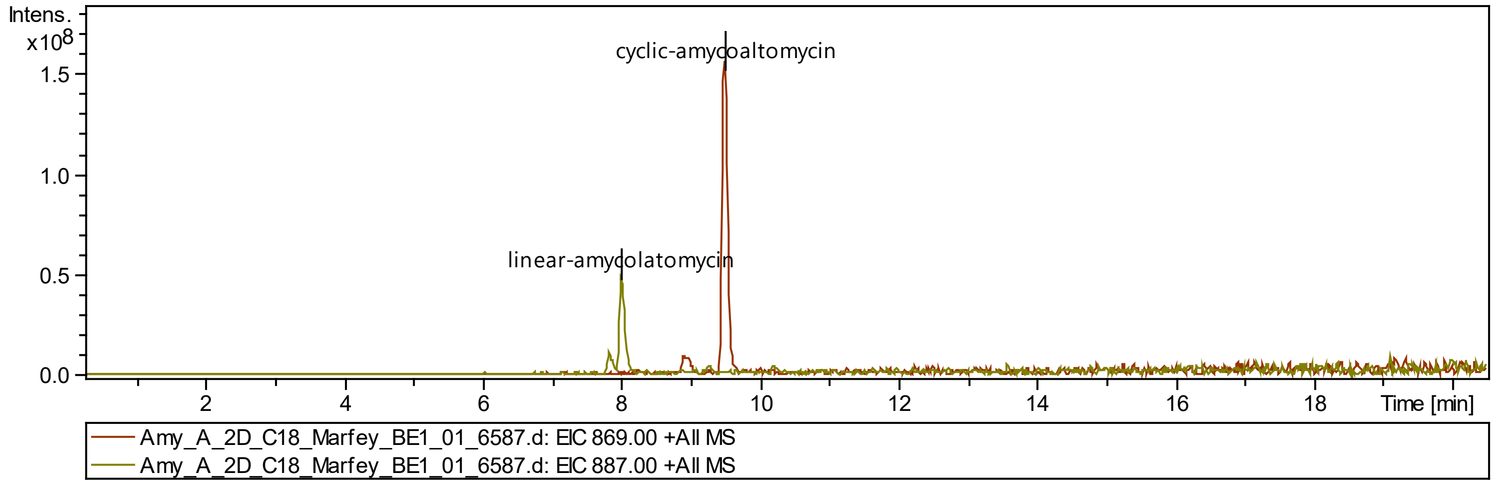

Figure S20. HPLC-DAD/MS chromatogram of hydrolysis amycolatomycin A under an acidic condition (chromatogram showed EIC for cyclic amycoaltomycin A at m/z 869 $[M+H]^+$ and m/z 887 $[M+H]^+$ for linear amycolatomycin A)

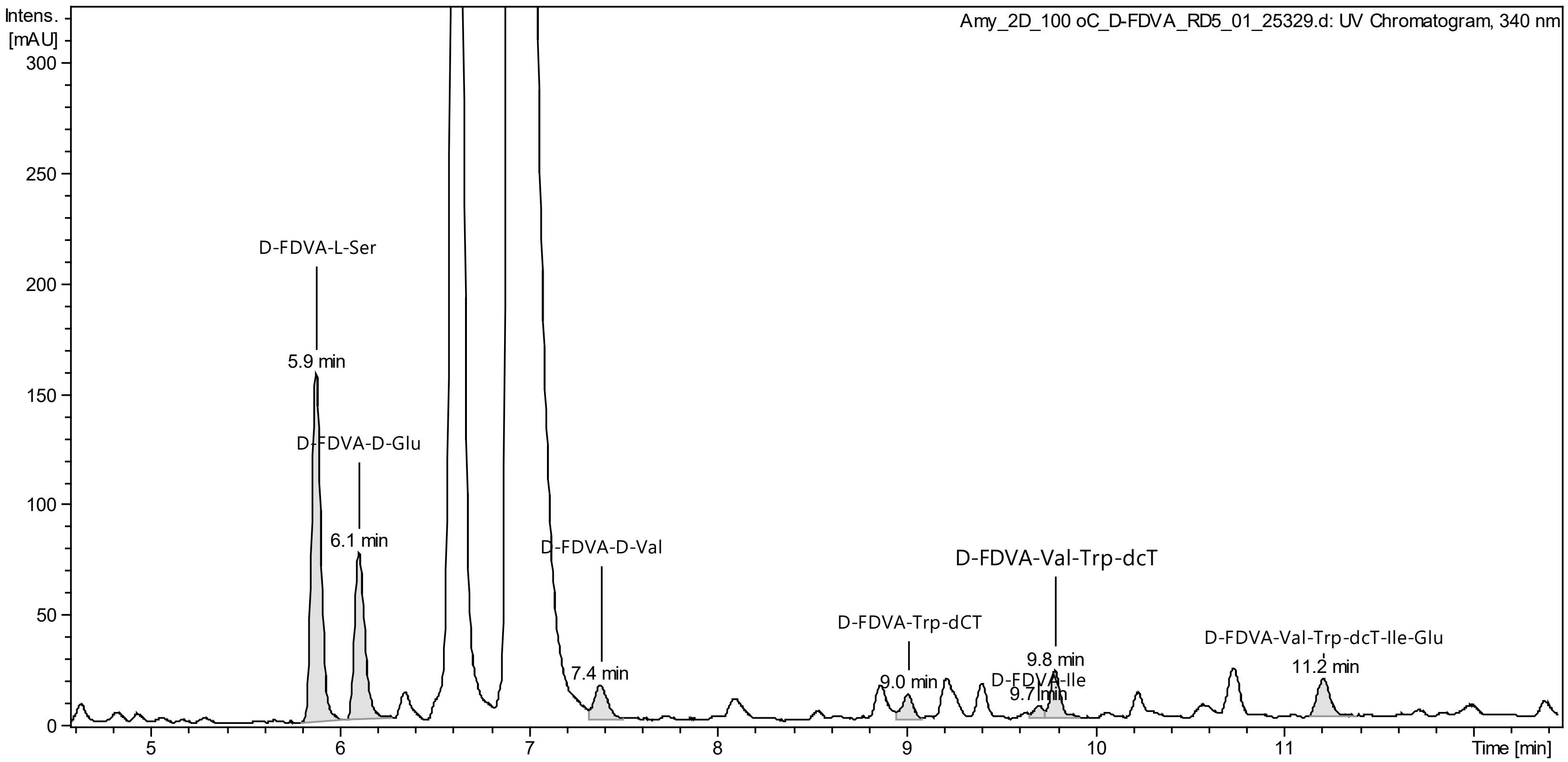

Figure S21. HPLC-DAD/MS of partial degradation of linear amycolatomycin under an acidic condition at 100 ºC (UV chromatogram selected at 340 nm)

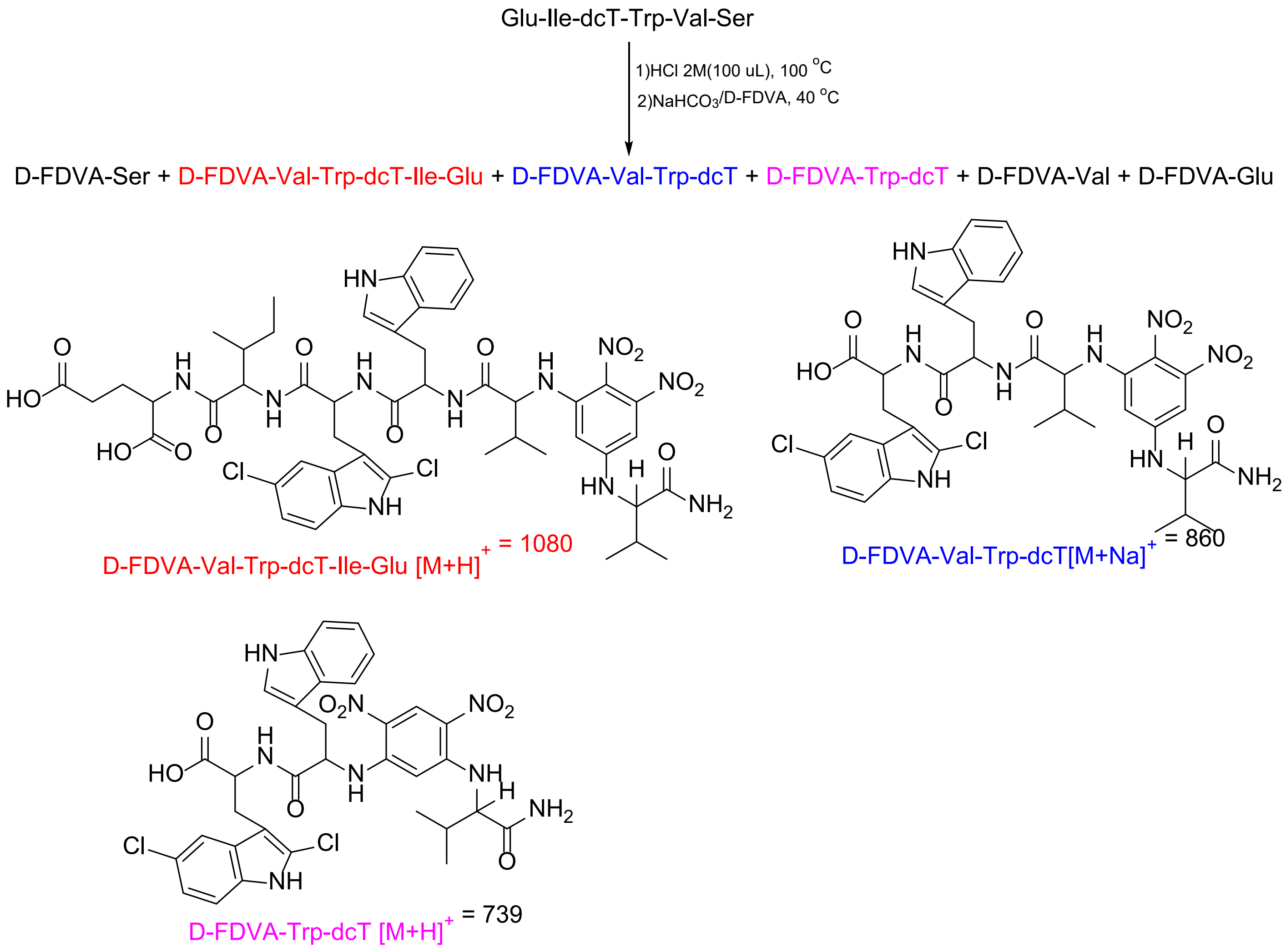

Figure S22. Partial degradation scheme of linear amycolatomycin A under acidic condition at 100 ºC

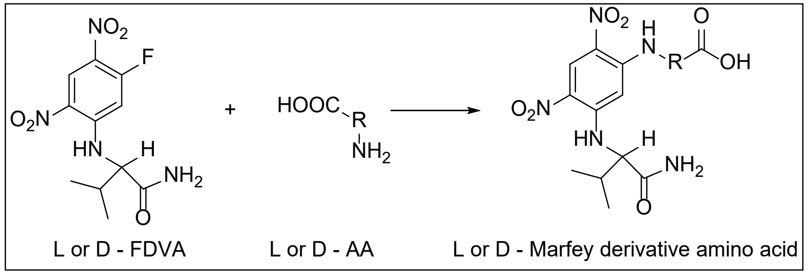

Figure 23. General reaction of Marfey's reagent and amino acid

Table 2. Retention time of L or D authentic amino acid derived D-FDVA

Nr.	Amino acid	Marfey's amino acid derivatives retention time (min)	Mass $[M+H]^+$
1.	L-Valine	8.9	398
2.	D-Valine	7.4	398
3.	L-Serine	5.9	386
4.	D-Serine	5.7	386
5.	D-Glutamic acid	6.1	428
6.	L-Isoleucine	9.7	412
7.	D-Isoleucine	8.0	412
8.	L-Tryptophan	9.0	485
9.	D-Tryptophan	8.1	485
10.	L-Aspartic acid	6.1	414

Table 3. Retention time of L or D authentic amino acid derived L-FDVA

Nr.	Amino acid	Marfey's amino acid derivatives retention time (min)	Mass $[M+H]^+$
1.	L-Valine	7.4	398
2.	D-Valine	8.9	398
3.	L-Serine	5.7	386
4.	D-Serine	5.9	386
5.	D-Glutamic acid	6.5	428
6.	L-Isoleucine	8.0	412
7.	D-Isoleucine	9.7	412
8.	L-Tryptophan	8.1	485
9.	D-Tryptophan	9.0	485
10.	L-Aspartic acid	5.8	414

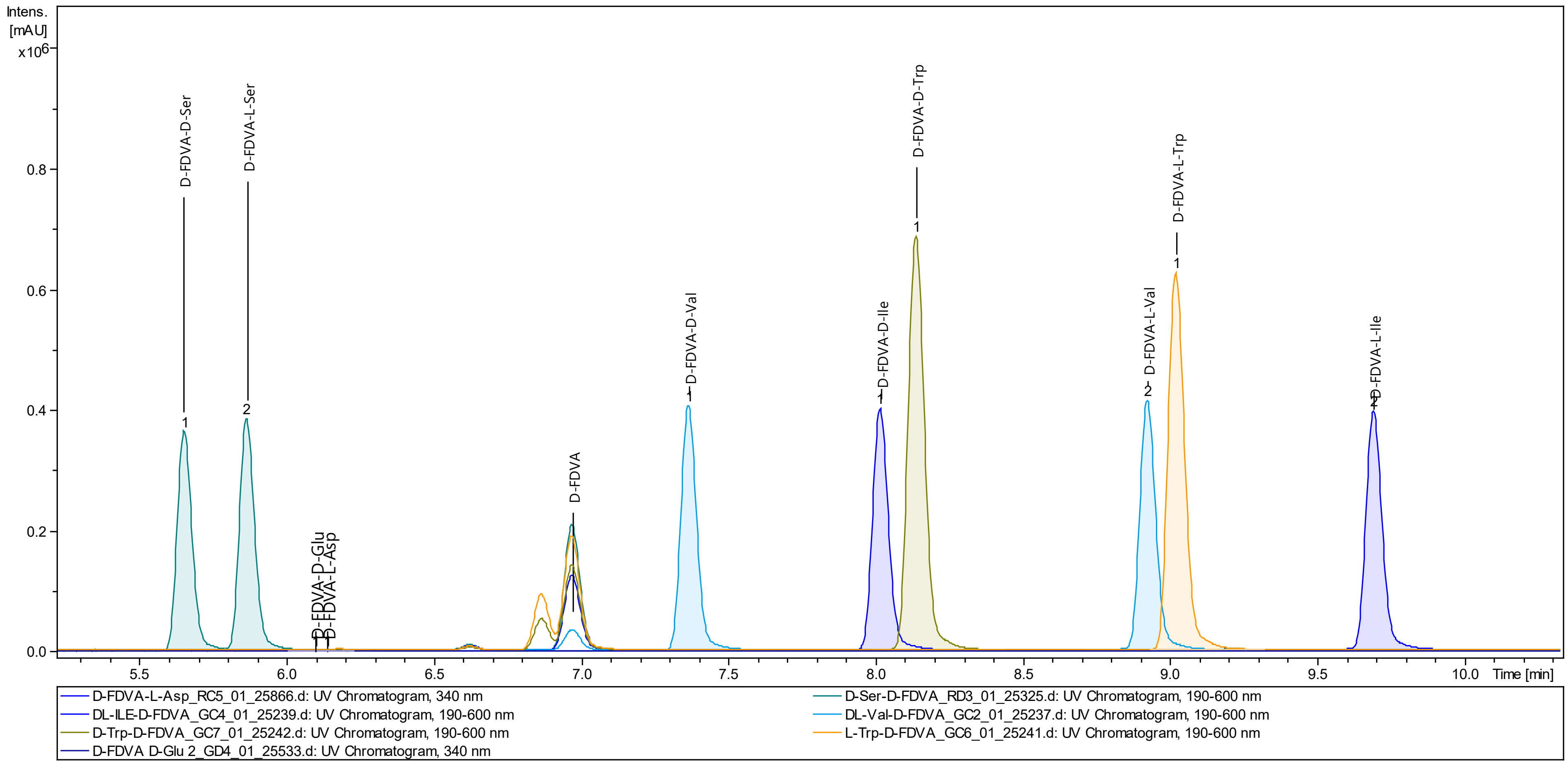

Figure S24. HPLC-DAD/MS chromatogram of L/D or DL authentic amino acid derived D-FDVA

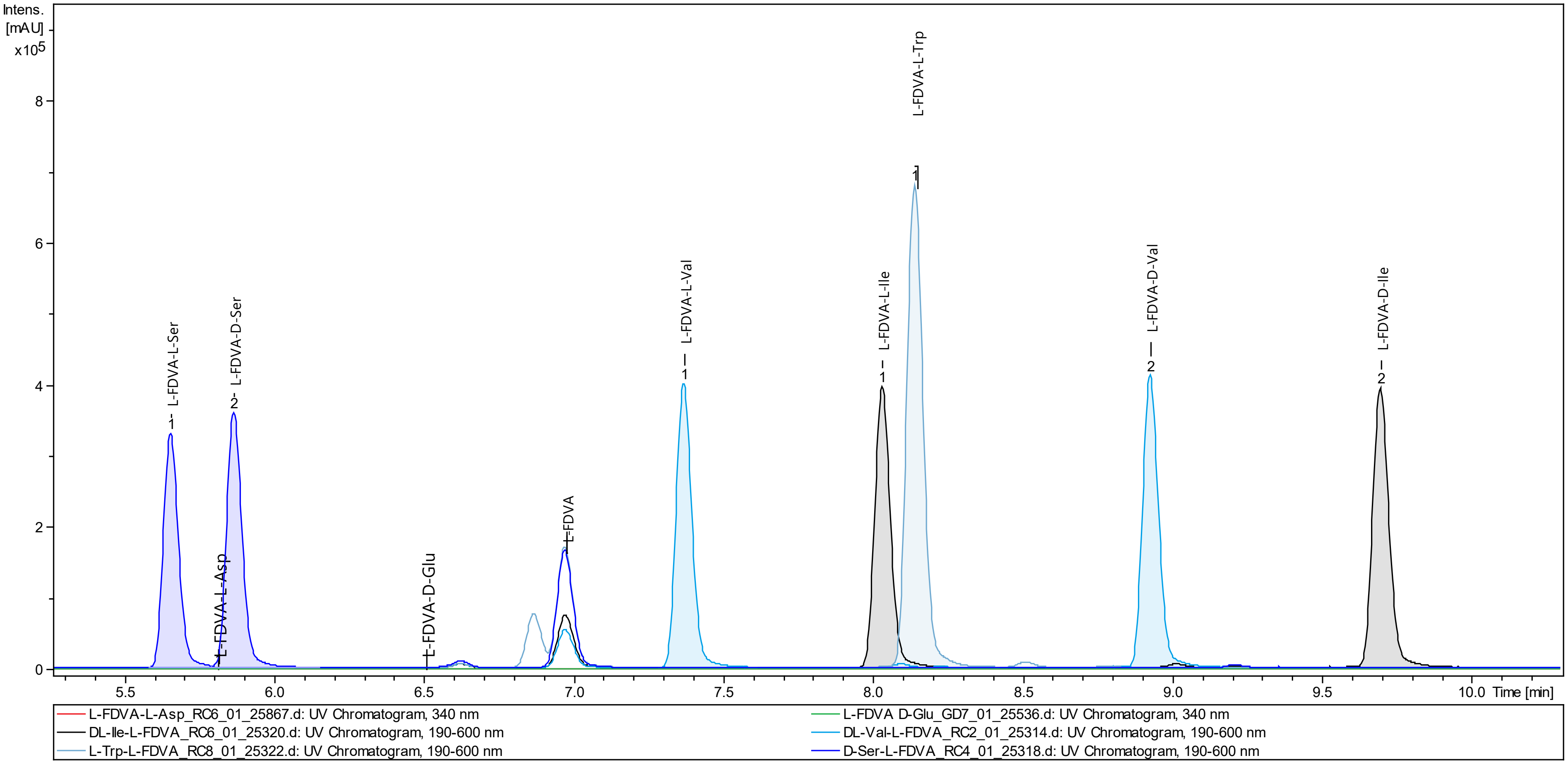

Figure S25. HPLC-DAD/MS chromatogram of L/D/DL authentic amino acid derived L-FDVA

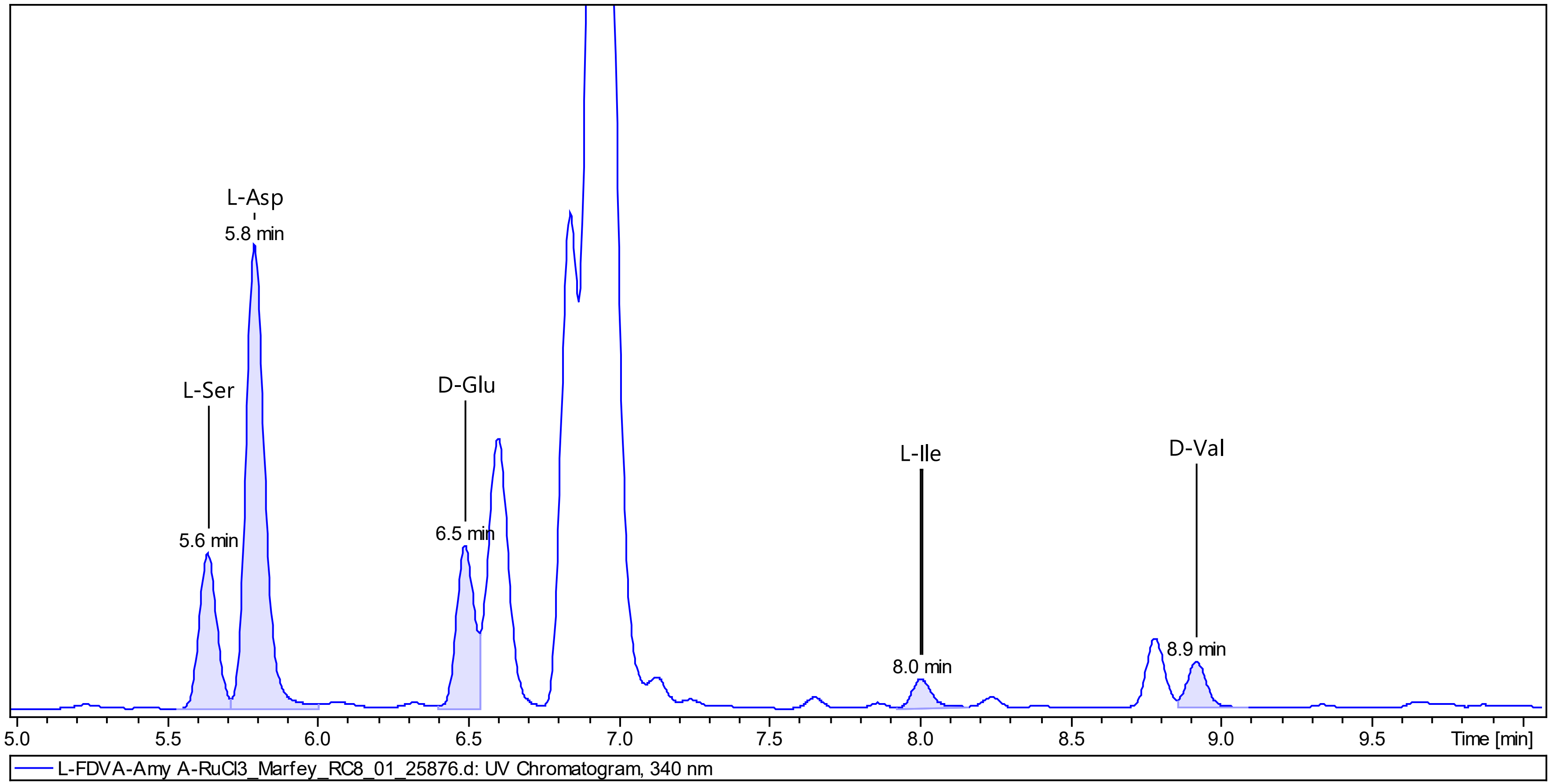

Figure S26. HPLC-DAD/MS of Amycolatomycin A catalyzed by $RuCl_3 \cdot H_2O$-$NaIO_4$ followed by hydrolysis and derivatization with L-FDVA on C_{18} column.

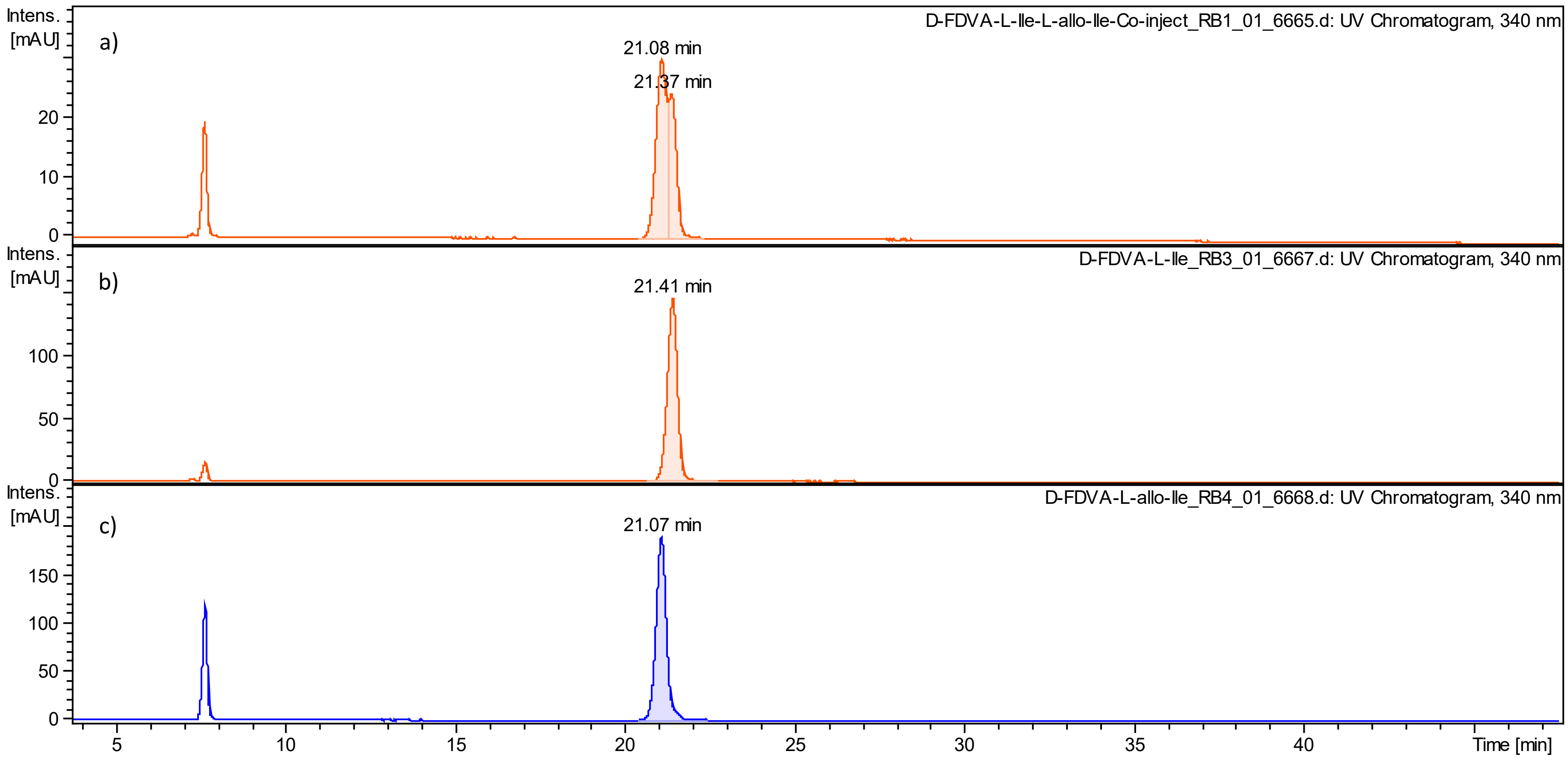

Figure S27. HPLC-DAD/MS of Marfey's analysis on C_4 column of an authentic amino acid L and L-*allo*-Ile: co-injection of D-FDVA-L-Ile-L-*allo*-Ile (a), D-FDVA-L-Ile (b), and D-FDVA-L-*allo*-Ile (c)

.

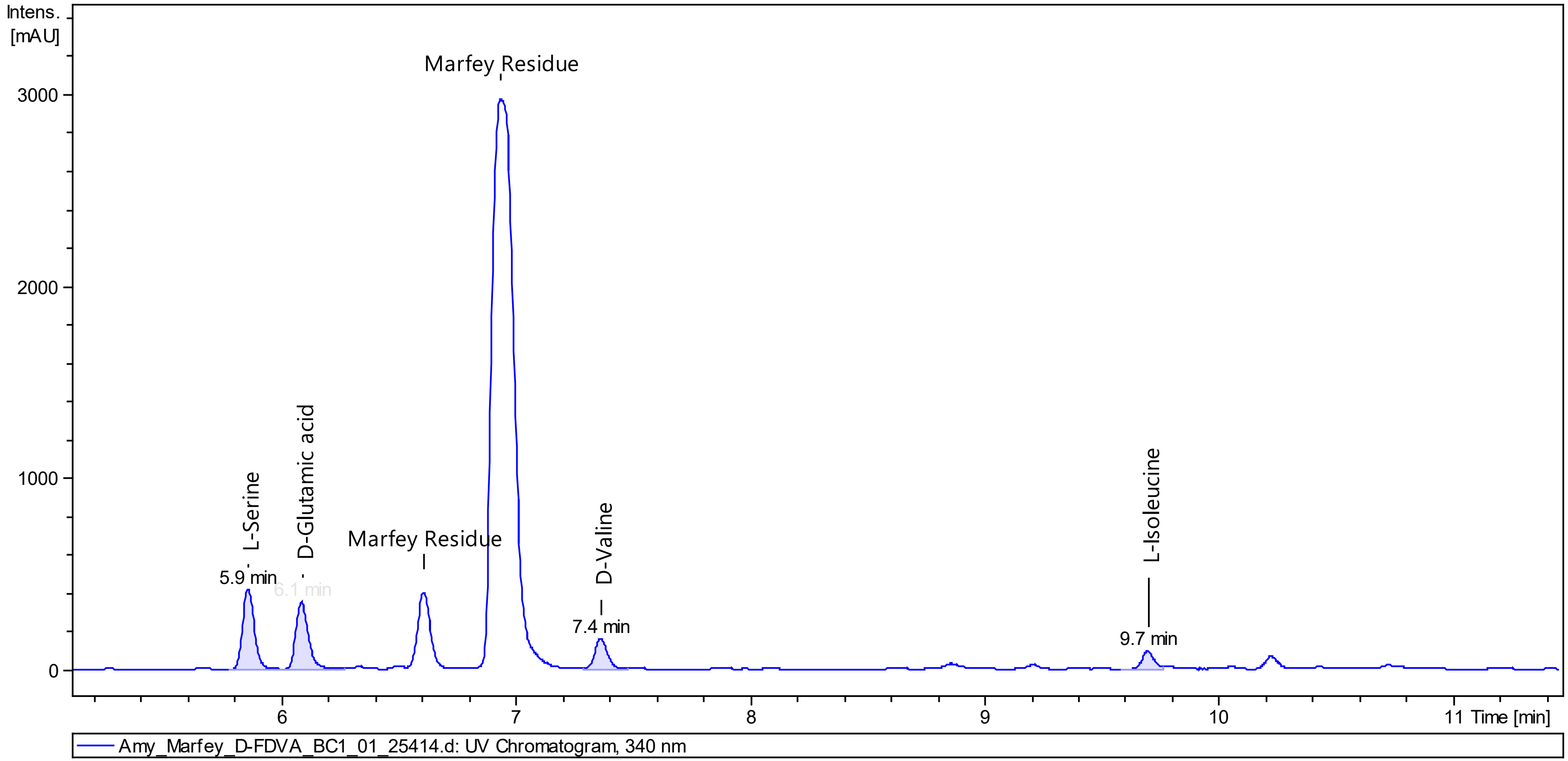

Figure S28. HPLC-DAD/MS of Marfey's analysis of amycolatomycin A derived D-FDVA in C_{18} column

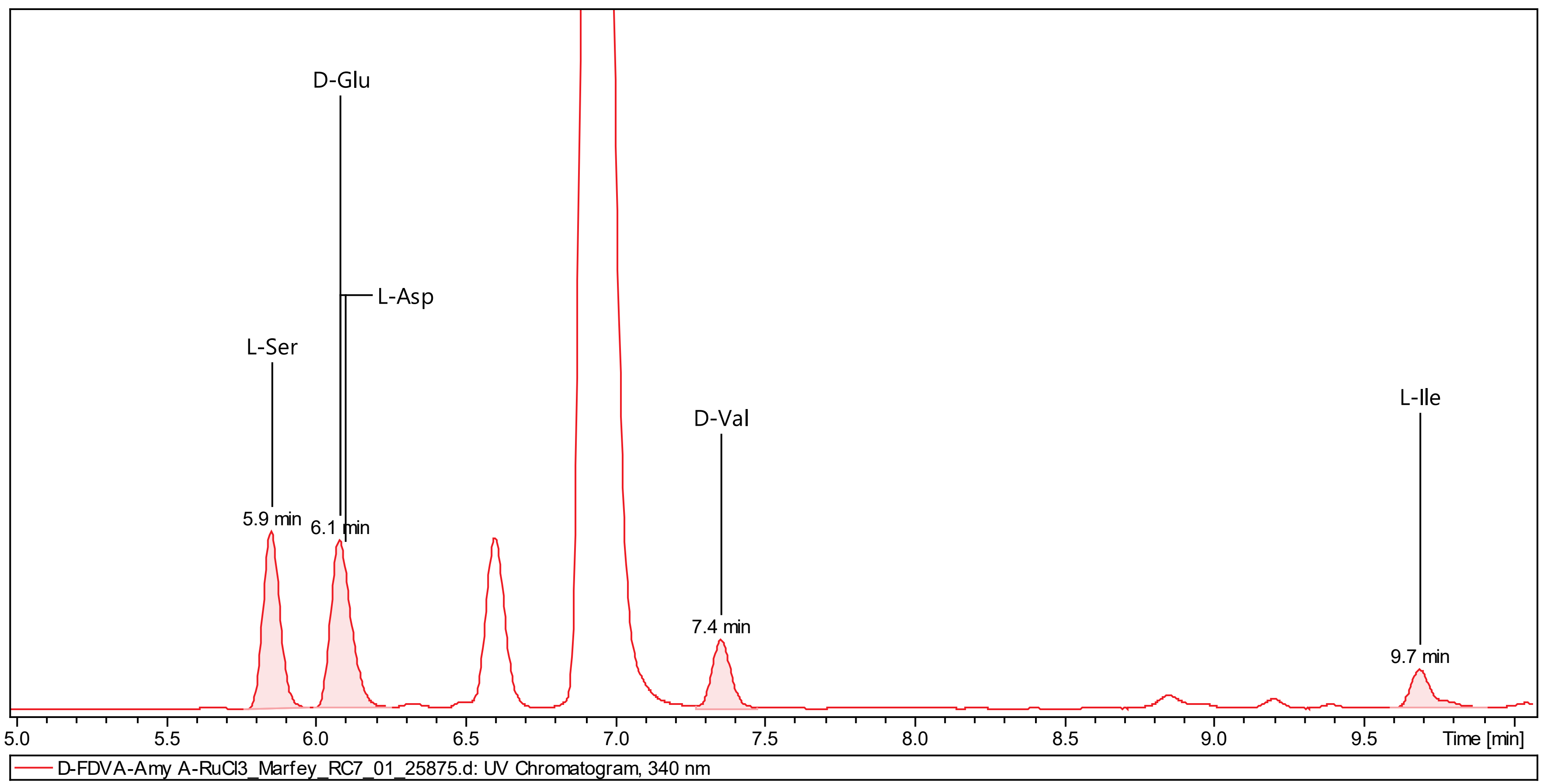

Figure S29. HPLC-DAD/MS of Amycolatomycin A catalyzed by $RuCl_3 \cdot H_2O$-$NaIO_4$ followed by hydrolysis and derivatization with D-FDVA on C_{18} column.

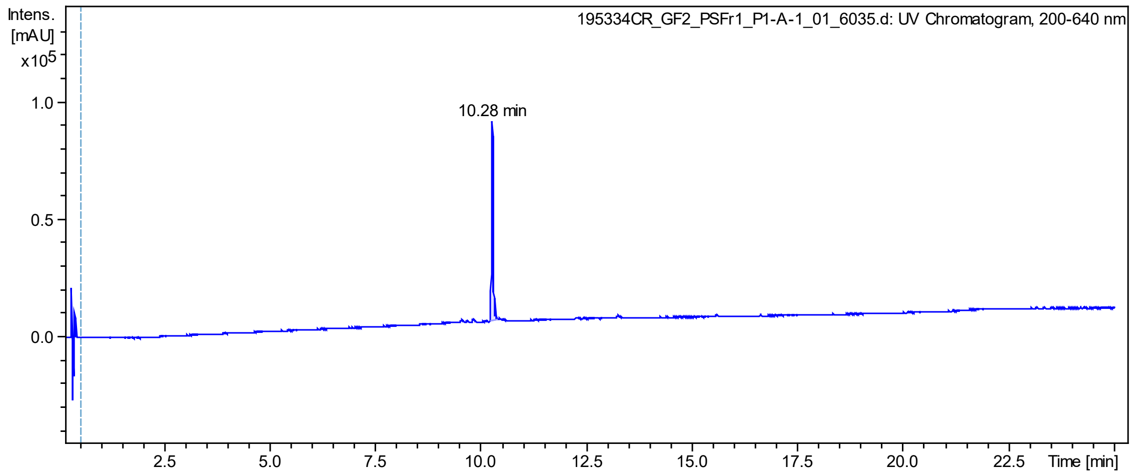

Figure S30. HPLC-DAD/MS chromatogram of amycolatomycin B

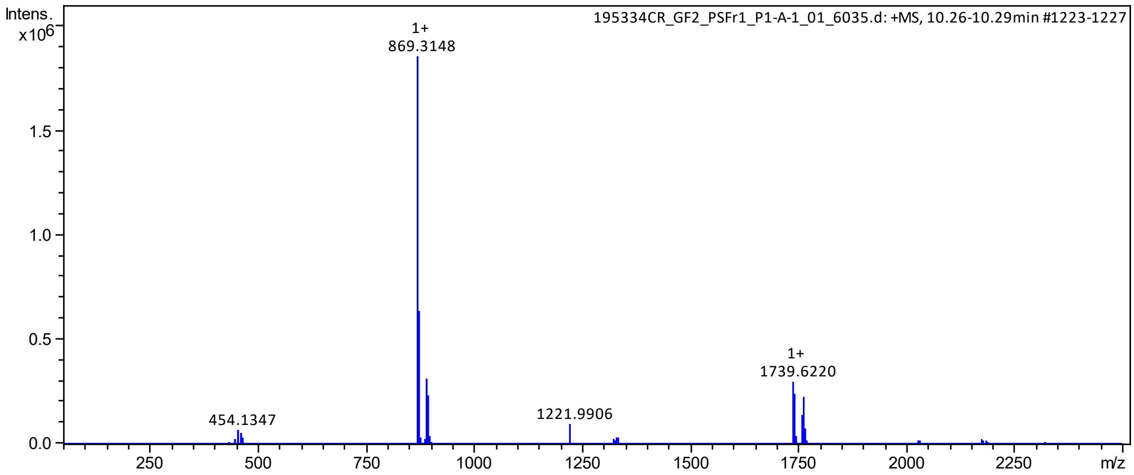

Figure S31. HR-ESIMS chromatogram of amycolatomycin B

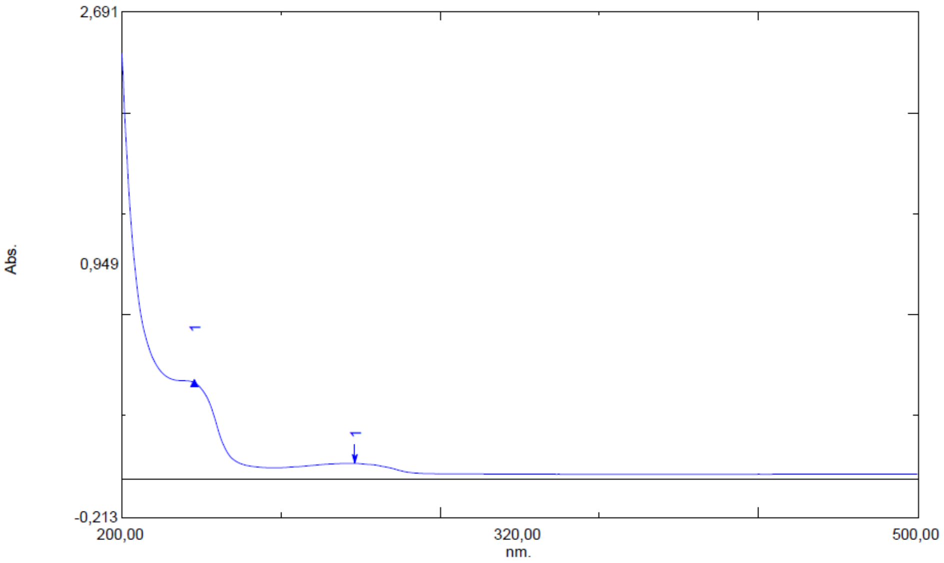

Figure S32. UV/vis spectrum of amycolatomycin B in MeOH (log ε) [neutral]λ_{max} 227 (3.90), 288 (4.70) nm.

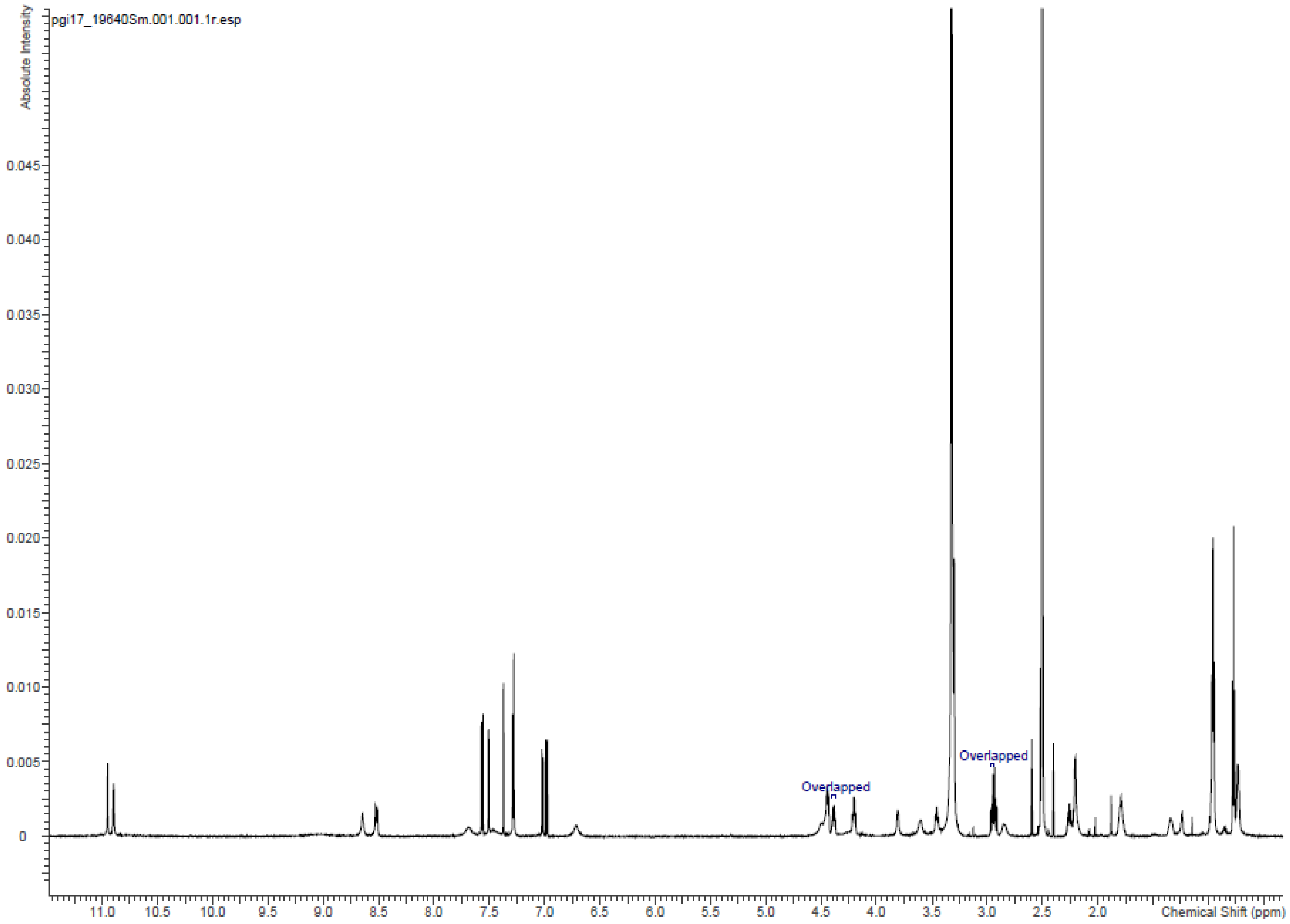

Figure S33. ^{1}H NMR spectrum of amycolatomycin B in DMSO-d_6 (700 MHz)

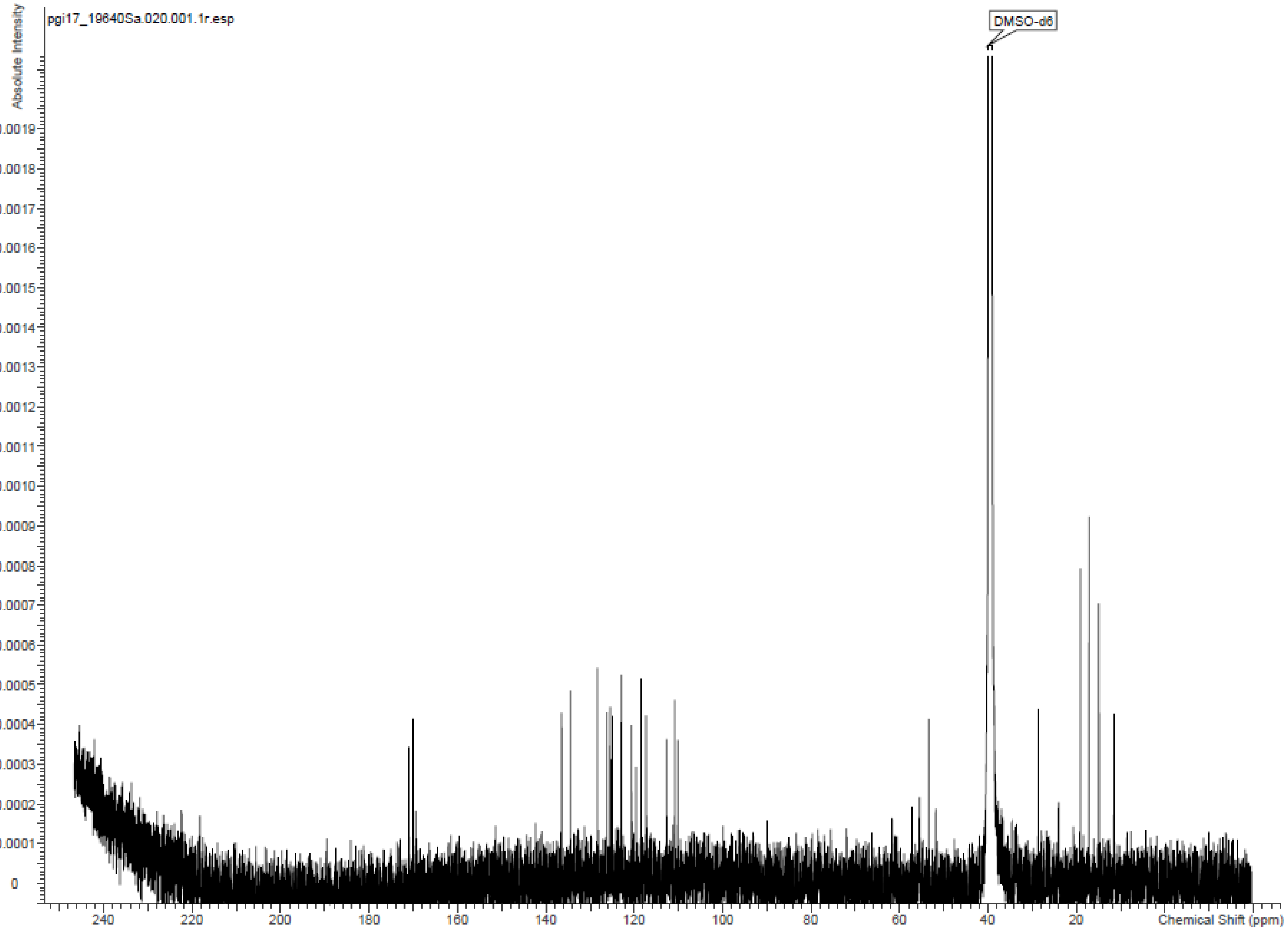

Figure S34. ^{13}C NMR spectrum of amycolatomycin B in DMSO-d_6 (176 MHz)

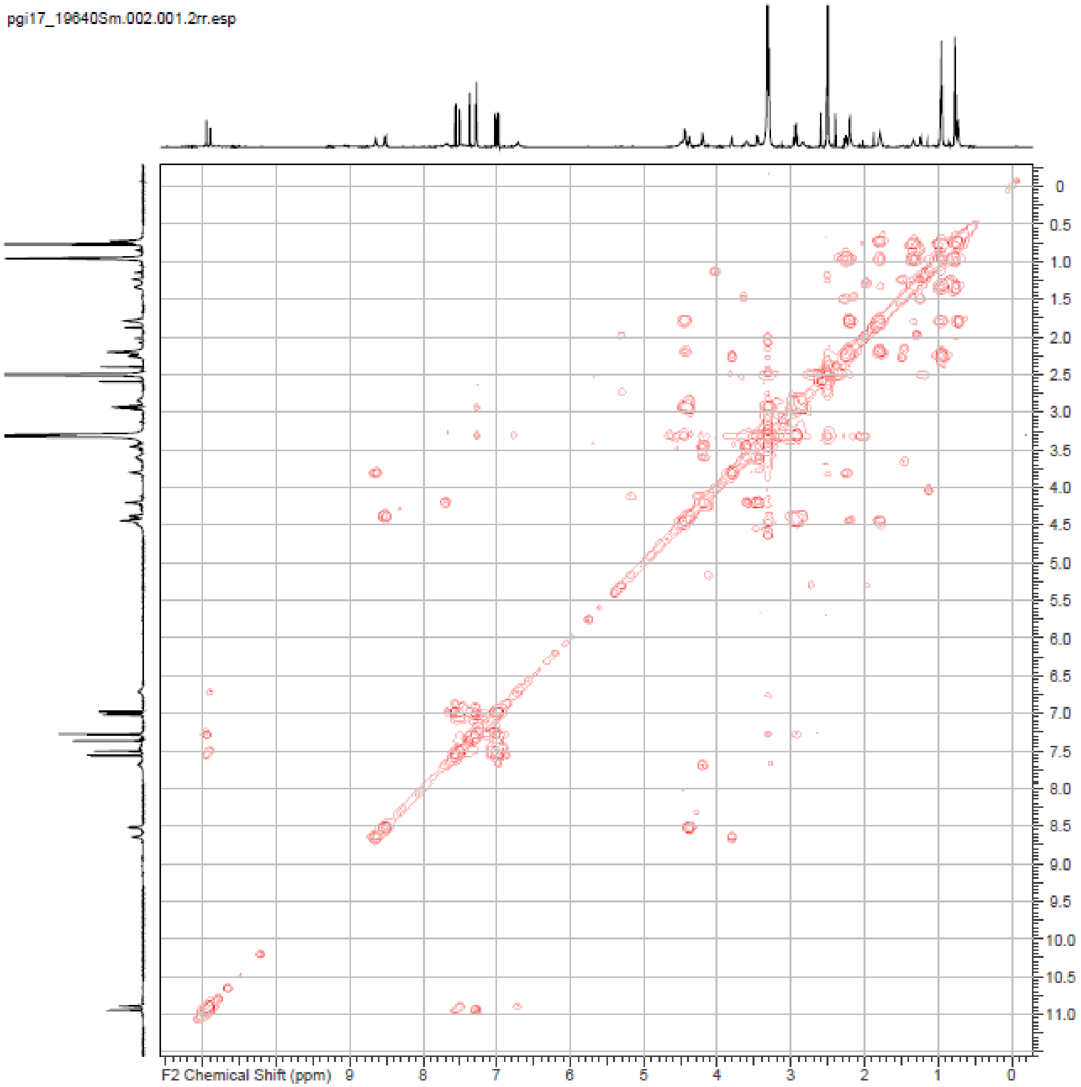

Figure S35. $^{1}H,^{1}H$ COSY NMR spectrum of amycolatomycin B in DMSO-d_6

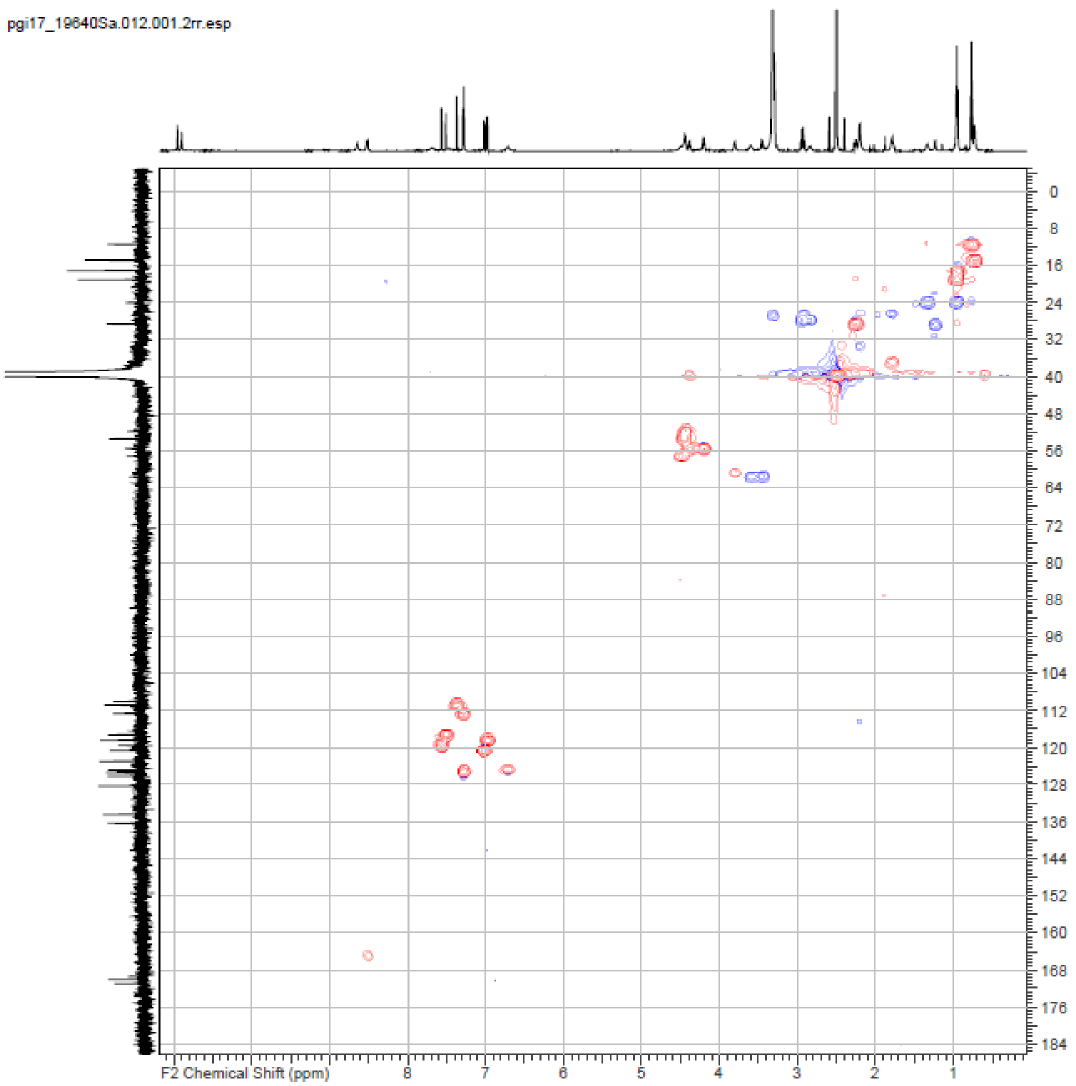

Figure S36. $^{1}H,^{13}C$ HSQC-DEPT NMR spectrum of amycolatomycin B in DMSO-d_6 (700 MHz, 176 MHz)

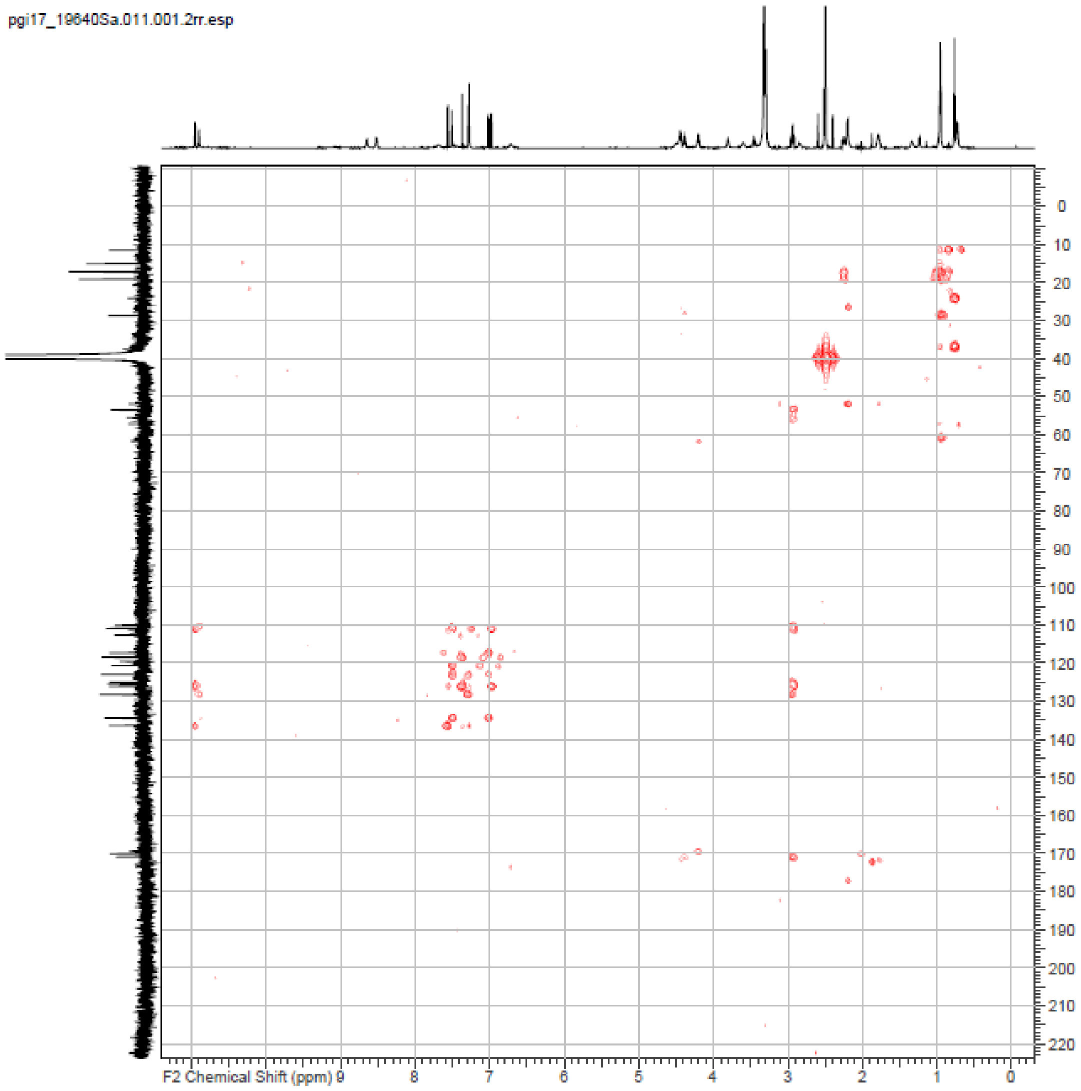

Figure S37. ^{1}H,^{13}C HMBC NMR spectrum of amycolatomycin B in DMSO-d_6 (700 MHz, 176 MHz)

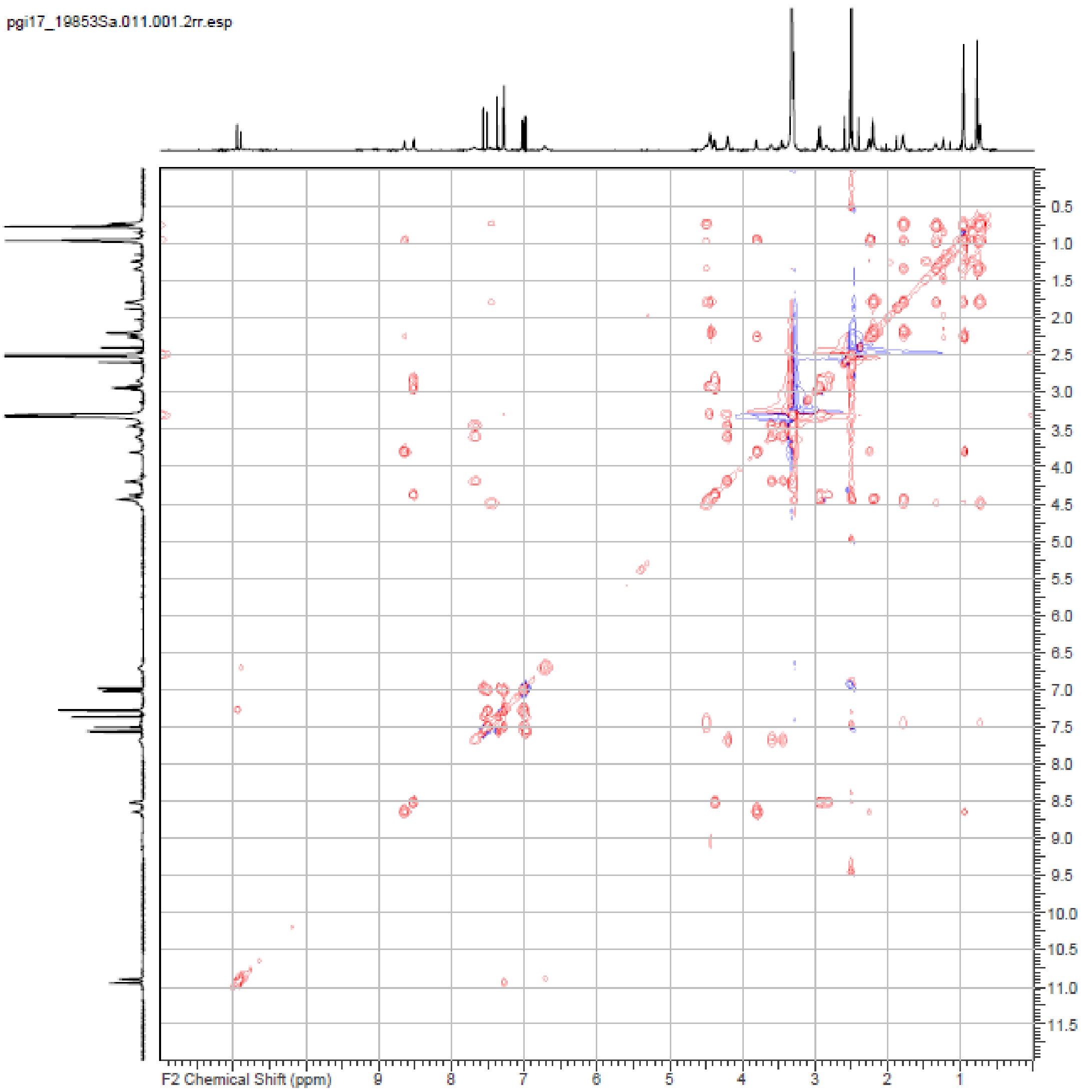

Figure S38. TOCSY NMR spectrum of amycolatomycin B in DMSO-d_6 (700 MHz)

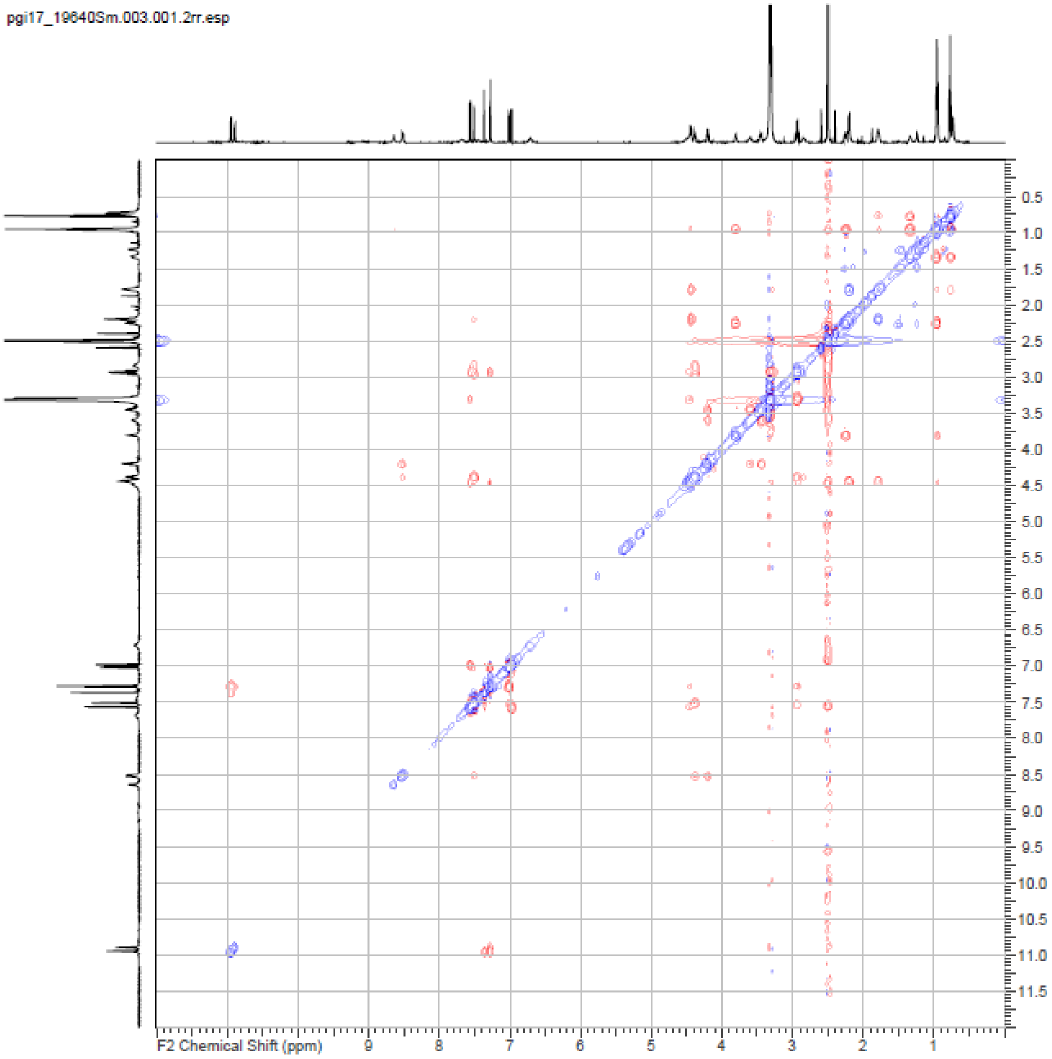

Figure S39. ROESY NMR spectrum of amycolatomycin B in DMSO-d_6 (700 MHz)

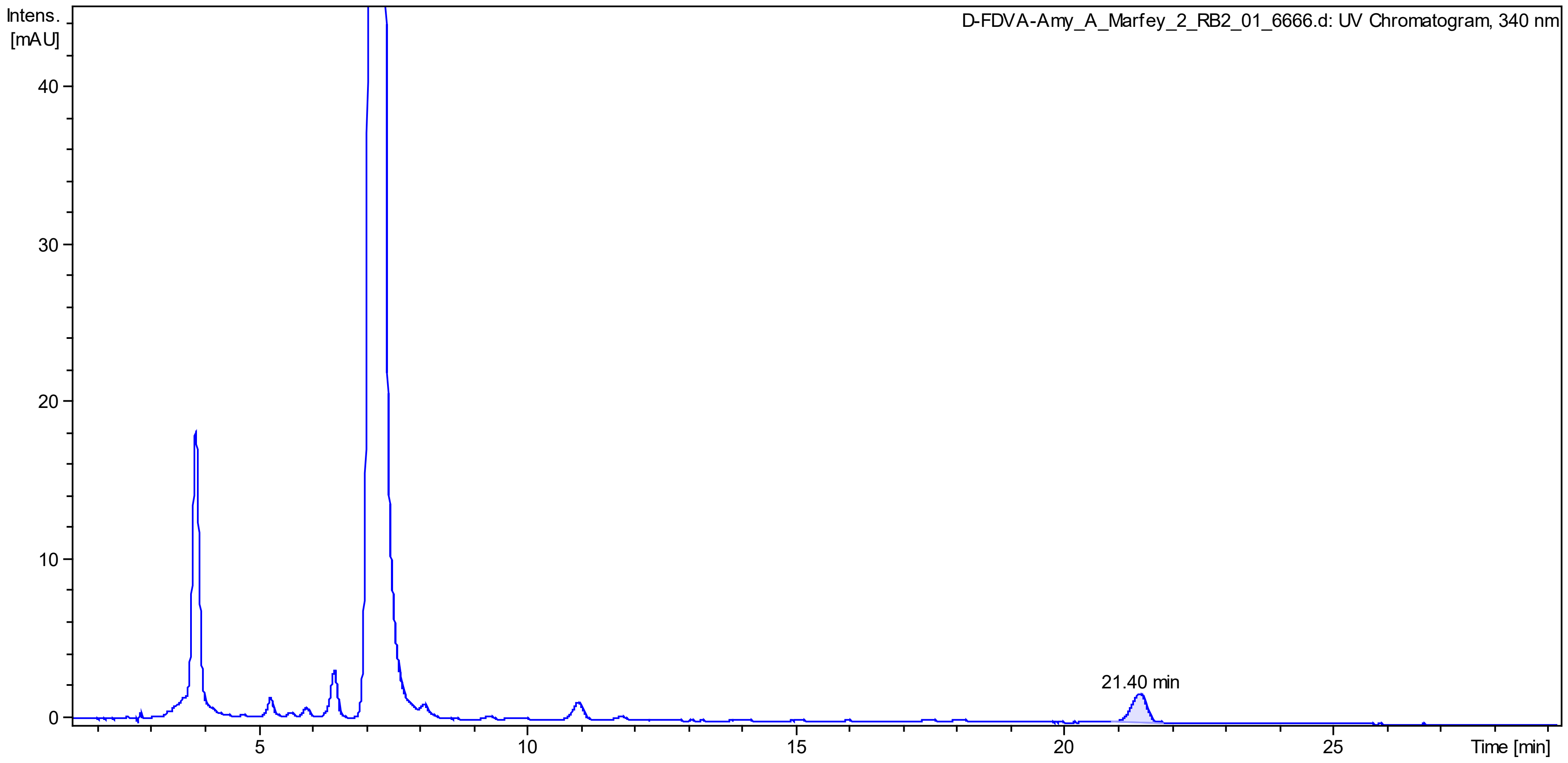

Figure S40. HPLC-DAD/MS of Marfey's analysis on C_4 column of amycolatomycin A derived D-FDVA

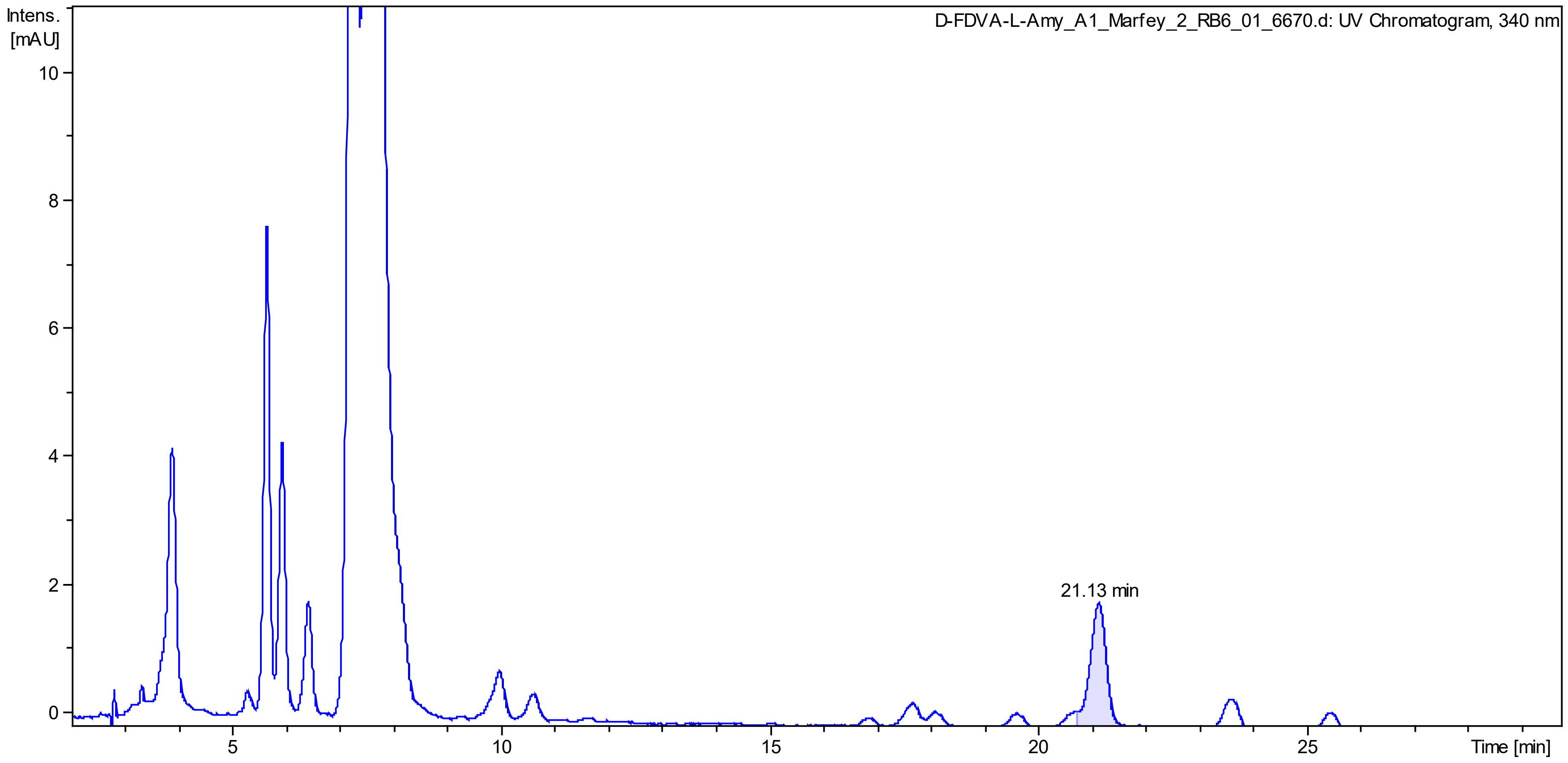

Figure S41. HPLC-DAD/MS of Marfey's analysis on C_4 column of amycolatomycin B derived D-FDVA

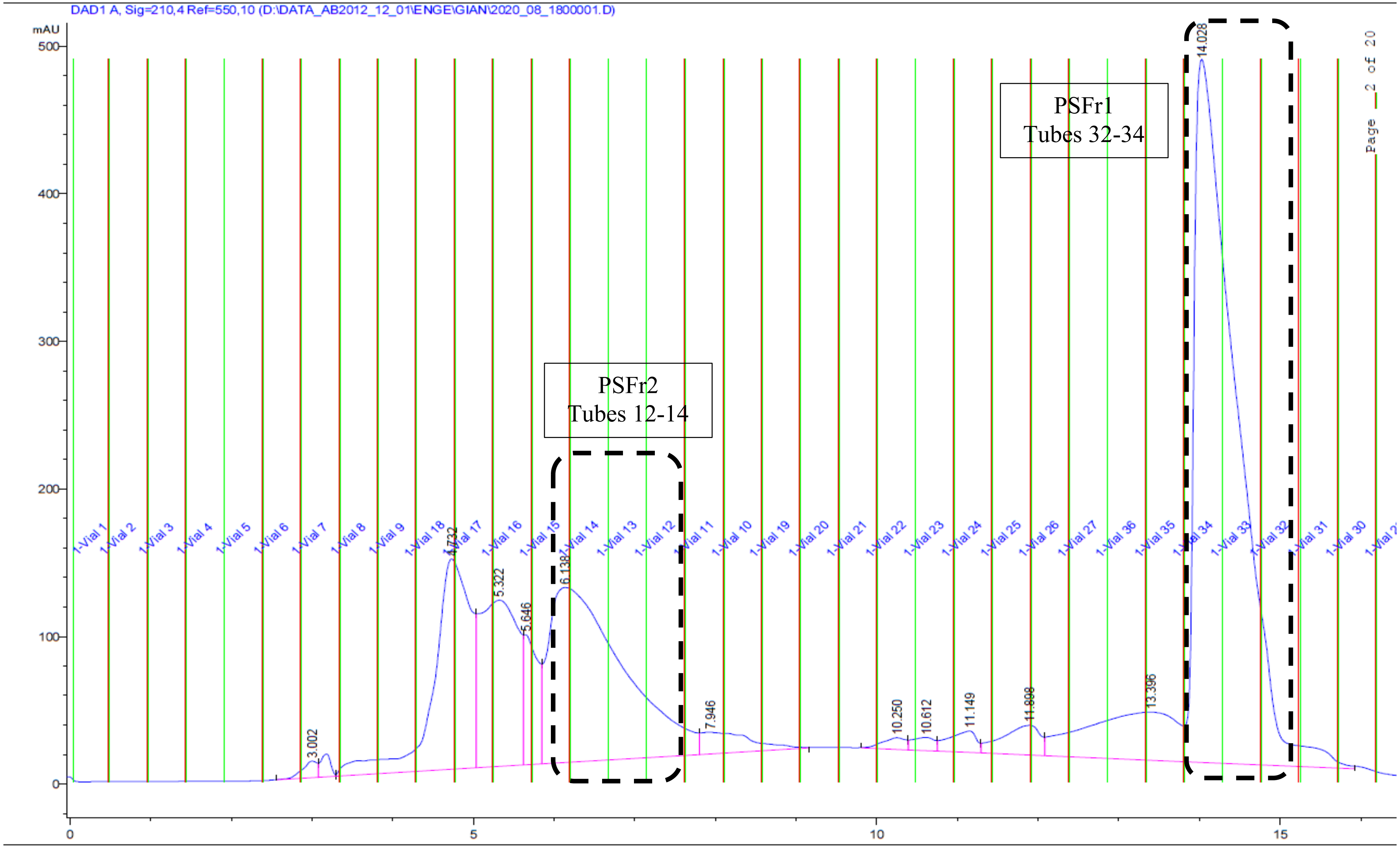

Figure S42. Isolation process chromatogram of GF2 fraction in preparative HPLC

Table S4. List of amycolatomycin biosynthetic genes and its nucleotide sequence (*ammA* – *ammD*)

Gene (nucleotide)	Locus Tag	Location
ammA (6279)	ctg2_2089	2,151,103 - 2,157,381
ammB (1290)	ctg2_2092	2,159,026 - 2,160,315
ammC (5865)	ctg2_2098	2,169,205 - 2,175,069
ammD (9192)	ctg2_2120	2,196,270 - 2,205,461

>ammA
ATGTCCCAGACTGCCAATGTGGAAACCTGGCTGCGGCGGCGTCGCGGGTCCGGTGGCGCC
GGCGGGATCGCCAGGGTCGAGCGCGGCGGGCCGCTGCCCATGTCGTTCGGCCAGGAACA
GATGTGGTTCCTCGACCAGCTGGTCCCCGACAGCGCCGAGTACCTGGTCCCGTTCGCCAT
CCGGCTGACCGGTGAGCTCGACCGGAAAGCGCTCTCGCGGGCGTGGGACGGGCTCGTCG
AGCGGCACGAGATCCTGCGCACGCGGCACGTGCTCGACGACGGGGAACCGCGTCAGCTC
ATCGACGAGCCGCGGTCCGGGCAGCTGTCGATGGTGTCGGTCGCCGGTGCCTCGGCGCAG
CAGCGCGTCACCGAACTGATCGAGGCGCAGTCCGCGCTGCCGTTCGACCTCGGGCGGCAG
TGGCCGGCGCGCGCGACGCTGGCGGAGGTCAGCGACACCGAGCACGTGCTGATCGTGGT
CTTCCACCACATCGCGTGCGACGCGTGGTCGTCGCGGCTGGCGCTGTCCGACCTGTGGAC
CCTCTACGCCGACGGCGAGGCGGCGAAGCTGCCGGAACTGACCGTCCAGTACGCGGACT
TCGCCGCGTGGCAACGCGCGAGGCTGACCTCCGACACCGGCGACAAGCAGCTCGCCTACT
GGGAGGAGGTGCTGGCCGGGGTCCCGCAGCTGGACCTGCCCACCGACCTGCCGCGCCCG
CCGGTGCGCGGGTACGCCGGGGCCGAGGTGGACTTCGAGCTGCCGCCCGAGGTCGGCGC
CGAGGTGGTGGCGCTGGCCCGTGCCGCGGGCACCACGCCGTTCGTGGTGCTGCTGACCGC
GTTCCAGTCGCTGCTCGCGCGCTACACCGGCAGCACCGACGTCCCGGTCGGCACCATCGT
CTCCGGGCGGACCCGGCCGGAACTCCAGCACCTGGTCGGCAACGGCATCAACACGGTGG
TCATCCGCACCCGGTGGGCCGGGCGGGAGAGTTTCCGCGAGCTGGTCGGCGGGGTCCGC
GAGACCGTGCTGGACGCCTTCGACCACAGCGCCGTGCCGTTCGCCAGGGTGGTGGACAA
GGTGCAACCGCGCCGCGACCCGTCGCGGACCCCGGTGTACCAGGCGGTGTTCTCCTGGCA
CGAAGCCGGTGGTCACGGCCCGGAACTGATCGCCGGGCTGAAGGTCGAGCCGATCCCCG
TCGGCGGTGGCGTCGCGCGGTGCGATCTGGAACTCCAGGTGGGCCAGACCGCCGAGGGC
GGGCTGAGCGCGAGGCTGATCTACGCCACCGAACTGTTCGAGGCCGCCACGATCGAACG
CATGGCCGCGCACCTGACGCGCCTGCTCACCGCCGCGGTGGCCGACCCCGACCAGCTGGT
GTCCGCGATCGAGATGCTCGACGACGCGGAACTGGCTTCGCTCACCAAGGCCGCGGAAC
CGATGCCCGCCACCGAAGACGTGTTGCGGCGCTTCGATCAGCAGGTCGCCCGCACCCCCG
ACGCCGTCGCGGTGCGCACCGACGACGGTGAGACGACGTTCGCCGGGTTGAACGCACGC
GTCAACGGGATCGCCGCGCAGCTGCGGGAAGCGGGCACGAGCCGGGGCGAGATCGTCGG
CATCCTGCTCGACCGCGGCGTCGACCTTTTGGCTTCCATGCTGGCTACCTGGCGCGTGGGC
GCGGCGTACCTGGCGATCGGCCCGGAACTGCCGCCGCTGCGCTGGGGTCAGCTGCTCACC
GACAGCTCGGCCAAAGTGCTGATCACCGGTGAAGACGTCAGCGAAGTCTTCGGCGGACG
GGTGCTGGTGCCGGACCACAGCGATTCGGTAGAGGGCGAATGGCCCGGCGTGTTCGACCT
CGACGCGCTGGCGTACGTCGTCTACACCTCGGGCTCGACCGGCCGCCCCAAGGGCGTCGC
GGTGACCCACCGCGGCCTGATCAACCACCTGGACTGGGTGGTCGACAGCTACATCGGCGA
CCGCACCGGTGGCGCGCCGCTGTTCTCCACGGTCGCCGGTGACGTGGTGGTGCCGACGTT
GTTCGCGCCGCTGCTGGCCGGTCAGCCGGTGCACATGTTCCCGCAGGACCTCGACCTGGC
GGACCTCGGCGCGCGGCTCGCCGCGGCCAAGCCGTTCGCCTTCGTCAAGCTCACCCCCGG
CCACCTCGAACTGCTGTCGCACCAGCTGACGCCCGCCGAGATCAACGGGCTGGCCGGTTC
CGTGGTGACCGGTGGCGACGTGCTGCTCGACCACGTGGCGAAGCAGTGGAACTCCTGGCT
GGGCGACGGACGGCTGGTCAACGAGTACGGGCCGACCGAGATCACCGTCGGCAACTCGA
CCTACCTGCCGGGCGAGGACTCGCGGCGCGAGGTGGTGCCGATCGGCAGCCCGATCCCG
CACACCAGCATGTACGTGCTCGACGAAGCGCTGCGCCCGGCGGCGGTCGGCGTGATCGG

CGAGGTCTGCGTCGGCGGGTCCGGCGTCGCGCTCGGTTACCTGGACCAGCCGGGGCAGAC
CGCGGACAAGTTCGTGCCGGACCCGTTCGGGGCGCCCGGTTCGCGGTTGTACCGCACCGG
CGACCTCGGGCGCGTGATGGCCGACGGCAACCTCGAGTTCGTCGGCCGCGCGGACGGTC
AGGTCAAGGTGCGCGGGTACCGGGTGGAACTCGGCGAGATCGAAGGGGTGCTCACCGGG
CACGCCGAGGTCGAGGAGTGCCGCGTGGTGCTGCGCGGCGAAGGCGACCAGCGTGATCT
CGTCGCCTACGTGGTCGGCGCCGCCGACACCGAGCGGCTGGGCACCTGGCTGGCCGGCAC
GCTGCCCGAGTACATGGTGCCGAAGCTCGTGCCGCTGGACCGGATCCCGTTGACCGCCAA
CGGAAAGCTCGACGTGTCCGCGCTGCCGGAATGGGCCGCGCCCGAGGTGGACTTCGTGCC
GCCGCGGACGCCCGTGGAGGAGCGGATCGCCGAGGTGTGGGCGGAGGCGCTGCGGGTCG
AACGCGTCGGCGTGCACGACGGCTTCTTCGACGTCGGCGGCGACTCGATCCGGGCGGTGG
CGCTGGTCGGCGCCCTGCGGCACGCCGGGTACGACCTGTCCGTGGCGGACGTGTTCAGCT
ACGGCACGGTCGCCAAGCTCGCCAAGCTGATCGAAGGCCGCGAGCAGGTCTCCGAATCG
GGTTCGGTCGCGCCGTTCTCGCTGATCAGCGAAGCGGACCGGGCGCTGGTGCCGGACCGC
GTCGAGGACGCGTACCCGTTGTCCCGCAACCAGACCGGCATGATCTTCGAGATGCTCGCC
GACGAGCAGAACCCGTACCACTGCACCACCACCTTCTTCATCAAGGACCCGGCGCCGTTC
TCGCTGGCCGCGATGACGCAGTCGGCCCGGCTGCTCAGCCAGCGGCACGAGGTGCTGCG
GACCTCGCTGCACCTGTCCGGGTACTCGGTGCCGATGCAGCTGGTGCACGACGACGCGCA
GATGGCGGTCGGCATGCGCGACCTGCGGGGGATGGCCCCCGACGAGGTCGACCAGGTGC
TCAAGGAGCACGTGGCGCACGAGCGGGCCAACCTGTTCGACATGACCGTGCCCGGCCTG
ATGCGGTTCTTCGCCTTCCCCACCGACGAGAACGGGTACTGGCTCTCCATCACCGAATGC
CACCCGGTGCAGGAGGGCTGGGGGTACCACTCGATGGTGATGGAGCTGCTCACCTGCTAC
GGCTCGCTGCGCAGGGGTGAGGAACCCGTGCCGTACCACCGGCCGGAGGTCCGCTTCGCC
GACTTCATCGCCGAGGAGCTGGCCGCCATCGAATCGCCGGAGCACTCCGGTTACTGGCGC
GACCTGATCACCGGGCACACGCCGTTCGCGCTGCCCGACGAATGGGGCGACCGCGCGGC
ACCCGGCGCCAAGCACCAGGCGATGGCGGACTGGCGCGATCTCAAGCCGCGGCTGCGCG
AGGCGGCGGCCGCCGCCGGGGTGTCGATGAAGTGCGTGATGGTGGCCGCGTTCACCAAG
GTGCTGGCCCAGCTGACCGACGAGGAGTCGTTCCACTTCGGACTGATCACCGACGCCCGC
CCGGAACGGCTCGGCGCGGACACGGTGTACGGCATGTACCTGAACACCCTGCCGTTCGGC
GCGCGGCGCCCGGCCGGGACCTGGCGGGACCTGCTGCGCGCCACCTTCGAGCGCGAGGT
CGGGCTGTGGCCGCACCGGCACTTCCCGTACCCGGAGATCGTGCGGATGGCCGACAAGC
GGCAGCGGCTGATCGACGTGATGTTCAACTACCACGACTTCAACCAGGTGGACACCGATC
TGGTGGCCGAGCGGAAGGGGCTGGACGACAGCCCGACCGACTTCGGCCTGACCGTGTCC
ACCCGGGTGGACCTGGTGCTGGTGACCGCCGACTACCGCAAGCTCGGGCCGGCGCAGGC
CGAGCTGATCGCCCAGACCTTCCGGCTGGTGCTGGAGGGCATCGCGAACGACCTCGACGG
GGACGCCACCGCGACGCTGCTGCCGGAGGGGCACCGCGCCTGGCTGCTCGACTCCGCCA
GGGAACCGGCGCGGCCCGCCGGGCGGGCGAACGCGGTTTCCCACGTGCTGGCCGCGTTC
GGCGAGCAGGTGGCACGCACGCCGGACGCCGTCGCGGTGCGCTGCGGTGACGAGGTGCT
CACCTACGCCGAACTCGACGAGCGGGCCGCCCGGCTGGCGCACCACCTGCGGGCACGGG
GGTTCACCCCGGGCTCGGTGGCCGGGGTGATCGGGCGGCGGGACCTGAACCTGTTGCCCA
CGCTGCTGGCCACCTGGAAGGCGGGCGGGGCGTACGTGCCGCTCGACCCGGCCGCCCCG
GAGGACCGGCTGGCCTACGTGCTGGCCGATTCCGGTGCCCAGTGCGTGCTGACCGCGCCC
GGGCTGACCAGCCGGGTCCCGGCCGCGTTCACCGGTCCGACGGTGTTCACCGACGCGGTG
GGCGAAGCGCGGCTCGACGACCCGGGCACCGGGGACGGGCTCGCCTACGTCATCTACAC
CTCCGGTTCCACCGGACGGCCGAAGGGCGTGCGGGTCAGCCACCGCAACCTGGCCAACT
ACCTGGGCTGGGCGGCGGCCGACTACCCGGCGGGCGGGGACGGCGGGGCGCCGCTGCTG
TCCACCATCGCCTCCGACCTCCCGGTGACCGCGTTGTTCGTGCCGTTGCTGGTCGGCCAGG
CGGTGCACGTGCTGCCCGACGACCTCGACCTGACCCACCTCGGCGCCGCGCTGCTCGCGG
GCGGGCCGTACAGCTTCGTCAAACTGACCCCCGGTCACCTGGAGGTGCTCGCGCAGCTGA
TCGACCAGCCGGTGGCCGCCACCATGGTGGTCGGCGGCGAGGCGTTGCGGGGCGCGGTC
GCCCAGCGGTGGGCGTCGATGCCCGGCAGCCGGGTGATCAACGAGTACGGGCTCACCGA
GACCACCGTCGGCAACTCGGTGCAGGTGTACGGCGACGGCTCGCGGCCGGTGGTGCCGA
TCGGCAAGCCGGTGCCGAACTCCGCGATGTACGTGCTGGACAACCGGCTCGAACTGGTAC
CGGCCGGGGTGGTCGGCGAGGTGTGCGTGGCCGGCGACGCGGTGGCGATGGGTTACGTG
AACCGCCCGGCGATGACCGCGGAACGGTTCGTGCCCGACCCGTACGGCCCGCCGGGGTC
ACGCCTGTACCGCACCGGCGACCTCGGCCGGGTGCTCGCGGACGGTTCGGTCGACCTGGT
CGGGCGCCGCGACGGGCAGGTGAAGATCCGTGGGTACCGAGTGGAACTCGGTGAGATCG

AAGCCGTGCTCGGCGACCACCCGCGGATTTCCGAGGCCAGGGTGCTGTTGCAGCCGACCG
CGCGCGGGGACCGCAAACTGGTGGCCTACCTCGTCACCGACGGTGAACCGCTCGGGCTG
GAGGAGGTCCGCTCGTGGCTGGCCGATCGCCTGCCCGACTACATGCAGCCCTACGCGGTG
GTGCCGCTGGAGCGAATGCCGTTGACCGCCAACGGAAAGCTCGACACGGCGGCGCTCCC
ACTGCCGGACGGCGGCGGGGCCGAGGACAGCTATGTGGCGCCGGTGGGCCCGCTCCAGG
AACAGCTCGCCGCGGTCTGGGGCGAGGTGCTCGGGCGCCGGGTCGGCGCGGAGGACACC
TTCGCCGAACTCGGCGGCGACTCGATCAGCGCGGTGGCGCTGACCGGCGCGCTGGACAA
CGCCGGCCTGGGCGTCACCGTGCGGGAGTTGTTCGCCCACCCGACGGTGGCCGGGCTGGC
GGGTTTGCTGGCCGCGCGTTCCGGCAGCTGA

>ammB
ATGGCCTCCTACCTGGCCAGGGCGGGCCTGTCGGTGGTGGTCTTCGAGAGCGAGACCTTC
CCGCGCCCCCACGTCGGTGAGTCCCTGGTCCCGGCGACCACCCCGGTGCTGGTCGAGATC
GGCGCGCTCGACAAGATCGACGAGGCCAAGTTCCCGCGCAAGTACGGGGCTTCCTGGAC
CTCCGCCGAGTCGAGGTCCATCCCGACGATGGGCTACGAGGGGCTCAGCCACGACTGGTC
GGCCGAAGTCCAGTTCGTCGAGCGCAACCAGGCCGGCGTCGACCGCGACTACACCTTCCA
CGTCGACCGCGGCCGGTTCGACGCCATCCTCCTGCGGCACGCGCAGGAGCAGGGCGCGA
CCGTGTTCAACGGCGCGCGCGTGCTCGACGTCGACTTCGACGACCCGGACGTGGTCCGGC
TGAGCGTCCGGTTCGGCCCGCGCACCACCGACTACACCGTGCGCATGGTGGTCGACGCCT
CCGGGCGGCAGACCCTGCTGGGCCGCAAGCTCAAGGTGAAGGTCGCCGACCCGGTGTTC
GACCAGTACGCGCTGCACACCTGGTTCGAGGGCCTCGACCGCGAGGCGCTCAGCCCGAA
CGTGGCCACCGCGGACCACATCTTCGTGCACTTCCTGCCGATCAAGGACACCTGGGTCTG
GCAGATCCCGATCACCGACACCATCACCAGCATCGGCGTGGTGACGCAGAAGAAGCGGT
TCGCCGAGGCGGCCACCGACCGCGAGCAGTTCTTCTGGGACTTCGTCTCCAGCCGTCCCG
AGCTGGCCGAGGCGCTGAAGAAGGCCGAGCAGATCCGGCCGTTCAAGGCCGAGGGCGAC
TACAGCTACGCGATGGAGCAGATCTGCGGCGACCGGTTCGTGATGATCGGCGACGCGGC
GCGGTTCGTCGACCCGATCTTCTCCAGCGGGGTGAGCGTCGCGCTCAACAGCGCGCGGAT
CGCCAGCCAGGACATCATCGCCGCGCACGCGGCCGGTGACTTCGACAAGAAGCGGTTCG
ACGCCTACGAGACCAAACTGCGCCGCGCGGTGAAGTACTGGTACGAGTTCATCTCCATCT
ACTACCGGCTCAACGTGCTGTTCACCGCGTTCGTCCAGGACCCGCGGTACCGCATCGACG
TGCTGAAGATGCTGCAGGGCGACGTGTACGACGGGACCGAGCCCAAGGCGCTGGCCGCC
ATGCGCGAGGTGGTGGCCGCGGTGGAGAACGACCCCGACCACCTGTGGCACCCGTACCT
CGGCTCCCTGCGCGCGCCGACGTCGGCCCCGAGCTTCTGA

>ammC
ATGGTCACGGGGAACCACGGACGACTGCCGGTGTCCAGTGCGCAGCGAGCCGTCTGGTT
GGCCCACCAGCAGGATCCGACCGAACGCCGGTACACCTGCGCGGAATACCTGACCGTCA
CCGGACCGCTCGACCTGCCGCTGTTCCGGCGGGCGTGGGCCGCGCTCGGTGCGGAAGCCG
ACGTGCTGCGGGTGCTGCGCGTCGAGGAAACCGCCGACGGTGAGCTGAACCAGGTGCTC
GCCGAGTGCGTCCCGCCGCTGCACGAACTGGACCTGCCCGACGACACCGCAGCGCTCGAC
TGGATGCACCACGACCTCGACCAGCCGATCGATCTCGCGACCGGACCGGTGTCCCGGGCC
GCGCTGCTGCGGATCACCGGCCACCGGCACCTGCTCTACCTGCGCATGCACCACATGTTC
ACCGACGGGCACAGCATGCACCTGATGCAGAACGCGCTCGCCGACACCTACACCGCCCT
GGTGCGCGGGCAGGACCGGACCACCGCCCGCCTCGGCGGGCTCGACGCACTGATCGCGA
AGGACGAGAAGTACCGGGCCAGCGCGGACTTCGAGGCCGACCGCGCCTACTGGACCAAG
CGCTTCGCCGACTCCCCCGAGCCAATGCGGGTGCCCGCCGTGCTGGGTGACCGGGACGAG
CGCACGATCCGCGCGCGGCAGGTGTCCTACCTGCCCGCCGGGGAATCCGCGCCGCTGGCC
GAGGCCGCCGCCGGGCTCGGCACCACCTGGCAGCTGGCGGTGGTCGCGGCGACCGCGGC
CTACCTGCACCGGATCACCGGCCGCCGGGACGTGGTGCTCGGGCTGGCGGGCAACGGGC
GGCGCGGCGGGCGCGCGAGCCTCACCCCGGGCATGGCCGCCAACACCCTCGCCCTCCGG
CTGGAGGTGTCGCCGTCGATGACCCTGCGGCAACTGGTGCCGCTGGTCGCCGAGGAGACC
GCCGCCGTGCAGCGGCACGAGCGCTTCCGCTACGACGACCTGTGCCACGCGCTCGGCACG
CGGCTCGCCGAGGACGGGCCGCTCGGCCCGATCCTCAACTTCATGCCGTACGCCAGGGAG
TTCCGCTTCGCCGACGCGACGGCGAGCGCGGTCAACCTGGCGTCGGGGCCGAGCATCGAC
CTCACCTTCGGCGTGACCGGCGCGGCCGAGCAGGGCCTGGCACTGGCCGTCGACGCCGAT
CCCGCGCTGCACCGGGCCGATGGGCTCGCGGGGGCGCTGCGCCGGTGGACCGCCTTCGTG

CTGGCCGCCACCGCCGCGCCCGACCAGGAGTTCCGGTCGATCGAGCTGCTCACCCAAGCC
GAACGCGCCGAAGTGCTCGTCCGGCGCAACGACACCGCCCGCGCCCGCGACGGGCGCAC
CGTGGTCGACCTCGTGCGCGAAGTCGCCCGGCGGCAACCCGACGCGATCGCGCTCAGCG
GCACCGGGAGCCAGTTGACCTACGGCGAGCTGGAGGCGAAGGCGGGCCTGCTCACCGCC
CGGCTGATCGAGCGCGGTGTGCACCGGGAGGACGTCGTCGGGCTCGCCATGCCGCGCTCC
CCCGAGCTGATCGTCGCGATGCTCGCCGTGCTCCGCGCCGGCGCGTCCTACCTGCCGCTC
GACCTCGCCTACCCGCCGGATCGCCTGCGGTACATGGTGACCGACAGCGCGCCGCGGTGC
GTGCTGGTCACCGACAGCGCCACCGCGGCCGCCGTGCTGCCCGCCGACGTGGTCATGGTC
GACGCCGGCGAACTGCCGTCCGGCGGGCACCCCGCCGAGCCGGTCGCGCTCGCCCCCGCT
TCGGCGGCCAACGTCATCTACACCTCGGGGTCCACCGGTTCGCCGAAGGGCGTGGTCGCA
CAGCACCAGGGCCTGGTCAACTTCGCGCTCGACCACGTGGGCCGGTTCGGCATCACCCCG
GACAGCCGGGTGCTGCAGTACCTCTCCCCCGCCTTCGACGCCGCGGGCGAGGACATCTGG
CCTGCACTGGTCGCCGGGGCCCGGCTGGTGCTGCCGCCCGAGCCCGCGAGCATCACCCCC
GCCGAGCTGATCACCACGCTGAAGTCCGAGCGGATCACCCACGCCGCCGTGCCGCCCAGC
GTGCTGCGCCAGCTGCCGGACGGGGACCTGCCGGACCTCGTGCACCTGGTGGTCGGCGGC
GAACCCTCGGACGACCGCCTGGTCGGCCGGTGGGCCCGCGACCGCCGGATGATGAACAT
GTACGGCCCCACCGAAACCTCCTGCGCCACCGGTGGCCGGCTGCTGCCCGGTGAGCCGGT
GACCATCGGCGGGCCGACCGACAACGTGGCGGTGTACGTCCTCGACGGCGACCTGCGGC
CGGTGGCGCCCGGGGTCGCGGGCGAGCTGTACATCGCCGGGCACGGCGTCGCCCGCGGG
TACCTCAACCAGCCGGGGCTCACCGCGGACCGGTTCCTCCCCTGCCCCTACGCCGAGCCC
GGCGCGCGGATGTACCGCACCGGGGACCGGGTGTTCCAGCGCGACGACGGCAGGCTGCA
GTTCCTCGGCCGCACCGACCACCAGGTCAAGATCCGCGGTTTCCGGGTGGAGCTCGGGGA
GATCGAGGCCGCGCTCGGCTCGGCTCCCGGCGTGGAGAACGCCATCGTGCTGGTGAAGG
GCACACGGTCCGGTGGGAAGCAGCTGGCGGGTTACGCGCTGCGGGCGCCGGGCGCGGCG
GTGGACACGGGCGGGCTGCGCCGCCGGTTGCGCGACGCGCTGCCCGAGCACATGGTGCC
GTCCTCGATCCTGGTGCTCGACTCCTTTCCCGTGCTGCCCAACGGAAAGGTGAACCGCGA
CGCGCTGCCGGACCCGGCGGGTTCCGGGCCCAGGGTCATCCAGCCCCCGGCGACGAAGG
TGCAGGCGGCGCTCTGCGGGATGTTCGCCGAAGTGCTCGGGGTGGCCGAGATCGGCGTGC
ACGACTCGTTCTTCGCCCAGGGCGGCGACAGCATCCTGGCGTTGCGCCTGGTCTCCGCCG
CCCGCGCGGCCGGTCTGGTGCTGTCCCCGCGGCAGGTGTTCGAGCACCCGACCGTCGCCG
AACTCGCCGAGGTGGTCGGGCGGGACGTGACCGCGGAGGCCGACGACGGCCTCGGCGCG
TTCCCCGCTCCCCCGATCGTGCGCTGGGCGACGGAACTGGGGCCGATCGACCGGTTCCAC
CAGTCGGTGTGCGTCCGCGTCCCGGCCGGGGCCGGGCACGACCGGCTCGCCAAGGTCCTG
CAGCTGGTGATCGACCGCCACCCGGCGCTGCGCACGCGGCTCACCGACGTGCGTTCGCTG
CGTGCCGACGCACCCGCCACGGCGGACGTGTTGTCCACTGTGGACGTCACAGGGCTCGAT
CTCGACGCCGTGGTCGAGGAACAGCGCGAGGCTGCCGTCGCCGCGCTGTGCCCGGCCGA
CGGCAGCCTCGTGCGCGCGGTGTGGTGCAACGCGGGCGAGGACCACCACGGCACGCTGC
TCCTGGTGCTGCACCACCTCGCCGTGGACGGGGTCTCGTGGCGCGTCCTGCTCGACGACC
TGGCGCACGCGTGGGAGGTGGTGGCCGCCGACGGGACCGCGCGGTGGGCACCGGCGGGC
ACGTCCCCGCGCACCTGGGCGAACCAGCTCGCCACCGCGGCGGCCGAACCCCGCTGGAC
GGCGCAGGCCGCGCTGTGGCAACGCGTCCTCACCACCGACGACCCGCCCATCGGCAAGC
GGGAACGCACGGGCACCGACACCTTCGACACCGCGGCCGCCACCGAACTCACGCTGCCG
GACGGCCTCGCCGAACCGCTGCTGACCGTGCTGCCCGACCGGTACCGGGCCACCCAGAAC
GACGTGCTGCTGACCGCGTTCGCGCTCGCCGCCCGGCGCTGGCGCGAACGGTGGCTGCCC
GGCGGCACCGCCGTGCTGGTGGACCTGGAAGGGCACGGCCGCCAGGACGCCGGGCAGCC
GGTCGCGCTGGACAACACGGTCGGCTGGTTCACCAGCATCCACCCGGTGGCCGTCGATCC
CGGGGCGCTCGACTGGCCCGCCGTGGTCTCCGGCGGCGCCGAGCTGGGCCCGGTGCTCAA
GCGGGTCAAGGAGCAACTGCGCGCGATCCCCGATCACGGCCTCGGTTTCGGGCTGCTGCG
GTACCTCAACGACGACACCGCTCGGGTGCTGGCCCCGCTCGGCTCGCCCCGGCTGGCCTT
CAACTACCTCGGCAGGCTCACCGTCTCCGAGGCCGCGGACTGGGCGCTCACCGGCGACGA
CCCGTTCGACGGCGCCGCCGACCCGCGCATGGTGATGCCGCACGAGCTGGAGATCAACGT
CCTCGCCTACGACCGTCCACAGGGGACGGAACTCACGGTGCGGGCGATGTGGCCGTCGG
GGGTGCTGGACGAAGCCGACGTCATCGACTTCCTCGGCCTCCTGCGGGACGCGCTGACCG
GGCTGGCCGCGCACGCCGCGCTACCCGGCGCGGGCGGAATGACCCCGTCCGACGTGCCG
CTGGTCGACCTCGACCAGGAGGAGATCGACGAGCTCACCCGGGACCGGCCGGGTACCGC
CGACATCCTGCCGCTGACCCCGCTGCAGGAGGCGCTCCTGCTGCACCACCTGGTCACCAA

GGACACCATCGACGTCTACAACGAGCAGTTGCGGATGATCCTCGAAGGGCCGCTGGACG
CGGACCGGCTGCGGGCCGCGCTGGCGGCGGTGGTCCGGCGGCACCCGGCGCTGGCCGCC
GCCTTCGACCACGACGTGGCCGAGCCCGTCCAGCTGGTGCCCGCCGACCCGCCGCCGGTG
CCGTGGACCGAGCACGACCTCTCGGACCTCGACCCCGGCGAGCGCGACCTCCGCGCGGC
GCACCTGGCCGACGCCGACCGGGCCGCCCGGTTCCGGCTGGACCAGCCACCGCCGCTGCG
GGTCCAGCTGGTCCGGCTCGGCCCGGACCGCCACCAGCTCGTGCTCACCGCGCACCACAT
CGTCTGGGACGGCTGGTCCATGGCGATCGTGCTGCGCGAGTTCTTCGCCTGCTACCCGCA
CGGCGACGACCGCGTGCTCCCCGCGCCGCCGCGCTACCGGAACTACCTGACCTGGCTGCG
CGCGCAGGACACCGGTGCCGCGCGCGCGGCGTGGGCGGACTACCTCGCCGGGGTGGCCG
GCCCGACGCGGGTCGCCCCCGACCTGCCTGCCGACCAGGTGACGCACGAACTGCTGATCA
CCCACCTGCCCGAGAACGTGGCCACGCGGCTCACCGAGCGGGCCAAGCAGGCCGGGGTC
ACCTTCAACGCGGTGGTGCAGCTGGTCTGGGCGGGCCTGCTCGGTGAACTCACCGGCGAG
CAGGACGTCATCTTCGGCACCTCGGTCTCCGGCAGGCCGCCGGAGATCGCCGGGGTGCAC
GACATGGTCGGGCTGCTCACGAACACCGTGCCGGTGCGGGTCCGGCTGGAGCACGAGGC
GCCGGTGGCCGACGCGCTGGCCCGGCTCGCGCGGGAGCAGGTCCCGCTGCTCAACCACC
ACCACCTCGGGCTGGCCGACATCCAGCGGCAGTCCGGCCACGACCGGGTCACCCTGTTCG
ACACCACCCTGATGGTGCTCAACTACCCGTTCGACCCCGCCGAATGGGACGCCGCCCTGG
GTGATCTGCACGTGGCGGACCACCACCTCAGCGACGACACCCCCTACCCGCTGCGACTCG
TCGTGGTGCCGGGGCCGCGGGTCCAGGTCCGGCTCGGCTTCCGGCCCGACGCCGTGAGCC
GGGAGGAGGCCACCGCCCTGCTCGACCGGGTGGCCGCCGCGTTCACCGCCATCGCGCAC
GACCCCGGGCTGACCGTCGCCGGCCTCTTCGCCCGAACACCACTTGTCGCGCCCCTCGAC
GGGCGGCTGGAGGGAACAACATGA

>ammD
ATGCGCAGCTCGGCCGTGCTCGAATTGTTCGGGTCGCGGGTTTCCCGCTCCCCGGACGCG
ATCGCCGTGCGTTCCGGGCGGGAGGCCGTGAGCTACGGCCAGCTGGACCTCGCCTCGGCA
CGGCTCGCCTTCCGCCTGGCCGCAGGCGGCGCCGGGCCGGGAAAAGTGGTGGCACTGCA
CCTGGACCGCGGTCCGGAGCTGGTGATCGGGCTGCTCGCCGTGCTCCGGACCGGTGCCGC
GTTCGTACCGCTGGACGCCGCTCATCCGGCCGGGCGGCTGCGGTGGCTGCTGGAGGATTC
GGGTGCCGATCAGGTCGTTTCGAATTCACCGCTGCCCGGCGGTTTCGGGAATTGCGCGGT
GGTTCCGGTGGTCGGCCCCGGCACGGAGTTGTTGCCGGAACGGCCGATCGATCCGGCGGC
CGCCGCCTACCTCATGTACACCTCCGGATCCACCGGTCCACCCAAGGGCGTGGTGGTCGA
ACACCGCCAATTGGCCTCTGTGTGCGCGGTGTGGGAAGAACTGTACCGGTTGCGTGAACG
TCCGCTGGAATTCGTTTCCGTGACCGGTTTCGCCACCGATCTGTTCTTCGCCGATTTCGCC
CGTTCGGTGCTGTACGGCGGGACGCTGATCCTCGCCCCTCGTGAAGCCATCACCGACCCC
GCGCGACTGCTCGACCTCATCGAGCACACGGGTGGCACCGCGCTCGAACTGCTGCCGTCG
CTCGCGAAGGCACTGGGCCAGGAAGCGGCACGGCGTGGTGGCATGCCACCGCTGGACCT
GGTGTCGGTGGGATCCGAGGCGTGGCCGAGTGGGGACGGCCGTGCCTTCCGCGCCCTGCT
GAGCCCGGAAACCCTGCTGTTCAACGCGTACGGCACCACGGAGACCACAGTGGACTCCT
GCGTGTACCGCGTGGCGTCACCGGATGATTGGCCGGGCTCGGTGCCGATCGGCCGCCCGG
TGCCCGGAACCACCGCCTACGTGCTCGACGAGGACCTGGCCGATGCCGAGACCGGTGAG
CTGTACCTCGGCGGCGAAGGCGTGGCGCGGGGATACCACCGGCGCCCCGGGCTCACCGC
CTCCCGCTTCCTGCCCGACCCGTTCCGCCCCGGCAAGACCATGTACCGCACCGGCGACCT
GGTCAGGCGCCAGGAGAACGGGGTGCTCGAACACCTGGGCCGGGTCGACGACCAGCTCA
AGATCCGCGGTTTCCGCGTCGAACCGGGAGAAATCGAGAACGTCCTGGTCCGGCACCCCG
CGGTGGACCGGGCGGTGGTCGCGTCACCGGACGAGCCGGGGCGCCGAAGACTCGTCGCC
TACTTCGTGCCCGTCGAAGCGGAACCGCCGGCGACGGCGCACCTTCGCGAGTTCCTGGCG
GCCAGGCTGCCGGAACACCTGGTGCCGACCGCGTTCGTCGCGCTGGAGCGCTTCCCGGTA
CTGCCCGGCGGGAAGGTCGATCGCCGCGCGCTGCCCGCTCCGCCGCGCACGGTCGAGGG
CGCGGAACCGCGCACCGAGTCGGAACGCGTTCTCGCCGGGATCTGGCGTGAGGTGCTCG
AACTCGACCACGTCGGTATCGACGACAACTTCTTCGATCTGGGCGGGGATTCGATCCTCG
GCATCCAGGTCGGCGCGCTCGCCAGGGCACGGCTCGGGGTGGTCTGGCCCTATCGCGCGT
TGTTCGACCGGCCGACCGTCGCGGAACTGGCGGCGCTGCCCGGTGAACCGGCGACGGAG
GTGCGCGTCGCGGAAGCGGGGGACCGGTTCCCCGTGTCGTTCGCGCAGCGGCGGCTCTGG
TTCCTGCACACGCACTCACCCGGCGCGGAGTACAACCTCCCGAAAGCGCTGCGGTTGCGC
GGCGAACTGGACGTCGACGCCCTGCGATCGGCCTTGACCGCGCTGGTGGAACGGCACGA

GATCCTGCGCACGAGCTTCCCGGTCGAGCGCGACGTGCCCTGGCAACGGATACACCCACC
GGCCCCGGCCGACCTCGAACTGGTCGACCTCGACGGGGAGCAGGGCCTGGACGATCTCCT
GCACGCCGAGGCGGAGGTGGTCTTCGACCTGGCCGAGCGCCCGCCGGTACGGCTGGTGCT
GGCGAAGCTCGGTCCCCGGGACCACGTCCTGGTGCTGAACCTGCACCACCTGCTGACCGA
CGACTGGACCAGCCAGATCCTGCTCGCCGAACTGGCGGAGCACTACCGCGCGGCGGTTAC
GGGCGAGGCGTCGCGCCTGCCGCCGCTACCCGCCCGATACGCGGATTTCGCACGCTGGCA
GGCCGAACACCTCACCCCTGAACTCGCCGACCGGCAGTTGTCCTTCTGGCGCACCCAGCT
AGCTGGGCTGCGCCCCTTCGAACTCCCACCGGACCGGCCGCGACCGGCCCGCCGCTCGGC
CGAGGGGGCGGCACACCGGACCGAGTTGTCCCCGGAACTGACCGCCCGGCTGGCCGAGT
TCGCCAGGACCCGCCGGGTCACCCTCTTCACCACCCTGCTCGCCGCGGTCAAGCTGGTGT
TCGCCCGGCTGGCCGGCGAGCCCGACGTGGCGGTCGCGACGGTGGAGTCCGGGCGGGAG
CGGGCCGAGTTCGCCGACGTGGCCGGCTTCTTCGTCGGCACCGTGGTGCTCAGATCCACA
GTGGACGAGGAACAAAGCTTCGACGGCCTGCTCGCGCGGGTGCGCGAAACCGTGCTGGC
CGCGATGGACAACGCCGACGTGCCGTTCGACCGCGTGGTCTCGGCGCTGGCCCCGAAACG
GGACCTGGCCGCCCTGCCGCTGGTCCGCACCGCGGTGGTGATGCAGAACGCGCCGGTGCG
CCACGGCGTCTTCCCCGGGTTGTCCGCCGACGAGGTGGACCTGCCGATGACCGCCGCGAA
CATCGACCTCAACACCGAGTTCCGGCTCGTCGGCGACCGGCTCCACCTCGTCGTCAGCTA
CCGCACGGACCTCTACGACCGGCGCACGATCGAACGCCTCACCGCACAGCTGACCGAAG
TGCTCGCGGCGGTGGTCGAGGAACCGGACCGTCCCCTGTGGACGATGCCGCTGACCACGG
CGCCCCTCGCGAAGCCCGTGTTCGGGCTCTCGCTGCCGGCGACCCCGATCCTGTTCGCCG
ACCTCGCCCGCCGGTACCCCGACGAGGTCGCCGTGGTCGCCGGTGTGGTTGAACTGACCT
ACCGCGAACTGGCCGAACGGGCCGAGGCACTGGCCGATCGCCTCGCCGAAATCGGCGCC
GGGCCGGAGGTCCCGGTGGCCGTCTGCCTGCGGCGCGGGATCGACCTGGTGGTGAGCGT
GTTCGCGGTGTTCCAGGCCGGCGCGGTGCTGGTACCGGTCGACCCGGGGCACCCGCCCGA
CCGGATCGCCTTCGTACTGGCCGACTCCCACGCGCTGGTCGTGCTCACCGACGAGGCGGG
CGCGGACCGGCTGCCCGGCGGCGCGCCGGTGCTGCGGGTCGACGGTGCCCAGCCGGTCG
CGCCGAAGCGCCGTCAAGTCGTTCCCCAGCCGGACAACCTGGCCTACGTCGAGTACACCT
CCGGCTCCACCGGGGTGCCGAAGGCGGTGATGGTCGAACACCGGTCGCTGGTCAACAGC
GCGGCGGACGCGCGTGTCCAGCTCGGCCTCGGGCCCGGCACGCGGATGCTGCTGCACTCA
CCGATCACCTTCGACTTCGGCCTCTGGCAGCTGCTGATGCCGCTGCTGTCCGGCGCGGCG
GTCTGCCTGAGCGAGGCGGGGGAGCGGGACGGCACATTGACCCTGGCCGAGCAGATTCG
CCGCGACCGGATCACCGTGGCCTCGCTGACCCCGGCCCTGCTGTCCACAGTGGATCCCAC
CGCGGTGCCCGGGCTGGAACTGGTGACCGTCGGCGGCGAGCAGTGCCCGGCCGAACTGG
CCAGGAAGTGGCTGGCGCACACCGCGTTCGGCAACTTCTACGGCCCGACCGAGGCCACCC
TGGCCTCCACCGGCCTGATGCTGGCCAGGGGCGCCGATCCGGGCCCGGGTGCGGCGATGC
CGATCGGCCCGCCCATCCAGGGCAACACCGGCTACGTGCTCGACCGGTACCTGCGCCCGG
TGCCGCCGGGCGTCGACGGTGAGCTGTACATCGGCGGCATCGGGGTGAGCCGGGGTTACT
TCGGCCGGCCCGGCCTGACCGCCGACCGCTTCCTGCCGGACCCGTTCGGCCCGCCCGGTT
CGCGGGTGTACCGCACCGGTGATCTGGTCCGCCGCCTCGATGACGGCTCCCTGGAGTTCC
GCGGCCGGATCGACCGGCAGGTCAAGGCCCGCGGGTTCAGGGTGGAGCCCGCCGAGATC
GAGGCGATGCTGACCGGGCTGCCCGAGGTCAGCGAGGCGGCGGTGATCGCCGACGACGG
TGGCCGCCTGCTCGCCTACGTGGTCGCGGAAACCCCGCTGGACGACGAACTGCTGCGGGC
GGAAACCGCACGGGTGCTCCCGGCGTACATGGTCCCGGCCGCCTTCGTCACCCTCCCCGA
ACTTCCCCTGACGGTGAACGGAAAGCTCGACCGGGCGGCGCTCCCGGTGCCGGAGGCAC
GCTCCCGGTGCAGCTACCGCGCGCCCGGAACCGAAGCCGAGGCGCGTCTCGCGGAGATC
TTCCGTGAGGTGCTCGACGTGGAACGCGTCGGCGTCGACGACAACTTCTTCGAACTCGGC
GGCGACTCGATCCTGAGCATCCGGGTGGCCGCGGGCGCGAACCGCGCGGGTCTCCGGCT
GACCTCGCGAGAGGTGTTCGAACGGCAGACTGTCGCCGAGTTGGCGGCCGGGCTGCCCG
CCGAACCGAGCCCGGTGGTGCGCGACGACACGGCCGTGTCGGGTGCGGTGGAACTCACC
CCCATCCAGCGGTGGTTCCTCGATACCTTCACCGTCCACCCCGGACAGTTCACCATGTCCC
GCTTCCTCGAACTCGCGCCGCGAGTCGACCGGGAGACCGTGCGGACAGCCGTGCTCGCTC
TCGTCGCCCACCACGACGCTCTGCGCCTCCGGTTCAGCCGGGAGCACGGGCAGTGGCTCC
TCGCGACCGAACCCGGGGAGGTCTTCCGCGCCCATGATCTGTCCGAAGTGGATGACGCCG
GTCAAGCGATGGACCGCCGGATGGCGGAGGCCCGCGCGGAACTCGACCTCGCCACCGGT
CCGCCGTTCCTGGCCGATCACTTCGACCTGGGGGAGGGGCGGCCACCCCGGCTGCTCCTG
ACCGTGCACCACTTCGTCGTCGACGGGGTCTCCTGGCGCATCCTGCTGGAAGACCTCCGC

ACCGCGTGCGCGCGGCGGGACCTCGGCCCCAAGACGACCTCGTTCCAGCAGTGGTCGCG
AATGCTCCGCGAGCAGGTGGAAGCGGGCGCGCTGGACGGCGAACTGCCCTACTGGACCC
GGGTCCACGAGCGCACCGCACCGCCGCTCCCGCTGGACGGCCCCGGCGGGCAAGGGCGC
ACCGAGACGATCACCGTCCGGCTGACCGCCGAGGAGACCGCGGCGCTGCTCCGCGAGGT
GCCCGGCGTCTACCGCACGCAGGTCAACGACGTGCTGCTCAGCGCGCTGTCGGTGGCCGT
GTCCGGCTGGACCGGCCACGATCGCGTGCTGCTGAACCTGGAGACCCACGGCCGGGAGC
AACTGCCCGGCCAGGCCGATCCGTCCCGCACGGTCGGCTGGTTCACCAGCCAGTTCCCCC
TGGTGCTCGACCTGCCGCGGGACCGGGACTGGCGCACCGTGCTGCGCTCGGTCAAGGAGC
AGGTGCGCGAGGTCCCCGGCCGCGGGCAGAACTACGACGCGCTGCGGTACCTGTCCGGG
GTGCTCGACGGCGGGCACCCCGCGGAGATCAACTTCAACTACCTCGGCCGGTTCGCCGAC
GCGGCCAACGAGCTGTACCTCCGCGAGTTGCCGGTCGTCGACGGCCCGCATCCCGAAGAA
GCACGCCCGTTCCCGCTGGAAGTCACCGGCGTCGTCGAGGCCGGGGAACTGGTGCTGCGC
TGGGACCATTCACCCGCGACACTGGCCGGCGCGACCGTGCGTGGCCTGGCCGAACACACC
ACCGACGCGCTGCGGGCGATCATCGCGCACTGCCGCGCCGACGGCGCCGGTGGTCGCAC
CCCGTCGGACTTCCCGCTGGCCGGGCTCGACCAGGCCGCGGTGGACCGGCTGGCCGGTGA
CGGACGGTCGGTGGAGGACGTCTACCGGCTCACCCCGATGCAGGCCGGGATGCTGTTCCA
CACCCTGGCCGGCGGCGACGAGGTCTACACCTGCCGGACGAGCGTGACCTTCGACCGCGT
GACCGATCCGGCCGCGTTCGCCGAAGCCTGGCAACTGGTGGCCGGTGCGGCCCCGGCGTT
GCGCAGCACCGTGCACTGGGCCGGGCTGCCCGAACCCGTCCAGGTGGTGCACCGGCCCG
CGCCACTGCCGGTGACCTGGCACGACTGGCGCGAACTCCCCGACGAGCGGGACCTCGTGC
GACGCCTGCGTGAGGACGACCAAGCCGCCGGACTGGACCTCACCGGCGCACCGCCGACC
CGGCTGGCGTTCGCCCGGCTGGCCGGCGACCGGGTGCACGTGCTGATGACCACGCACCAC
CTGTTCATCGACGGCTGGAGCCTGGCCAGGCTGCTCTCCGACGTGACCGCCGCGACGGAA
GCGCTGAGCCGGGGAGAACAGCCGGACCTGCCACCGCAACGCCCGCTCCGGGACTACTG
CGAATGGCTGGCGGCCCAGGACCACGATGCCGTCGCGGCCTACTGGGGCAAGGTGCTCG
ACGGTTTCCGCGAGCCGACCCCGTTGCCGTTCGACCGCCCGCCCGCGCCCGGACACCGCA
CCCGCCGGACCGGGACCAGGACGTTGCGGTGGAGCGAAGAGCGCACCACCGGACTCCAC
GACTTCGCCCGCCGCCACCGGCTCACCGCGCACACCGTGGTCCAGGCGGCGTGGGGCTTG
CTGCTGTCCCGGCACGGTGGCACCGGCGAGGTGCTGTTCGGCACCACGGTCTCGGTGCGC
CCGCCCGAACTCCCCGGCGCGGAGACGATCATCGGCCCGCTGATCAACACGCTGCCGGTC
CGCCTCACCGCGGATCCGGGGACGACCGTGCTGGACTGGCTCGCCGAGATCCAGGCCGG
GCAGGCCGAGGCCCGTGAACACGCCTTCTACCCGTTCTCCCGGCAGCAGGAGCTGACCGA
GGTGCCGCCGGGCGTGAACCTGTTCGACAGCACCATCGCCTTCGAGAACCACGCGGAGA
ACCCGTTCGAGGTGGATTTCGACGGCGATGACGTGACCAACTTCGCGCTCGGGCTGGTCG
TGTTGCCCGGTTCCTCGCTCCACTTCGAACTGTCCTACGACGAGGACCTGTTCGACGACGC
CACCGCGGAACGGCTGGTCGCCCGCCTGGACACCGCACTGCGCTCGCTGACCAGCGCGCC
GGAGCAGCCGCTGGCGGCCGTCCGGATCCTGCCCGACGCGGAACTGGACCTGCTCGGCC
GCTGGGGCGCGCCACTGCCGCCCGCTCGGCAGCGAACCGCGGCCGAACTCTTCGCCAAGC
AGGTGCGCCGCGACCCGGACGCCGTGGCGGTTTCCGGCGGCACCGACCTCACCTACCGAG
AGCTGGACGAGCGGGCGGGCAGGCTGGCGCACCGGCTCGGCGTGGGCCGCGAGTCCGTG
GTCGGGGTTTGCCTCGACCGCGGTGCCGACGCGCTCACCGGCATGCTCGCGGTGGCCAAG
GCCGGGGCCACCTGGCTGCCGATGGACCCGGCCCAGCCACCGGACCGGTTGCGGTGGCT
GGCCGAGGACGCCGGTGCGGCCGTGGTGCTGACCGACCGGAATTCGGCGGGACGGTTCC
CCCGCGAACTCCGGGTGGACGAGGTGCGGTGGGATCGCGGGCCGGTGCTAGACCGGCCG
TCCCGGCTCGACGACGCCGCCTACGTCAGCTACACCTCGGGCACCACCGGGCGGCCGAAG
GGCGTGGTGGTGCCGCACCGGGGACTGGCCGCACAGGTGTCGGCGCTGTCGAGATCTATC
GGTACCGGGCCGGGAAGCCGGGTGCTGCAGTGGCTTTCCCCCGCCTTCGACGCGTCCATC
CTCGAAGTGCTCGCCGCGTTGTTCACCGGCGCGACGCAGGTACCGGCGCCACCGGGCACC
CCGGTGACCGGGCTCGCGGAGGTGGCGCGGGCGCACGGCGTGACCCACGCGATGATGCC
GCCGTCGGTGCTGGCGGTGCTGCCGGAAACCGACTGGCCCGCCGAGGTCACCGTGCTGAC
CGGCGGCGAGGCGGTCCCGGCCGCGCTGGCCGCCCGGTGGTCGAAGGGGCGGCGGCTGG
TCAATTCCTACGGCCCGACAGAGGTGACCATCAGCCCGACGTTCGGCCGCCCACTGTCCG
GAGTGGACGCCCCGCCGATCGGCCTGCCGATCGAGGGCGCCGTGGTCCACCTGCTGGACC
CGGCCCTGCGCCCGGTCCCGGTGGGCGTGGTCGGCGAGATCTTCCTCGGTGGCGACGGCG
TCGCCCGCGGGTACCACCGCGCGCCGGGCGCGACCGCGGCGAGCTTCGTGGCCGATCCGT
TCGGTGAGCCGGGCGCCAGGATGTACCGCACCGGTGACCTGGCGCGGTGGTCCTCCGACG

GCGAGCTGTACTTCGCCGGTCGCGTGGACGCGCAGGTGAAGGTGCGCGGGGCACGCGTC
GAGCCGGGTGAGGTCGAAGCCGCGCTGCTGCGGCACCCCGAAGTGCGCGAGGCCGCGGT
GACGGCGGCGGGCACCGGCGCCGAACGGCGGCTGGTGGCCTGGATCGTGCCGCGGCACC
GGCCGGGGCCGACGGCGGGCGAACTCCGCGAGCACCTCGCGGCCATCCTGCCCGAGCAA
CTGCTGCCCGCCGAGTTCGGCGTGCTCGACCGGATGCCGCTGACCTCGCGAGGCAAGCTC
GACCGGGCGAAGCTGGCCGGTACCGCGGTGGCACCCGGCGCTGACTACCTGGCGCCCCG
CACGGACAGCGAACGGCTGGTGGCGCGGGCCTGGGCGACCGTGCTCGGCCGCGAGGAGA
TCGGCGTCCGGGAGAAGTTCTTCGAGGCGGGCGGGTCCTCGCTGACGCTGGTCCGGCTGG
CCGGGGAGTTCGCCCGGCTTGGGCGCACCGAACTGCCGGTCGCGGTGCTGCTGGAGCACC
CGACCATCGAAGCCATGGCGAAGCGCGTCGGCCAGGCACGACCGGCCGCGGACCACGAA
CTGTGA
